# Co-Extracellular Vesicles Delivery System Enhances Immunochemotherapy for Glioblastoma

**DOI:** 10.34133/research.1219

**Published:** 2026-03-31

**Authors:** Wenbo Niu, Rui Guan, Le Sun, Mingqing Wang, Xuejiao Wang, Biao Zhang, Xiangrong Hao, Qun Wu, Zhongman Cheng, Jiahui Wei, Ying Wang, Jian Zhang, Jun-Bing Fan

**Affiliations:** ^1^ Department of Oncology, Zhujiang Hospital, Southern Medical University, Guangzhou 510282, P. R. China.; ^2^ School of Traditional Chinese Medicine, Southern Medical University, Guangzhou 510515, P. R. China.; ^3^ Cancer Research Institute, School of Basic Medical Sciences, Southern Medical University, Guangzhou 510515, P. R. China.

## Abstract

In the tumor microenvironment, both tumor cells and tumor-associated macrophages (TAMs) frequently impede the effective treatment of glioblastoma (GBM). Herein, a co-extracellular vesicles (EVs) delivery system composed of M0 RAW264.7 macrophage-derived extracellular vesicles (MEVs) and doxorubicin (DOX)-loaded lemon-derived EVs (LEVDs) is demonstrated, enabling a significant enhancement of immunochemotherapy for GBM. This system facilitates the penetration of both EV types across the blood–brain barrier and blood–brain tumor barrier, enabling precise modulation of TAMs and tumor cells within the GBM microenvironment. During this process, MEVs exhibit a remarkable homing capacity toward TAMs, and meanwhile, they are enriched in microRNA let-7f-5p, which targets the 3′ untranslated region of A20 mRNA in M2 macrophages, leading to the activation of nuclear factor κB signaling pathway. This cascade drives the repolarization of M2 macrophages toward an M1 phenotype, effectively reversing the immunosuppressive tumor microenvironment. Concurrently, LEVDs exhibit exceptional targeting of GBM cells through receptor–ligand interactions, facilitating efficient chemotherapy. As expected, the co-EVs delivery system significantly enhances immunochemotherapy for GBM through MEVs-mediated TAM repolarization and LEVDs-driven chemotherapy.

## Introduction

Glioblastoma (GBM) is recognized as the most aggressive primary brain tumor owing to its high mortality and poor prognosis [1]. Two primary challenges persist in the clinical treatment of GBM. First, the blood–brain barrier/blood–brain tumor barrier (BBB/BBTB) severely restrict the drug delivery to brain tissues [2–6]. Second, the highly immunosuppressive tumor microenvironment, predominantly shaped by tumor-associated macrophages (TAMs), further complicates treatment [7,8]. These TAMs are mainly skewed toward an M2 phenotype and secrete large quantities of immunosuppressive cytokines, thereby inhibiting the recruitment and antitumor activity of cytotoxic T cells [9,10]. Consequently, both TAMs and tumor cells within the tumor microenvironment exhibit concurrent resistance, thus greatly impeding the successful treatment of GBM [11–17]. Therefore, dual targeting of TAMs and cancer cells seems to be a promising therapeutic strategy. To date, several attempts have been made to simultaneously modulate TAMs and tumor cells by incorporating immunomodulatory payloads and chemotherapeutics into a single nanoparticle or hybrid extracellular vesicles (EVs) platform [17–24]. However, such coencapsulation strategies frequently expose tumor-resident and systemic immune cells (including TAMs) to chemotherapeutics because macrophages are major nanoparticle sinks [25,26]. This nonspecific drug exposure can cause cytotoxicity or dysfunction of macrophages and other immune cells, potentially compromising immunomodulatory efficacy and disrupting antitumor immune crosstalk [27,28].

**Fig. 1.**
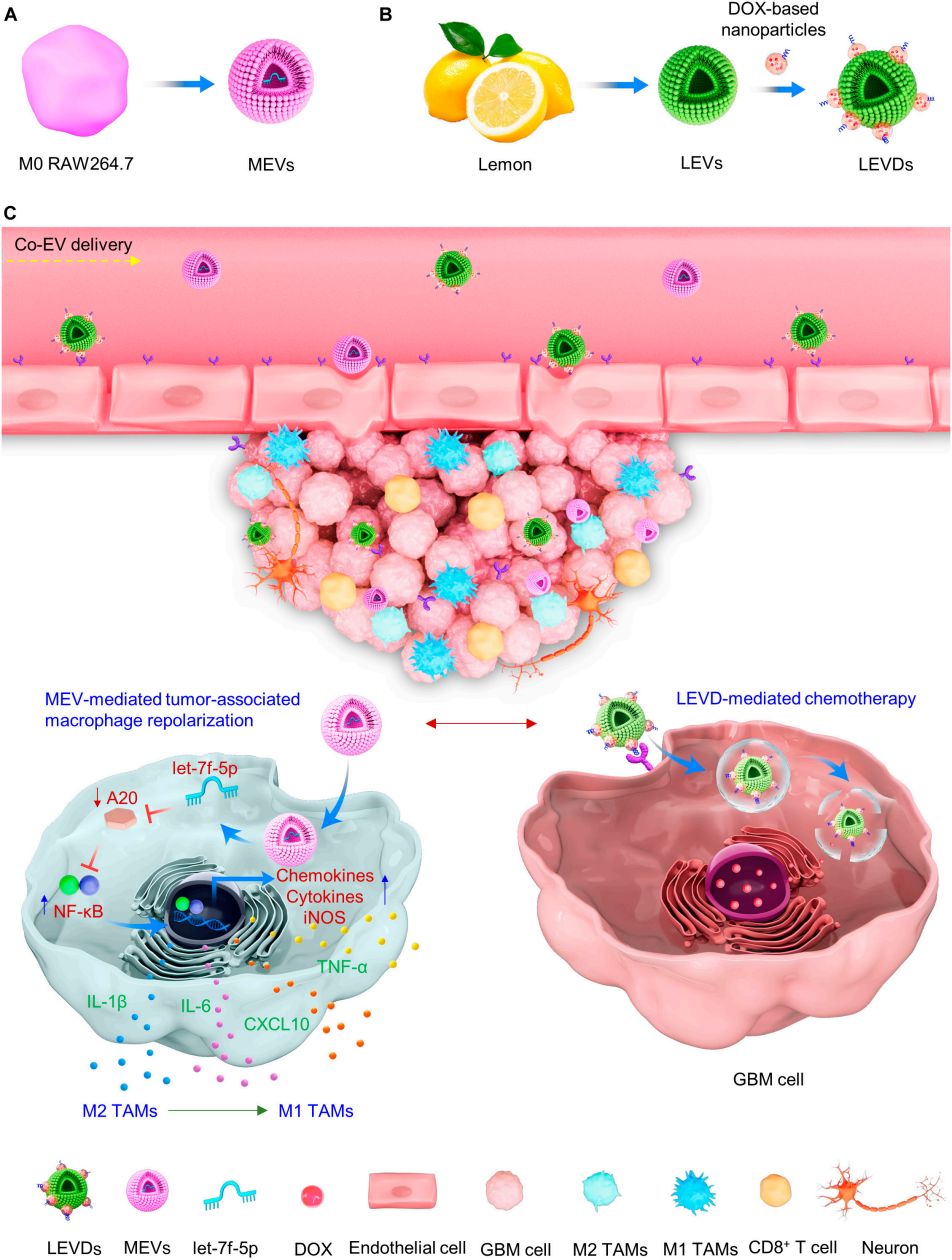
Schematic of the co-EVs delivery system for enhanced glioblastoma (GBM) immunochemotherapy. The co-EVs delivery system comprises M0 RAW264.7 macrophage-derived extracellular vesicles (MEVs) (A) and doxorubicin (DOX)-loaded lemon-derived extracellular vesicles (LEVDs) (B). During the delivery process, MEVs exhibit a remarkable homing capacity toward tumor-associated macrophages (TAMs) and enables the direct repolarization of protumor M2 macrophages into an antitumor M1 phenotype. This is achieved through the let-7f-5p within MEVs, which could target A20 within M2 macrophages, thereby activating the nuclear factor κB (NF-κB) signaling pathway. This process significantly reverses the immunosuppression of the GBM microenvironment. Concurrently, LEVDs demonstrate exceptional targeting of GBM cells through receptor–ligand interactions and facilitate the delivery of DOX into these cells, thereby enhancing chemotherapy effectiveness (C). As expected, the co-EVs delivery system significantly enhances immunochemotherapy for GBM through MEV-mediated M2 TAM repolarization and LEVDs-driven chemotherapy.

In living systems, diverse cells and their derivatives coexist, each carrying distinct information that synchronously and independently regulates life activities, which is crucial for efficient system function and overall health. This common phenomenon inspires us to develop a codelivery system capable of simultaneously and independently modulating TAMs and tumor cells within the tumor microenvironments. Herein, we demonstrated a distinct co-EVs delivery system that leverages 2 separate and independent EV populations, each for a specific cellular target. The co-EVs delivery system comprises: (a) M0 macrophage-derived EVs (MEVs) enriched with immunomodulatory microRNA (miRNA) let-7f-5p, which home to TAMs (Fig. 1A); and (b) cyclic arginine-glycine-aspartic acid (cRGD) peptides-functionalized, doxorubicin (DOX)-loaded lemon-derived EVs (LEVDs) engineered to specifically target GBM cells (Fig. 1B). This strategy ensures functional specialization: MEVs carrying let-7f-5p target A20 mRNA to activate the nuclear factor κB (NF-κB) signaling pathway, driving the repolarization of M2 macrophages to an M1 phenotype to reverse the immunosuppressive tumor microenvironment; LEVDs independently deliver cytotoxic DOX to tumor cells for efficient chemotherapy (Fig. 1C) [29,30]. By physically separating the immunomodulator and the chemotherapeutic agent, our co-EVs platform protects macrophages from chemotherapy-induced nonspecific cytotoxic effects, enabling simultaneous and synergistic remodeling of the tumor microenvironment and direct tumor eradication.

## Results

### M0 macrophage-derived EVs repolarized M2 macrophages to an M1 phenotype

**Fig. 2.**
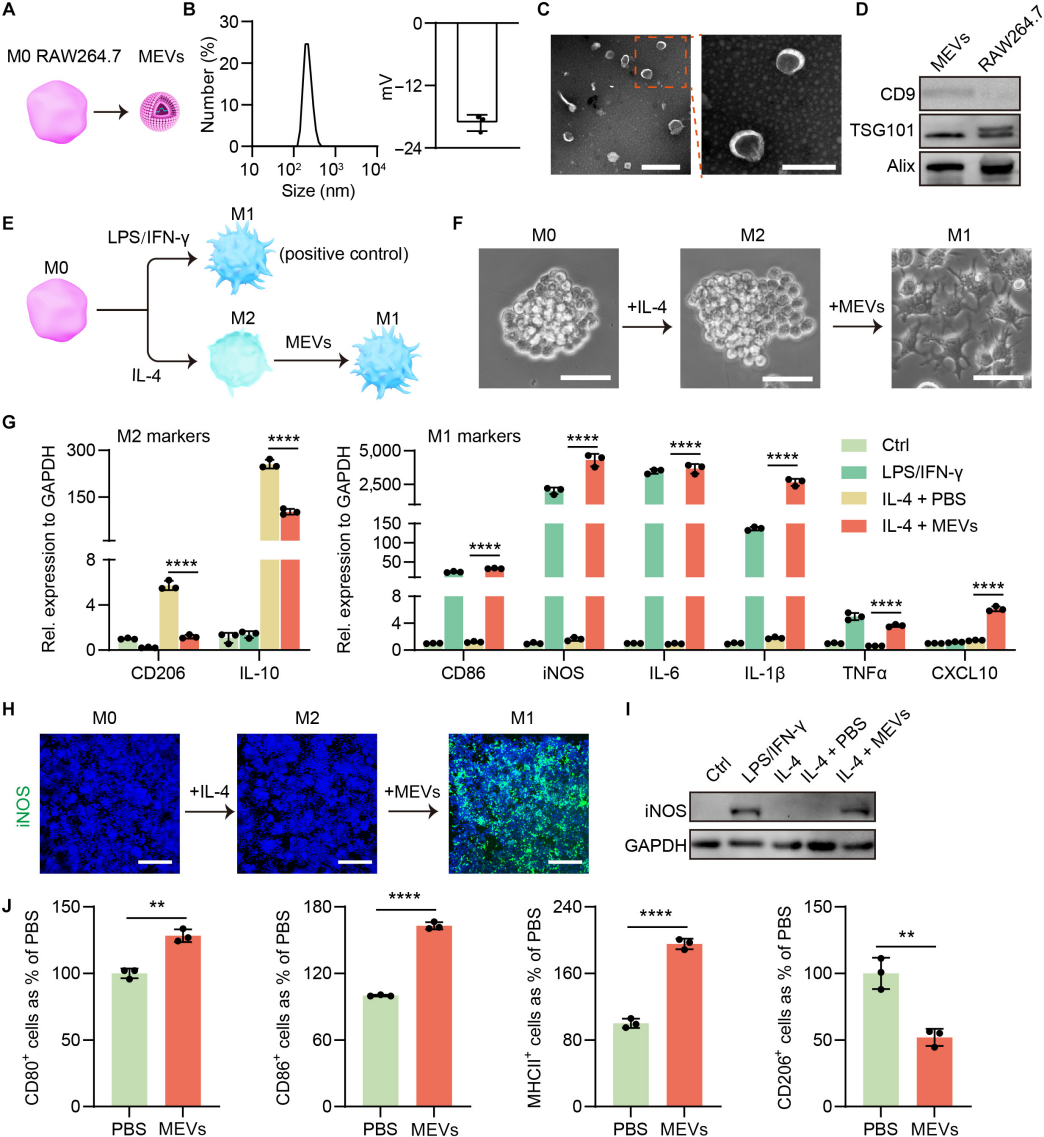
MEVs-mediated repolarization of M2 RAW264.7 cells into an M1 phenotype. (A) Schematic illustration of MEVs isolated from M0 RAW264.7 cells. (B) The average size and zeta potential of MEVs detected by dynamic light scattering (DLS). (C) Transmission electronic microscopy (TEM) images of MEVs. Scale bar: 500 nm for left panel and 200 nm for right panel. (D) The protein expression of MEVs. (E) Schematic illustration of the design of MEV-mediated macrophage repolarization. (F) Phase-contrast microscope images of M0, M2, and MEV-treated M2 RAW264.7 cells. Scale bar: 20 μm. (G) The markers of M2 phenotype (CD206 and interleukin-10 [IL-10]) and M1 phenotype (CD86, inducible nitric oxide synthase [iNOS], IL-6, IL-1β, tumor necrosis factor-α [TNF-α], and CXCL10) were detected by quantitative reverse transcription polymerase chain reaction (qRT-PCR) after treating M2 RAW264.7 cells with MEVs (*n* = 3). (H and I) The iNOS (a marker of M1 phenotypes) expression in MEV-treated M2 RAW264.7 cells. Scale bar: 300 μm. (J) Quantification of M1 and M2 macrophages by flow cytometry when M2 RAW264.7 cells treated with MEVs (*n* = 3). Data are represented as means ± SD. Statistical significance was determined using an unpaired, 2-sided Student *t* test (G and J), **P < 0.01; ****P < 0.0001. LPS, lipopolysaccharide; INF-γ, interferon-γ; GAPDH, glyceraldehyde phosphate dehydrogenase.

MEVs were first isolated from M0 RAW264.7 cells (Fig. 2A). Dynamic light scattering revealed that the hydrodynamic diameter and zeta potential of MEVs was 245.87 ± 21.38 nm and −19.20 ± 1.7 mV, respectively (Fig. 2B). Transmission electronic microscopy images showed that MEVs possessed lipid bilayers and exhibited a spherical morphology (Fig. 2C). Western blot analysis confirmed that MEVs were enriched in Alix, TSG101, and CD9, which are general EV markers (Fig. 2D). These results demonstrated successful isolation of MEVs from M0 RAW264.7 cells. We next investigated the cellular uptakes of MEVs in M0 RAW264.7 cells using PKH67-labeled MEVs (PKH67-MEVs). The results demonstrated that PKH67-MEVs were efficiently internalized by M0 RAW264.7 cells, with green fluorescence predominantly localized in the cytoplasm (Fig. S1).

Subsequently, we examined whether MEVs inherit the immunomodulatory capabilities of their parent M0 RAW264.7 cells. First, M0 RAW264.7 cells were induced into M1 and M2 phenotypes using lipopolysaccharide/interferon γ and interleukin-4 (IL-4), respectively. M1 RAW264.7 cells (induced by lipopolysaccharide/interferon γ) exhibited marked morphological changes, including alterations in size, lamellipodia, and filopodia, whereas M2 RAW264.7 cells (induced by IL-4) remained morphologically similar to M0 cells. Notably, exposure of M2 RAW264.7 cells to increasing concentrations of MEVs (Fig. 2E) induced morphological changes reminiscent of M1 cells, particularly at higher MEV concentrations, indicating that MEVs may repolarize M2 RAW264.7 cells toward an M1 phenotype (Fig. 2F and Fig. S2). We therefore assessed the expression of M1 and M2 markers in MEV-treated M2 RAW264.7 cells, using M1 RAW264.7 cells as a positive control. The quantitative reverse transcription polymerase chain reaction results revealed marked down-regulation of the M2 markers CD206 and IL-10, and significant up-regulation of the M1 markers CD86, inducible nitric oxide synthase (iNOS), IL-1β, IL-6, CXCL10, and tumor necrosis factor-α (TNF-α) (Fig. 2G). Immunofluorescence and western blotting confirmed that iNOS (a classical M1 marker) was absent in M0 and M2 RAW264.7 cells but strongly induced in MEV-treated M2 cells (Fig. 2H and I). Flow cytometry showed a decrease in CD206^+^ cells and an increase in CD80^+^, CD86^+^, or MHCII^+^ populations after MEV treatment of M2 RAW264.7 cells (Fig. 2J). To test the generality of this effect, we used EVs from mouse M0 bone marrow-derived macrophages (BMDMs) and human M0 THP-1 cells; similar repolarization was observed (Figs. S3 to S5). These data collectively demonstrate that M0 macrophage-derived EVs possess robust immunomodulatory activity capable of repolarizing M2 macrophages to an M1 phenotype.

### Mechanism underlying MEV-induced macrophage repolarization

**Fig. 3.**
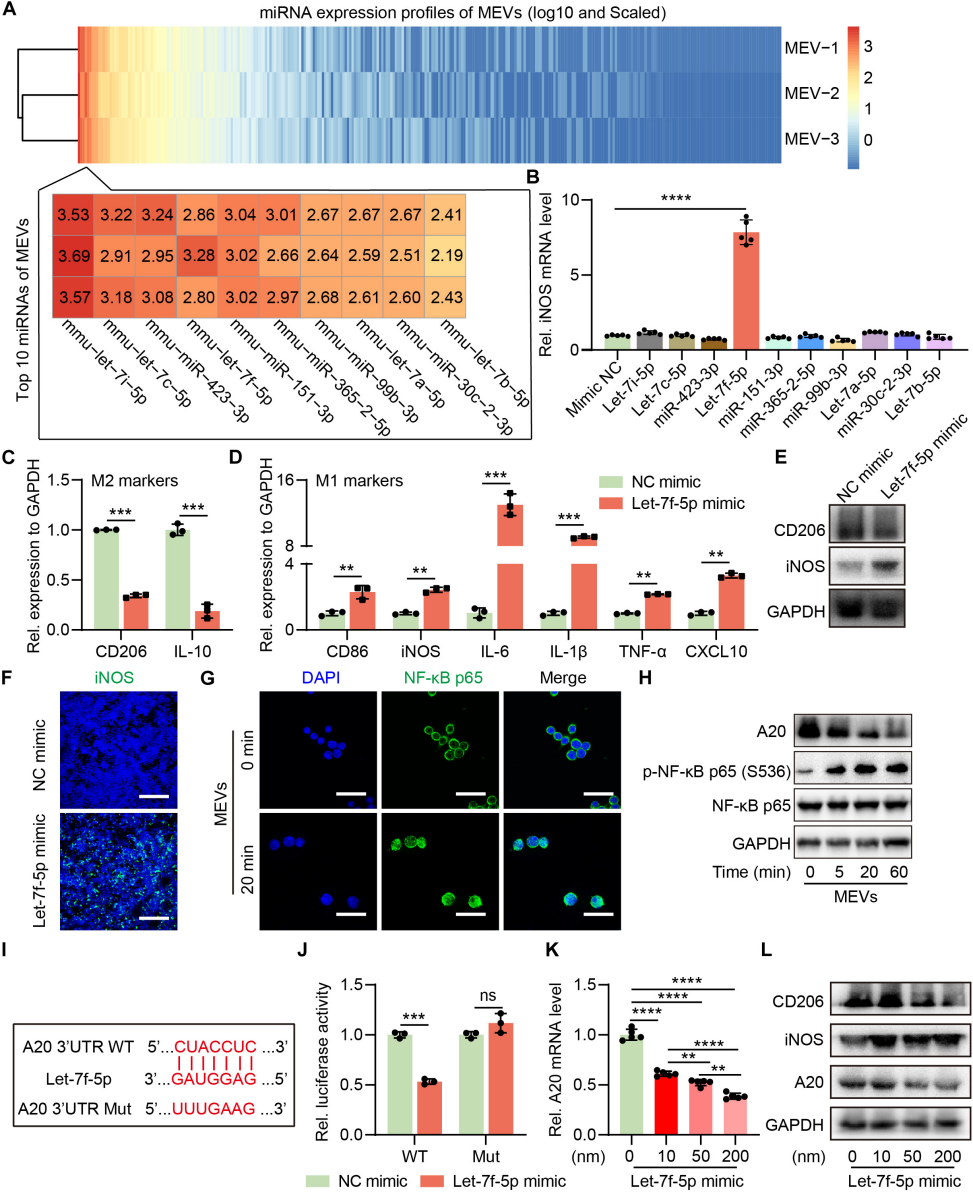
The mechanism of MEV-mediated M2 RAW264.7 cells repolarization. (A) Top 10 most abundant microRNAs (miRNAs) in MEVs detected by small RNA sequencing. (B) The mRNA expression of inducible nitric oxide synthase (iNOS) after M2 RAW264.7 cells transfected with the top 10 miRNAs, respectively (*n* = 3). (C and D) The marker expression of M2 phenotypes (CD206 and interleukin-10 [IL-10]) and M1 phenotypes (CD86, iNOS, IL-6, IL-1β, tumor necrosis factor-α [TNF-α], and CXCL10) was determined by quantitative reverse transcription polymerase chain reaction (qRT-PCR) after M2 RAW264.7 cells transfected with let-7f-5p mimic (*n* = 3). (E) The expression of CD206 and iNOS in M2 RAW264.7 cells transfected with let-7f-5p mimic assayed by western blotting. (F) The iNOS expression in M2 RAW264.7 cells transfected with let-7f-5p mimic detected by immunofluorescence. Scale bar: 300 μm. (G) Nuclear factor κB (NF-κB) p65 translocation into the nucleus in MEV-treated M2 RAW264.7 cells detected by immunofluorescence. Scale bar: 30 μm. (H) The expression of A20, p-NF-κB p65 (S536) and NF-κB p65 in MEV-treated M2 RAW264.7 cells with different incubation times determined by western blotting. (I) The wild-type and a mutated type of binding site between let-7f-5p and A20. (J) Relative luciferase activity of wild-type or mutated A20 luciferase report plasmid-transfected M2 RAW264.7 cells in the presence of let-7f-5p mimic (*n* = 3). (K) The mRNA expression of A20 in M2 RAW264.7 cells transfected with different concentrations of let-7f-5p mimic (*n* = 3). (L) The protein levels of CD206, iNOS, and A20 in M2 RAW264.7 cells transfected with different concentrations of let-7f-5p mimic. Data are represented as means ± SD. Statistical significance was determined using an unpaired, 2-sided Student *t* test (B, C, D, and J) or 1-way analysis of variance (ANOVA) (K), ns, not significant; **P < 0.01; ***P < 0.001; ****P < 0.0001. GAPDH, glyceraldehyde phosphate dehydrogenase.

EVs carry diverse bioactive cargoes, including lipids, proteins, and nucleic acids, among which miRNAs are the most abundant and can mediate intercellular communication and immune modulation [31–35]. We hypothesized that specific miRNAs in MEVs are critical for M2 macrophage repolarization. Thus, we performed deep sequencing of small RNAs derived from MEVs, identifying >300 miRNAs. As shown in Fig. 3A and Table S1, the 10 most abundant miRNAs present in MEVs were selected to identify potential targets involved in macrophage repolarization. The results suggested that only let-7f-5p significantly up-regulated iNOS expression in M2 RAW264.7 cells (Fig. 3B). To determine whether let-7f-5p was transferred from parent M0 cells to MEVs, we transfected M0 RAW264.7 cells with a Cy5-labeled let-7f-5p mimic, and the resulting MEVs were added to M2 RAW264.7 cells. Fluorescence in recipient cells confirmed that MEVs inherit let-7f-5p from their parent cells (Fig. S6). To further validate the role of let-7f-5p in repolarization, we transfected MEV-treated M2 RAW264.7 cells with a let-7f-5p inhibitor, which significantly reduced iNOS expression (Fig. S7). Conversely, transfection with a let-7f-5p mimic recapitulated the repolarization effect, down-regulating CD206 and IL-10 while up-regulating M1 markers (Fig. 3C and D). Western blotting and immunofluorescence assay confirmed these findings (Fig. 3E, F, and L). These results established let-7f-5p as a key miRNA driving M2 macrophage repolarization.

It is primarily acknowledged that miRNAs exert their biological functions by targeting the 3′ untranslated region (UTR) of mRNA to induce mRNA degradation or transcription repression [36,37]. We then used the target gene prediction algorithm TargetScan Mouse 8.0 to identify the target genes of let-7f-5p [38]. The results indicated that the seed sequence of let-7f-5p could interact with the 3’UTR of A20 (Fig. S8), which is a negative regulator of NF-κB activity [39,40]. NF-κB activation drives expression of numerous of proinflammatory cytokines (e.g., IL-6, IL-1β, CXCL10, and TNF-α) that support M1 polarization [41]. We therefore hypothesized that MEV-derived let-7f-5p targets A20, thereby activating NF-κB signaling and repolarizing M2 macrophages. Immunofluorescence showed nuclear translocation of NF-κB p65 in M2 RAW264.7 cells treated with MEVs, indicating NF-κB activation (Fig. 3G). Western blotting showed that NF-κB p65 was phosphorylated in Ser536 after treating M2 RAW264.7 cells with MEVs, while A20 expression was significantly inhibited, especially as the incubation time was extended to 60 min (Fig. 3H). These results suggested that MEVs could inhibit A20 expression and activate the NF-κB signaling pathway. Then, a luciferase reporter assay was conducted to detect the interaction between let-7f-5p and the 3’UTR of A20. The wild-type or mutated let-7f-5p-binding site in the 3’UTR of A20 was inserted into a luciferase reporter plasmid (Fig. 3I). The results showed that in the presence of the let-7f-5p mimic, when M2 RAW264.7 cells were transfected with the wild-type plasmid, the luciferase activity was significantly reduced. In contrast, the luciferase activity remained largely unaffected when these cells were transfected with the mutated plasmid (Fig. 3J). This suggested that let-7f-5p could inhibit A20 expression by directly binding its 3’UTR. Furthermore, it was also shown that let-7f-5p could inhibit the mRNA expression and protein level of A20 in a dose-dependent manner (Fig. 3K and L). Conversely, let-7f-5p inhibitor could restore A20 levels in MEV-treated cells (Fig. S9). Additionally, we also found that let-7f-5p mimic could suppress the CD206 expression while increasing the expression of iNOS in M2 RAW264.7 cells (Fig. 3L). These data demonstrated that let-7f-5p in MEVs targets A20 to activate NF-κB signaling, thereby reprogramming M2 macrophages toward an M1 phenotype.

### Biological activities of MEV-mediated macrophage repolarization in phagocytosis, proliferation, apoptosis, and colony formation

**Fig. 4.**
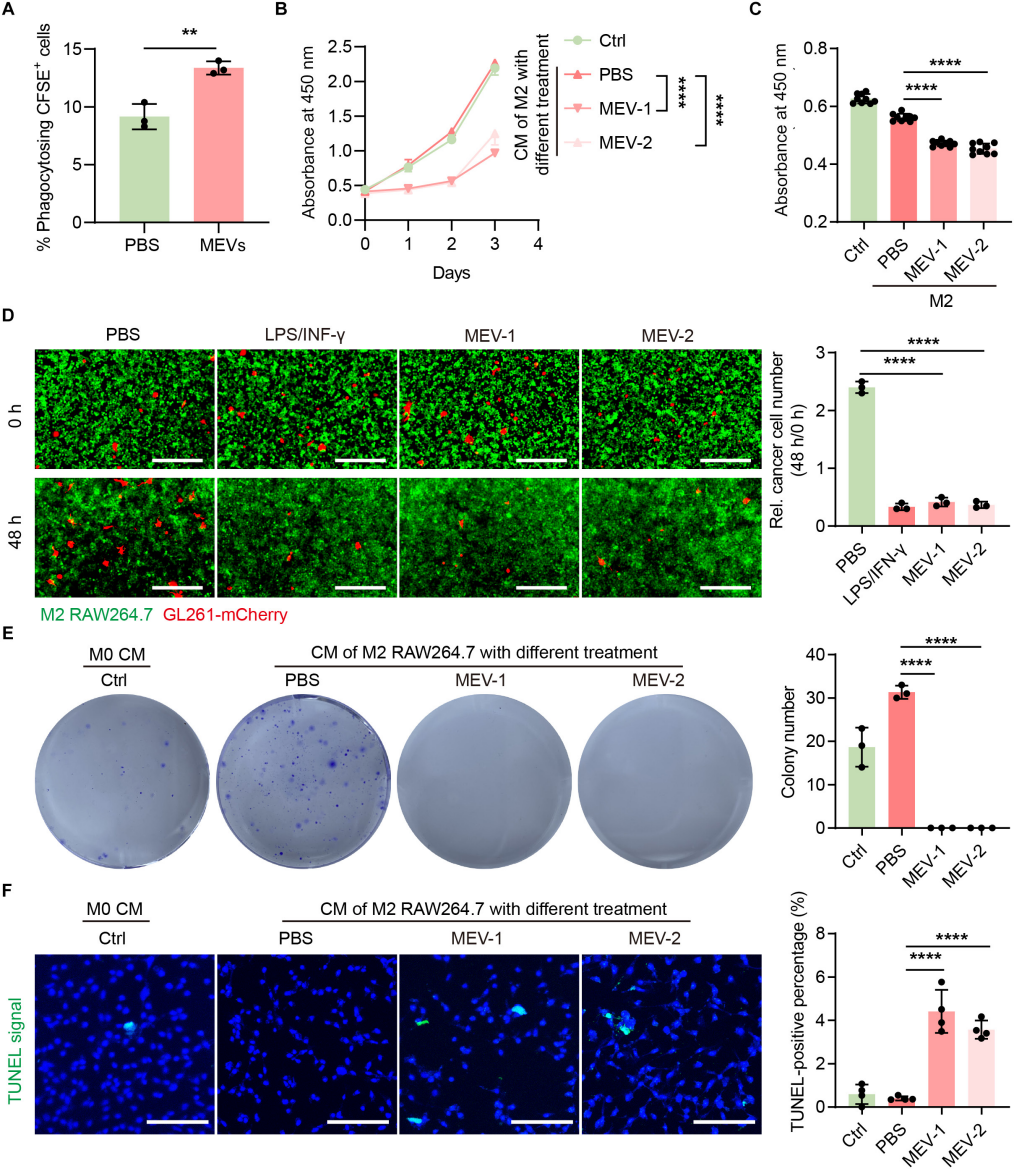
Anti-glioblastoma (GBM) effects of MEV-mediated M2 RAW264.7 cell repolarization in vitro. (A) The phagocytosis of GL261 cells by MEV-treated M2 RAW264.7 cells evaluated by flow cytometry (*n* = 3). (B) The proliferation of GL261 cells cultured with conditional medium (CM) of M2 RAW264.7 cells treated with phosphate-buffered saline (PBS) or different concentrations of MEVs (MEV-1: 10 μg·ml^−1^; MEV-2: 5 μg·ml^−1^) as indicated for 3 d assayed by Cell Counting Kit-8 (CCK8) assay (*n* = 3). (C) The antiproliferative effects of MEV-mediated M2 RAW264.7 cells reprogramming determined by CCK8 assay using a transwell noncontact coculture system. (D) Inhibition of GBM by MEV-mediated M2 RAW264.7 cell repolarization detected by a direct contact coculture system. Green: Carboxyfluorescein succinimidyl ester (CFSE)-labeled M2 RAW264.7 cells; Red: GL261-mCherry cells. Scale bar: 400 μm. (E) Colony formation of GL261 cells cultured with CM of M2 RAW264.7 cells treated with PBS or different concentrations of MEVs as indicated for 10 d (*n* = 3). (F) Images of terminal deoxynucleotidyl transferase dUTP nick end labeling (TUNEL) staining of GL261 cells cultured with CM of M2 RAW264.7 cells treated with PBS or different concentrations of MEVs as indicated for 36 h (*n* = 3). Scale bar: 100 μm. Data are represented as means ± SD. Statistical significance was determined using an unpaired, 2-sided Student *t* test, **P < 0.01; ****P < 0.0001.

The biological activities of MEV-treated M2 RAW264.7 cells in phagocytosis, proliferation, apoptosis, and colony formation were comprehensively investigated. We first investigated the cytotoxicity of MEVs on GBM cells and macrophages, and the results indicated that MEVs showed no cytotoxicity toward GL261 or RAW264.7 cells even at 100 μg·ml^−1^ (Fig. S10). M1 macrophages are typically recognized for their robust phagocytic capabilities. Thus, we next examined the phagocytosis of MEV-treated M2 RAW264.7 cells on GL261 cells. The result showed that MEV treatment resulted in an enhanced phagocytic activity of M2 RAW264.7 cells against GL261 cells (Fig. S11 and Fig. 4A). Furthermore, we also explored the antiproliferation capacity of the conditional medium (CM) of MEV-treated M2 RAW264.7 cells on GL261 cells. The results indicated that the CM of MEV-treated M2 macrophages could effectively inhibit the proliferation of GBM cells (Fig. 4B). The anti-GBM effects of MEV-mediated M2 RAW264.7 cells repolarization were further evaluated using transwell noncontact and direct contact coculture systems. The results revealed that MEV-treated M2 RAW264.7 cells demonstrated a marked antiproliferative activity against GL261 cells (Fig. S12 and Fig. 4C and D). In addition, we also found that the CM of MEV-treated M2 RAW264.7 cells could also effectively inhibit the colony formation (Fig. 4E) and promote apoptosis of GL261 cells (Fig. 4F). These results suggested that MEV-mediated macrophage repolarization could effectively inhibit GBM in vitro.

### MEVs penetrate the BBB/BBTB and target TAMs in vivo

**Fig. 5.**
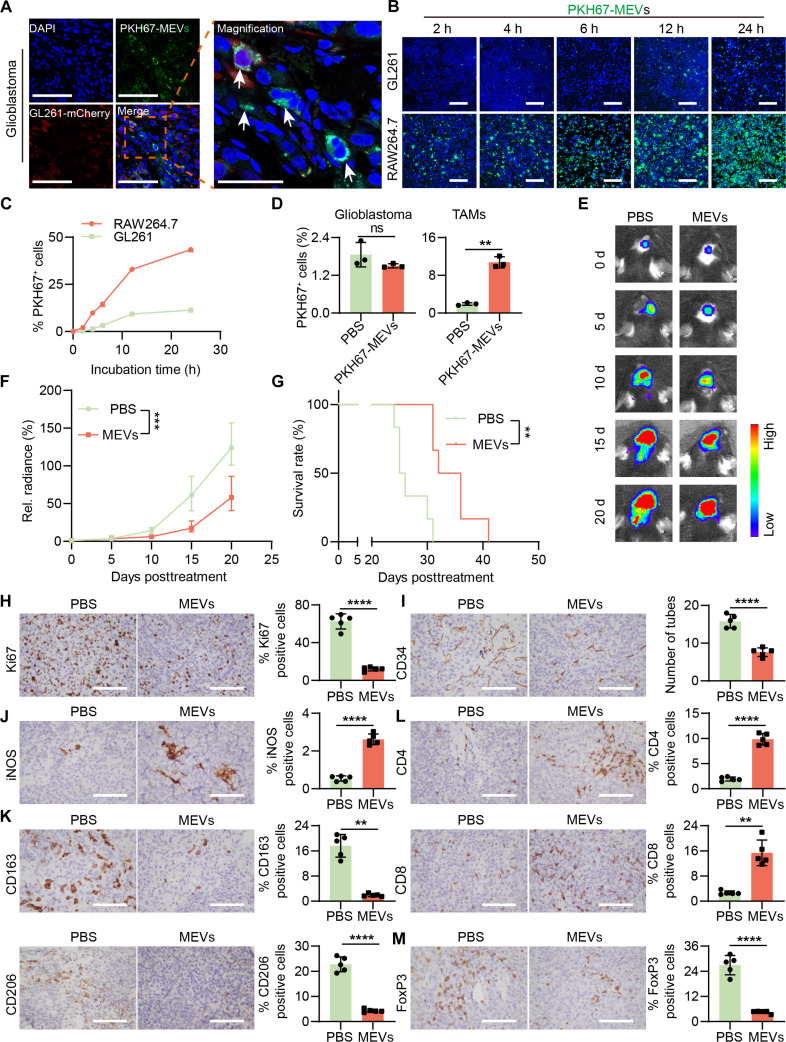
The blood–brain barrier/blood–brain tumor barrier (BBB/BBTB) crossing, targeting capacity, and anti-glioblastoma (GBM) efficacy of MEVs. (A) The BBB/BBTB crossing of MEVs. MEVs could bypass the BBB/BBTB and distribute in GBM in orthotopic GL261-mCherry-bearing mice. Scale bar: 100 μm for left panel and 40 μm for right panel. (B) The cellular uptake of PKH67-MEVs incubated with GL261 or RAW264.7 cells detected by laser scanning confocal microscope (LSCM). Scale bar: 300 μm. (C) The percentage of PKH67-positive cells after GL261 and RAW264.7 cells incubated with PKH67-MEVs determined by flow cytometry (*n* = 3). (D) The cellular uptake of PKH67-MEVs in GBM cells and tumor-associated macrophages (TAMs) in vivo (*n* = 3) detected by flow cytometry. (E) IVIS bioluminescent images of representative GL261-Luc-bearing C57BL/6 J mice treated with phosphate-buffered saline (PBS) and MEVs. (F) Relative bioluminescence of GL261-Luc tumor in different treatment groups (*n* = 6). (G) Kaplan–Meier survival curve of GL261-Luc-bearing C57BL/6 J mice in different treatment groups (*n* = 6). (H and I) Representative immunohistochemistry (IHC) images of Ki67 and CD34 in GL261-Luc tumors and the corresponding quantitative analysis (*n* = 5). (J and K) Representative IHC images of M1 markers (inducible nitric oxide synthase[iNOS]) and M2 markers (CD163 and CD206) in GL261-Luc tumors and the corresponding quantitative results (*n* = 5). (L and M) Representative IHC images of CD4^+^ and CD8^+^ T cells and regulatory T cells (Tregs) (FoxP3^+^) in GL261-Luc tumors and the corresponding quantitative results (*n* = 5). Scale bar for (H) to (M): 100 μm. Data are represented as means ± SD. Statistical significance was determined using an unpaired, 2-sided Student *t* test (D, F, and H to M) or 2-sided log-rank Mantel–Cox tests (G), **P < 0.01; ***P < 0.001; ****P < 0.0001.

An in vitro BBB model was constructed according to our previous study [29]. The bEnd.3 cells were cultured on the upper transwell chamber membranes coated with collagen I. When transendothelial electric resistance values of bEnd.3 monolayers exceeded 200 Ω·cm^2^, GL261 cells were seeded and cultured onto a slide in the lower chamber (Fig. S13A) [29]. The successful construction of the in vitro BBB model was confirmed by detecting the expression of tight junction proteins, such as ZO-1 and Claudin-1 in bEnd.3 cells, as well as the cell–cell adhesion protein (PECAM-1) through immunofluorescence (Fig. S13B). The results demonstrated that PKH67-MEVs readily traversed the endothelial layer and accumulated in GL261 cells (Fig. S13C). We further assessed the transcytosis of MEVs using a coculture experiment. The results demonstrated that MEVs could also be transported from bEnd.3 to GL261 cells via transcytosis (Fig. S14). Next, the capability of MEVs to bypass the BBB/BBTB and biodistribution in vivo were examined using an orthotopic GBM-bearing C57BL/6 J mouse model. A single dose of PKH67-MEVs was intravenously administered into orthotopic GL261-mCherry-bearing mice. After being perfused with phosphate-buffered saline (PBS), the liver, spleen, heart, lung, kidney, and GL261-mCherry-bearing brain, as well as the stomach and intestine, were excised and sectioned to assess MEV accumulation. It revealed a pronounced distribution of MEVs within the GBM tissues (Fig. 5A). Besides, MEVs could also be observed in the liver, spleen, lung, stomach, and intestine (Fig. S15). To further evaluate the biodistribution of MEVs, we administered Cy7-labeled MEVs (Cy7-MEVs) to intracranial GL261-Luc-bearing mice. After administration, these mice were sacrificed to assess the accumulation of Cy7-MEVs in key organs using an in vivo imaging system (IVIS) at 3 and 24 h. It showed that at 3 h postinjection, Cy7-MEVs predominantly localized in the liver, spleen, lung, kidney, stomach, and intestine, aligning with the results from the laser scanning confocal microscope. However, by 24 h, there was an marked decrease in the fluorescent signal of Cy7-MEVs across these organs. Notably, Cy7-MEVs persisted in GBM tissues even after 24 h postadministration (Fig. S16).

To assess MEV targeting to macrophages, we compared uptake by GL261 and RAW264.7 cells. PKH67-MEVs were taken up significantly more by RAW264.7 cells than by GL261 cells, as shown by confocal microscopy and flow cytometry (Fig. 5B and C). Furthermore, we validated the in vivo targeting capability of MEVs to TAMs in GL261-mCherry-bearing mice. Flow cytometry analyses confirmed that PKH67-MEVs were predominantly internalized by macrophages rather than GBM cells (Fig. 5D and Fig. S17). These observations suggest that MEVs could effectively traverse the BBB/BBTB, demonstrating remarkable targeting affinity for TAMs.

### MEVs suppress GBM growth and reverse the immunosuppressive microenvironment

We next evaluated the anti-GBM effect of M0 macrophage-derived EVs in vivo. The low yield of EVs derived from M0 BMDMs poses a considerable challenge for in vivo studies, as obtaining sufficient EVs for a single recipient mouse requires 10 to 15 C57BL/6 J mice. To address this, MEVs were used in subsequent in vivo anti-GBM assays. We first detected whether MEVs could induce M2 BMDM repolarization. The quantitative reverse transcription polymerase chain reaction results showed that M2 markers CD206 and IL-10 were remarkably down-regulated, while M1 markers CD86, iNOS, IL-1β, IL-6, TNF-α, and CXCL10 were clearly up-regulated in MEV-treated M2 BMDMs (Fig. S18A). In subcutaneous GL261 tumors, intratumoral MEV injection increased iNOS^+^ cells and decreased CD163^+^ or CD206^+^ cells (Fig. S18B). These results indicated that MEVs could reprogram M2 TAMs to an M1 phenotype in vivo. Thus, we used MEVs for the in vivo anti-GBM experiments. The orthotopic GL261-Luc-bearing mice were constructed, and the mice were administered either with MEVs or PBS at 5-d intervals, culminating in a total of 6 injections. The results showed that compared to the PBS group, GL261-Luc-bearing mice treated with MEVs exhibited a slower tumor proliferation rate (Fig. 5E and F) and longer median survival time (34 d for the MEV group versus 25.5 d for the PBS) (Fig. 5G), suggesting that MEVs efficiently inhibited GBM growth. The mice treated with MEVs also maintained higher body weight compared with the PBS treatment group (Fig. S19). We also performed immunohistochemistry (IHC) on GBM sections after administration to detect the expression of cell proliferation marker Ki67 and angiogenesis marker CD34. The results revealed that compared with the PBS group, a significant expression reduction of the Ki67 and CD34 was observed in the MEV treatment group, suggesting that MEVs could efficiently suppress the proliferation and angiogenesis of tumor cells (Fig. 5H and I). The markers of immune cells in GBM were also assessed by IHC, which showed increased iNOS and decreased CD163/CD206 in MEV-treated GBM (Fig. 5J and K). Furthermore, MEVs increased the infiltration of CD4^+^ and CD8^+^ T cells (Fig. 5L) and reduced the abundance of Tregs (FoxP3^+^) (Fig. 5M). All these data demonstrated that MEVs repolarize M2 TAMs to an M1 phenotype, counteracting the immunosuppressive microenvironment and benefiting GBM immunotherapy.

### The co-EVs delivery system (MEVs/LEVDs) enhanced immunochemotherapy for GBM

Although MEV-mediated M2 macrophage repolarization showed encouraging results in GBM treatment, therapeutic outcomes required further improvement. To simultaneously and independently regulate TAMs and tumor cells for improving the efficacy of GBM treatment, we developed a co-EVs delivery system comprising MEVs and DOX-loaded LEVDs. LEVDs were constructed through a 2-step process leveraging the chemical functional groups of both components according to our previous protocol. First, DOX-loaded heparin-based nanoparticles (DNs) modified with cRGD peptides were synthesized via self-assembly, utilizing carboxylated heparin as the polymeric backbone. This design capitalizes on heparin’s natural biocompatibility and its abundant carboxyl groups, which serve as reactive handles for subsequent conjugation. In parallel, lemon-derived EVs (LEVs) were isolated, which inherently present active amino groups on their surface, contributed by membrane proteins and phosphatidylethanolamine lipids. The final LEVD formulation was achieved by patching DNs onto the LEV surface. This integration is driven by the formation of stable amide bonds between the carboxyl groups (–COOH) of DNs and the available amino groups (–NH₂) on the LEV surface [29]. Transmission electronic microscopy images confirmed the vesicle structure of LEVs and LEVDs (Fig. S20A). The hydrodynamic diameters of LEVs and LEVDs were 211.47 ± 10.38 and 246.83 ± 17.92 nm (Fig. S20B); zeta potentials were − 39.17 ± 1.70 and − 32.67 ± 2.59 mV, respectively (Fig. S20C). The ultraviolet–vis spectra showed a DOX-characteristic absorbance peak at 489 nm for LEVDs (Fig. S20D), indicating that DOX was successfully loaded into LEVs. To rigorously evaluate the drug loading performance, we prepared 3 batches of LEVDs, and the drug loading efficiency and loading capacity were calculated. As detailed in Table S2, LEVDs demonstrated a DOX loading efficiency of 85.3% ± 2.1% and a loading capacity of 12.5% ± 0.8%, respectively. To better illustrate the drug loading capacity of the LEVs, we also calculated the amounts of DOX and cRGD loaded per microgram of LEVs. The results showed that 1 μg (protein amount) of LEVs loaded 1.54 ± 0.16 DOX and 0.40 ± 0.04 μg of cRGD in LEVDs (Table S3). To evaluate the stability of LEVDs in vivo, the PBS solution containing 50% fetal bovine serum was used to simulate the blood environment. It showed that the size of LEVDs did not significantly change in blood-mimicking conditions for 96 h (Fig. S20E). The release curve also displayed that the release rate of DOX from LEVDs was approximately 85% at pH 5.0, compared to only about 25% at pH 7.4 after 12 h of incubation (Fig. S20F). These results indicated that LEVDs were relatively stable under physiological conditions and exhibited pH-responsive release behavior. Furthermore, the DOX could be delivered into the nucleus by LEVDs, while LEVs was mainly located in the cytoplasm after internalization (Fig. S20G). We next investigated whether LEVs could also induce macrophage repolarization. It showed there was no obvious changes in the marker expression of both M1 and M2 phenotypes in LEV-treated M2 RAW264.7 cells, indicating that LEVs could not trigger repolarization in M2 RAW264.7 cells (Fig. S21). We also demonstrated that GBM cells and cerebrovascular endothelial cells were characterized by high expression of integrin αV and β3 (Fig. S22). Therefore, we next investigated the targeting ability of LEVDs for GL261 and RAW264.7 cells. The laser scanning confocal microscope images and the results of flow cytometry showed that the cellular uptake ability of LEVDs in GL261 cells was greater than that in RAW264.7 cells, indicating that LEVDs have good targeting capacity for GL261 cells (Fig. S23A and B). In addition, the uptake capacity of LEVDs in GBM cells was better than that of free DOX (Fig. S24), exhibiting good antiproliferation capacity (Fig. S25). The BBB/BBTB penetration capacity of LEVDs was also evaluated. In the in vitro BBB model, LEVDs could effectively traverse the endothelial layer to deliver DOX to GL261 cells (Fig. S26A). The results of in vivo experiments demonstrated that no fluorescent signals from DOX, DNs, or LEVDs were detectable in the brain tissue of healthy mice, confirming the restricted drug entry of the intact BBB. In contrast, in GBM-bearing mice, minimal signals of DOX and DNs were observed within the tumor tissue, suggesting limited extravasation due to the compromised BBB. Notably, strong LEVD fluorescence was detected in the tumor region, indicating that the targeting capability of LEVDs confers an excellent ability to traverse the BBB/BBTB to deliver DOX to GBM cells (Fig. S26B).

**Fig. 6.**
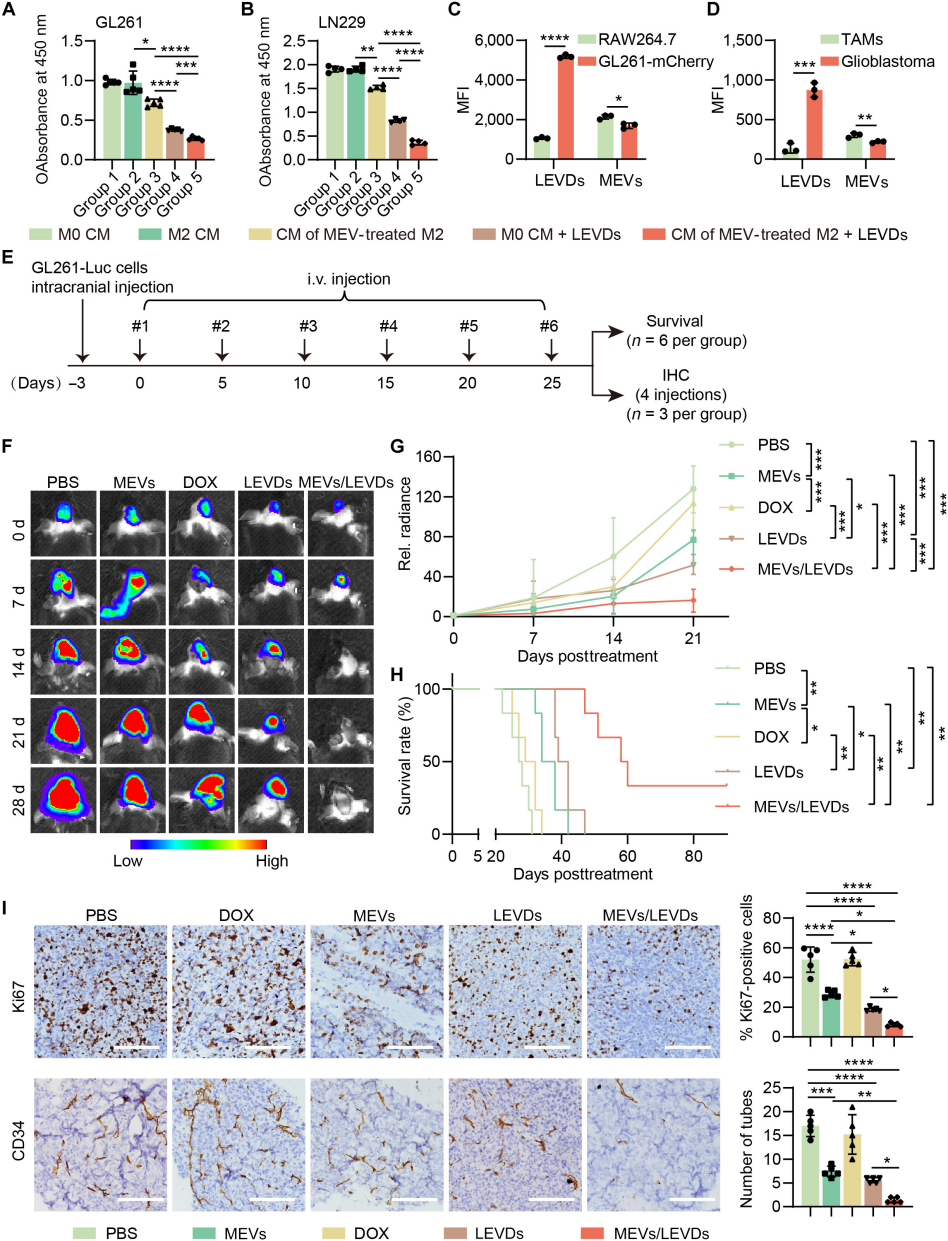
The anti-glioblastoma (GBM) efficacy of co-EVs delivery system (MEVs/LEVDs ). (A and B) The proliferation of GL261 and LN229 cells treated as indicated (*n* = 5). (C) Cell uptake of MEVs/LEVDs in GL261-mCherry and RAW264.7 cells, respectively, in the coculture system (*n* = 3). (D) Cellular uptake of MEVs/LEVDs in orthotopic GBM cells and tumor-associated macrophages (TAMs) from GL261-mCherry-bearing mice (*n* = 3). (E) Schematic illustration of experimental design for evaluating anti-GBM efficacy of MEVs, doxorubicin (DOX), LEVDs, and MEVs/LEVDs. (F) Bioluminescent IVIS images of representative GL261-Luc-bearing C57BL/6 J mice in different treatment groups. (G) Relative bioluminescence of GL261-Luc tumor in different treatment groups (*n* = 6). (H) Kaplan–Meier survival of C57BL/6 J mice in different treatment groups (*n* = 6). (I) Representative images of Ki67 and CD34 in GBM tissues from different treatment groups (*n* = 5). Scale bar: 100 μm. Data are represented as means ± SD. Statistical significance was determined using 1-way analysis of variance (ANOVA) followed by Bonferroni’s post hoc test (A, B, G, and I), an unpaired, 2-sided Student *t* test (C and D), or 2-sided log-rank Mantel–Cox tests (H), *P < 0.05; **P < 0.01; ***P < 0.001; ****P < 0.0001.

The anti-GBM efficacy of the co-EVs delivery system (MEVs/LEVDs) in vitro was subsequently explored. The results demonstrated that the combination of CM from MEV-treated M2 RAW264.7 cells and LEVDs synergistically inhibited GBM cell proliferation and induced apoptosis, compared to either treatment alone (Fig. 6A and B and Figs. S27 and S28). We also demonstrated that the co-EVs delivery system (MEVs/LEVDs) could efficiently penetrate the BBB in the in vitro BBB model (Fig. S29). We next assessed the targeting efficacy of the components within the co-EVs delivery system (MEVs/LEVDs) toward macrophages and GBM cells, respectively. An in vitro coculture assay revealed that LEVDs were preferentially taken up by GL261-mCherry cells, while MEVs were taken up by RAW264.7 cells (Fig. S30 and Fig. 6C). Similar selective targeting was observed in vivo in orthotopic GL261-mCherry-bearing mice (Fig. S31 and Fig. 6D). In contrast, no accumulation of MEVs or LEVDs was observed in the brain tissue of healthy mice, indicating that the intact BBB restricted brain penetration (Fig. S32). The anti-GBM efficacy of the co-EVs delivery system (MEVs/LEVDs) was subsequently evaluated. To optimize the MEV/LEVD ratio for in vivo therapy, we tested 3 co-EVs formulations with fixed LEVDs (DOX concentration 2.5 μg·g^−1^) and escalating MEV doses (1, 2.5, or 5 μg·g^−1^) according to the weight of mice. The GL261-Luc-bearing C57BL/6 J mice were administered with MEVs, DOX, LEVDs, and co-EVs delivery system (MEVs/LEVDs) and PBS (control) via intravenous injection at a 5-d interval for a total of 6 injections (Fig. 6E). The results showed that MEVs and LEVDs alone moderately inhibiting tumor growth. Compared to PBS, MEVs, DOX, and LEVDs, the co-EVs delivery systems of MEVs (2.5 μg·g^−1^)/LEVDs and MEVs (5 μg·g^−1^)/LEVDs exhibited significant suppression of tumor growth (Fig. 6F and G and Figs. S33A and B and S34). The overall survival results indicated that mice treated with MEVs (2.5 μg·g^−1^)/LEVDs and MEVs (5 μg·g^−1^)/LEVDs exhibited longer survival time compared with the other groups (Fig. 6H and Fig. S33C). The order of median survival time was as follows: MEVs (5 μg·g^−1^)/LEVDs (58 d) > MEVs (2.5 μg·g^−1^)/LEVDs (43 d) > LEVDs (39 d) > MEVs (1 μg·g^−1^)/LEVDs (38 d) > MEVs (34 d) > DOX (29 d) > PBS (27 d). Notably, 33.3% (2/6) of mice in the MEVs (5 μg·g^−1^)/LEVD treatment group achieved complete response, indicating great therapeutic advantages over monotherapy. Hence, MEVs (5 μg·g^−1^)/LEVDs (hereafter MEVs/LEVDs) were selected for further study. In the late treatment period, mice treated with MEVs/LEVDs maintained higher body weight than other treatment groups (Fig. S33D). The IHC results also indicated that MEVs/LEVDs achieved the best inhibition effect on the proliferation (Ki67) and angiogenesis (CD34) (Fig. 6I). Additionally, the biosafety was assessed in healthy C57BL/6 J mice. The co-EVs delivery system MEVs/LEVDs did not affect the body weight of mice (Fig. S35A) or serum concentrations of alanine transaminase, aspartate aminotransferase, alkaline phosphatase, or urea (markers of liver or kidney damage) (Fig. S35B). Given that the payload of co-EVs delivery system is DOX, a chemotherapeutic agent known to induce cardiotoxicity, we therefore focused on evaluating the cardiotoxic profile of this system. Echocardiographic analysis revealed that, compared with the control group, MEVs/LEVDs did not induce cardiac dysfunction, as evidenced by no significant difference in the left ventricular ejection fraction and left ventricular fractional shortening between the 2 treatment groups (Fig. S35C). Furthermore, no significant differences were observed between the 2 treatment groups in either the markers of myocardial injury (creatine kinase, creatine kinase-myocardial band, and cardiac troponin T) or the heart weight/body weight ratio, which was usually used as an indicator of cardiac atrophy (Fig. S35D and E). Hematoxylin and eosin staining results of the hearts, livers, spleens, lungs, kidneys, and brains also showed no signs of toxicity (Fig. S35F). We also detected the serum concentration of IL-6, IL-1β, and TNF-α to evaluate the immune-related inflammatory responses. As shown in Fig. S36, mild increases in serum IL-6 and IL-1β were observed in the MEV/LEVD treatment group. Although there was a trend toward elevation in the MEV treatment group, no statistically significant difference in TNF-α levels was observed between the 2 groups. The results indicated that the immunomodulatory effect of MEVs/LEVDs is targeted and controlled, rather than inducing a nonspecific cytokine storm. All these results indicated that MEVs/LEVDs possess excellent biological safety.

### The co-EVs delivery system (MEVs/LEVDs) reversed the GBM immunosuppression microenvironment

**Fig. 7.**
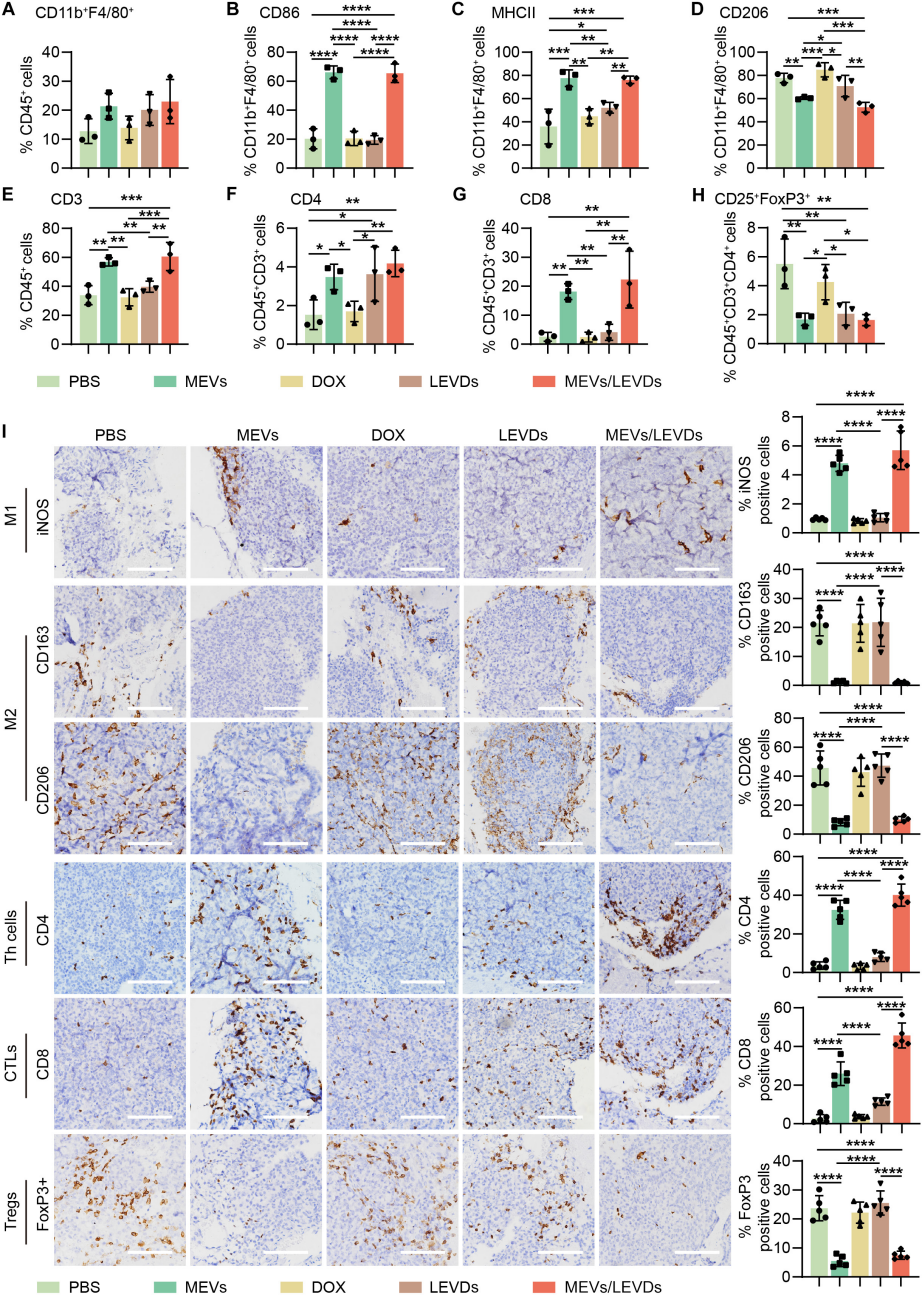
The co-EVs delivery system MEVs/LEVDs reversed the glioblastoma (GBM) immunosuppression microenvironment. (A to H) Infiltration of macrophages and T cells in GBM (*n* = 3). (I) Immunohistochemistry (IHC) images of M1 (inducible nitric oxide synthase-positive [iNOS^+^]), M2 (CD163^+^ or CD206^+^) macrophages, CD4^+^, CD8^+^, and regulatory (Tregs) (FoxP3^+^) T cells in GBM (*n* = 5). Scale bar: 100 μm. Data are represented as means ± SD. Statistical significance was determined using 1-way analysis of variance (ANOVA) followed by Bonferroni’s post hoc test, *P < 0.05; **P < 0.01; ***P < 0.001; ****P < 0.0001.

We subsequently examined immune cell infiltration in GBM tissues to elucidate the antitumor immune response of the co-EVs delivery system MEVs/LEVDs. We collected the GBM tissues from GL261-Luc-bearing C57BL/6 J mice following 5 treatment cycles to examine macrophages and T cell phenotypes. The results of flow cytometry revealed that, although there was no significant difference in total macrophage (CD11b^+^F4/80^+^) infiltration (Fig. S37A and B and Fig. 7A), both MEVs/LEVDs and MEVs significantly enhanced the proportion of M1 TAMs (CD86^+^ or MHCII^+^) and reduced the proportion of M2 TAMs (CD206^+^) (Fig. S37C to F and Fig. 7B to D). These results indicated that the MEVs/LEVDs successfully repolarized M2 TAMs to an M1 phenotype in GBM tissues. CXCL10, which is usually secreted by M1 TAMs, plays a vital role in recruiting activated T cells to tumor sites to enhance immunotherapy [42–46]. As described above, CXCL10 was significantly up-regulated in MEV-treated M2 RAW264.7 cells (Fig. 2G). Thus, we investigated whether CXCL10 expression could be augmented in the GBM tissues after treatment with MEVs/LEVDs. It showed the CXCL10 was up-regulated at both mRNA and protein levels in MEV/LEVD- and MEV-treated tumors (Fig. S38). Accordingly, compared to DOX and LEVDs, MEVs/LEVDs significantly enhanced the infiltration of total T cells (CD3^+^) (Fig. 7E), including the CD4^+^ T helper cells (Fig. 7F) and CD8^+^ cytotoxic T cells (Fig. 7G and Fig. S39), which are known to exert a cytotoxic effect in conjunction with M1 macrophages. In the tumor microenvironment, an overabundance of regulatory T cells (Tregs) may inhibit the activity of CD8^+^ T cells [47,48]. We therefore determined the infiltration of Tregs (CD45^+^CD3^+^CD4^+^CD25^+^FoxP3^+^). The results indicated that MEVs/LEVDs and MEVs, as well as LEVDs, could reduce the proportions of Tregs compared to DOX and PBS (Fig. 7H and Fig. S40). Immunological markers in GBM were also identified through IHC staining. The results confirmed increased iNOS^+^, CD4^+^, and CD8^+^ cells and decreased CD163^+^, CD206^+^, and FoxP3^+^ cells in MEV/LEVD- and MEV-treated tumors (Fig. 7I). These results revealed the co-EVs delivery system MEVs/LEVDs promoted the infiltration of cytotoxic T cells and reduced Tregs by repolarizing M2 TAMs, thereby reversing immunosuppression and enhancing immunochemotherapy efficacy against GBM.

## Discussion

EVs have emerged as pivotal mediators of intercellular communication, capable of transferring bioactive cargoes such as proteins, lipids, and nucleic acids to alter the phenotype and function of recipient cells [49]. Concurrently, their intrinsic ability to encapsulate and protect therapeutic payloads has positioned them as promising natural drug delivery vehicles [49]. In this study, we harnessed both innate functions of EVs to develop a unique codelivery system for enhanced GBM treatment. By employing 2 distinct EV populations—M0 macrophage-derived EVs (MEVs) for immunomodulation and DOX-loaded LEVDs for chemotherapy—we established a platform that enables simultaneous and independent targeting of the 2 major cellular components of the GBM microenvironment: TAMs and tumor cells. Our results demonstrate that MEVs, rich in let-7f-5p, effectively repolarize immunosuppressive M2 TAMs to an antitumor M1 phenotype by targeting the A20/NF-κB signaling pathway. Independently, LEVDs, functionalized with cRGD peptides, efficiently delivered DOX to GBM cells after traversing the BBB/BBTB. The combination of these 2 EV functionalities resulted in a significant and synergistic enhancement of anti-GBM efficacy, marked by robust tumor suppression, increased infiltration of M1 macrophages and cytotoxic T lymphocytes, and reversal of the immunosuppressive tumor microenvironment. Our study highlights the potential of leveraging the natural biological functions of diverse EV populations to design sophisticated, multipronged therapeutic strategies for complex diseases like GBM.

Despite the promising results, this study has several limitations. First, GBM is characterized by significant heterogeneity. Future studies should validate the robustness of this codelivery strategy in additional, more diverse GBM models, including patient-derived xenografts or genetically engineered mouse models that more accurately recapitulate the complexity of human GBM. Second, clinical translation of EV-based therapies faces substantial hurdles. A primary challenge is the scalable production and isolation of clinical-grade EVs. In this study, the yield of EVs from cell culture, particularly from primary cells like BMDMs, was relatively low, necessitating the use of the RAW264.7 cell line for in vivo studies. For clinical application, development of large-scale good manufacturing practice protocols for EV production, such as using bioreactors for cell culture and standardized methods for EV isolation and purification, is critical. In addition, the heterogeneity of EV populations poses challenges for quality control and batch-to-batch consistency. Although we demonstrated consistent drug loading across multiple LEVD batches, the inherent variability in EV cargo must be rigorously characterized and controlled to ensure reproducible therapeutic outcomes. Furthermore, another significant consideration is the safety profile of the co-EVs system. While our preliminary biosafety evaluation in mice showed no acute toxicity, cardiac dysfunction, or major organ damage, long-term toxicity studies are essential. Additionally, the long-term fate and potential off-target effects of EV components, especially the miRNAs carried by MEVs, remain unknown. While our study demonstrates a favorable short-term safety profile, chronic toxicity studies in larger animal models are necessary prerequisites for advancing this delivery system toward clinical trials.

Notwithstanding these challenges, the modularity of our co-EVs platform—where each EV type can be independently optimized or replaced—offers a flexible and powerful strategy. By addressing the hurdles of scalable production and long-term safety, this approach holds significant promise for developing next-generation combination immunochemotherapies for GBM and potentially other malignancies.

## Materials and Methods

### Animals and tumor models

Female C57BL/6 J mice (6 to 8 weeks old) were purchased from Guangdong Medical Laboratory Animal Center and kept under specific pathogen-free conditions with humidity 40% to 60% and 20 to 26 °C temperature. All animal experiments were approved by the Institutional Animal Care and Use Committee of Southern Medical University, Guangzhou, China (approval no. L2022096) and conducted in accordance with ARRIVE guidelines (Animal Research: Reporting of In Vivo Experiments). Orthotopic GBM-bearing mice were generated by intracranial injection of GL261-Luc or GL261-mCherry cells (5 × 10^3^ suspended in 5 μl of PBS) into the striatum as previously described [29]. Tumor growth was monitored by bioluminescence using the IVIS (IVIS Lumina II, Caliper, USA), after intraperitoneal injection of D-luciferin potassium salt (150 mg·kg^−1^). Blood was collected, and mice were euthanized with CO_2_ and sacrificed by cervical dislocation at designated time points.

### Small RNA sequencing of MEVs

Total RNA was extracted from MEVs using RNAex Pro RNA Reagent (AG21101) for small RNA (*n* = 3). RNA quality and concentration were assessed with an Agilent 2100 Analyzer and NanoDrop 2000. Small RNA sequencing and analysis were performed by Shanghai OE Biotech Co., Ltd. Briefly, miRNA sequencing libraries were constructed using the TruSeq Small RNA Sample Preparation Kit (Illumina) and sequenced on an Illumina HiSeq 2000 platform, generating 150-bp paired-end reads. Clean reads that passed the quality filter were processed to remove the adapter sequences as trimmed reads. The trimmed reads were aligned against miRBase (http://www.mirbase.org/) to identify known mature parasite miRNAs. Sequencing data has been uploaded to the Sequence Read Archive database under accession numbers SRR27230044, SRR27230045, and SRR27230046.

### In vivo antitumor experiments

For evaluation of the repolarization effect of MEVs on TAMs in vivo, 5 × 10^5^ GL261 cells were subcutaneously injected into the left flanks of C57BL/6 J mice (*n* = 3). Tumor volume was calculated using calipers according to the following formula: *L* × *W*^2^ × π/6, where *L* is the length and *W* is the width of the tumor. When tumors reached ~50 mm^3^, mice received intratumoral injections of 50 μg of MEVs or an equal volume of PBS every 3 d for a total of 3 injections. Tumors were harvested 2 d after the last injection. The IHC assay was carried out to determine the expression of iNOS, CD163, and CD206.

For anti-GBM experiments, the orthotopic GL261-Luc-bearing mice were generated and randomly divided into different treatment groups without any bias on body weight and tumor size (*n* = 6). To evaluate the anti-GBM efficacy of MEVs, GL261-Luc-bearing mice were administered with MEVs (5 μg·g^−1^) via the tail vein every 5 d for a total of 6 injections. After treatment, the fluorescence intensity of GBM was detected every 5 d using IVIS. To evaluate the anti-GBM efficacy of the co-EVs delivery system MEVs/LEVDs, GL261-Luc-bearing mice were given with MEVs (5 μg·g^−1^), DOX, LEVDs (DOX dosage: 2.5 μg·g^−1^), or different dosages of MEVs (1, 2.5, or 5 μg·g^−1^) plus LEVDs (DOX dosage: 2.5 μg·g^−1^) via tail vein every 5 d for a total of 6 injections. The fluorescent intensity of GBM was assessed by IVIS every 7 d. Day 0 was defined as the first day of treatment. PBS-treated mice served as controls. All animal procedures were approved by the Institutional Animal Care and Use Committee of Southern Medical University (approval no. L2022096), with specific approval for monitoring animals to natural death in the survival end point study despite anticipated significant weight loss exceeding standard humane end point thresholds. This approval was granted based on the scientific necessity of obtaining unbiased survival data for evaluating therapeutic efficacy for the aggressive GBM model. The body weight of all mice was detected every day until the mice died. In addition, all mice were inspected twice daily, and the survival time was recorded.

### Statistical analysis

Data were presented as means ± SD. Analyses were performed using GraphPad Prism software (version 9.1.0) and IBM SPSS 19.0 software. For comparison between only 2 groups, the unpaired Student *t* test (equal variances) or unpaired Welch’s *t* test (unequal variances) was used. For comparison between more than 2 groups, 1-way analysis of variance (ANOVA) followed by Bonferroni’s post hoc test (with equal variances) or Welch’s ANOVA (unequal variances) was employed. For survival analysis, Kaplan–Meier survival curves were plotted, and a log-rank test was performed. Differences were considered significance at *P < 0.05, **P < 0.01, ***P < 0.001, and ****P < 0.0001.

## Data Availability

The data that support the findings of this study are available on reasonable request from the corresponding authors. Sequencing data in our study has been uploaded to the Sequence Read Archive database under accession numbers SRR27230044, SRR27230045, and SRR27230046.

## References

[1] Schaff LR, Mellinghoff IK. Glioblastoma and other primary brain malignancies in adults: A review. JAMA. 2023;329(7):574–587. 36809318 10.1001/jama.2023.0023 PMC11445779

[2] Wu D, Chen Q, Chen X, Han F, Chen Z, Wang Y. The blood-brain barrier: Structure, regulation, and drug delivery. Signal Transduct Target Ther. 2023;8(1):217. 37231000 10.1038/s41392-023-01481-w PMC10212980

[3] Terstappen GC, Meyer AH, Bell RD, Zhang W. Strategies for delivering therapeutics across the blood-brain barrier. Nat Rev Drug Discov. 2021;20(5):362–383. 33649582 10.1038/s41573-021-00139-y

[4] Pandit R, Chen L, Götz J. The blood-brain barrier: Physiology and strategies for drug delivery. Adv Drug Deliv Rev. 2020;165-166:1–14. 31790711 10.1016/j.addr.2019.11.009

[5] Galstyan A, Markman JL, Shatalova ES, Chiechi A, Korman AJ, Patil R, Klymyshyn D, Tourtellotte WG, Israel LL, Braubach O, et al. Blood-brain barrier permeable nano immunoconjugates induce local immune responses for glioma therapy. Nat Commun. 2019;10(1):3850. 31462642 10.1038/s41467-019-11719-3 PMC6713723

[6] Liu J, Gao D, Hu D, Lan S, Liu Y, Zheng H, Yuan Z, Sheng Z. Delivery of biomimetic liposomes via meningeal lymphatic vessels route for targeted therapy of Parkinson’s disease. Research. 2023;6:0030. 37040500 10.34133/research.0030 PMC10076012

[7] Khan F, Pang L, Dunterman M, Lesniak MS, Heimberger AB, Chen P. Macrophages and microglia in glioblastoma: Heterogeneity, plasticity, and therapy. J Clin Invest. 2023;133(1): Article e163446. 36594466 10.1172/JCI163446 PMC9797335

[8] Chen C, Jing W, Chen Y, Wang G, Abdalla M, Gao L, Han M, Shi C, Li A, Sun P, et al. Intracavity generation of glioma stem cell-specific CAR macrophages primes locoregional immunity for postoperative glioblastoma therapy. Sci Transl Med. 2022;14(656):eabn1128. 35921473 10.1126/scitranslmed.abn1128

[9] Ni X, Wu W, Sun X, Ma J, Yu Z, He X, Cheng J, Xu P, Liu H, Shang T, et al. Interrogating glioma-M2 macrophage interactions identifies Gal-9/Tim-3 as a viable target against *PTEN*-null glioblastoma. Sci Adv. 2022;8(27):eabl5165. 35857445 10.1126/sciadv.abl5165 PMC9269888

[10] Kreatsoulas D, Bolyard C, Wu BX, Cam H, Giglio P, Li Z. Translational landscape of glioblastoma immunotherapy for physicians: Guiding clinical practice with basic scientific evidence. J Hematol Oncol. 2022;15(1):80. 35690784 10.1186/s13045-022-01298-0 PMC9188021

[11] van den Bossche WBL, Kleijn A, Teunissen CE, Voerman JSA, Teodosio C, Noske DP, van Dongen JJM, Dirven CMF, Lamfers MLM. Oncolytic virotherapy in glioblastoma patients induces a tumor macrophage phenotypic shift leading to an altered glioblastoma microenvironment. Neuro Oncol. 2018;20(11):1494–1504. 29796615 10.1093/neuonc/noy082 PMC6176807

[12] Hyun GH, Jeong D-H, Yang YY, Cho IH, Ha Y-J, Xing X, Abbott DW, Hsieh YSY, Kang YP, Cha J-H, et al. Multivalent carbohydrate nanocomposites for tumor microenvironment remodeling to enhance antitumor immunity. ACS Nano. 2023;17(12):11567–11582. 37306074 10.1021/acsnano.3c01645 PMC10311601

[13] Gao X, Li S, Ding F, Liu X, Wu Y, Li J, Feng J, Zhu X, Zhang C. A virus-mimicking nucleic acid nanogel reprograms microglia and macrophages for glioblastoma therapy. Adv Mater. 2021;33(9): Article e2006116. 33501743 10.1002/adma.202006116

[14] Nie W, Wu G, Zhang J, Huang LL, Ding J, Jiang A, Zhang Y, Liu Y, Li J, Pu K, et al. Responsive exosome nano-bioconjugates for synergistic cancer therapy. Angew Chem Int Ed Engl. 2020;59(5):2018–2022. 31746532 10.1002/anie.201912524

[15] Parayath NN, Parikh A, Amiji MM. Repolarization of tumor-associated macrophages in a genetically engineered nonsmall cell lung cancer model by intraperitoneal administration of hyaluronic acid-based nanoparticles encapsulating microRNA-125b. Nano Lett. 2018;18(6):3571–3579. 29722542 10.1021/acs.nanolett.8b00689

[16] Kulkarni A, Chandrasekar V, Natarajan SK, Ramesh A, Pandey P, Nirgud J, Bhatnagar H, Ashok D, Ajay AK, Sengupta S. A designer self-assembled supramolecule amplifies macrophage immune responses against aggressive cancer. Nat Biomed Eng. 2018;2(8):589–599. 30956894 10.1038/s41551-018-0254-6 PMC6450396

[17] Zhang T, Lip H, He C, Cai P, Wang Z, Henderson JT, Rauth AM, Wu XY. Multitargeted nanoparticles deliver synergistic drugs across the blood–brain barrier to brain metastases of triple negative breast cancer cells and tumor-associated macrophages. Adv Healthc Mater. 2019;8(18): Article e1900543. 31348614 10.1002/adhm.201900543

[18] Wang J, Shen S, Li J, Cao Z, Yang X. Precise depletion of tumor seed and growing soil with shrinkable nanocarrier for potentiated cancer chemoimmunotherapy. ACS Nano. 2021;15(3):4636–4646. 33651592 10.1021/acsnano.0c08996

[19] Wan D, Yang Y, Liu Y, Cun X, Li M, Xu S, Zhao W, Xiang Y, Qiu Y, Yu Q, et al. Sequential depletion of myeloid-derived suppressor cells and tumor cells with a dual-pH-sensitive conjugated micelle system for cancer chemoimmunotherapy. J Control Release. 2020;317:43–56. 31758970 10.1016/j.jconrel.2019.11.011

[20] Qiu N, Wang G, Wang J, Zhou Q, Guo M, Wang Y, Hu X, Zhou H, Bai R, You M, et al. Tumor-associated macrophage and tumor-cell dually transfecting polyplexes for efficient interleukin-12 cancer gene therapy. Adv Mater. 2021;33(2): Article e2006189. 33270281 10.1002/adma.202006189

[21] Deng C, Zhang Q, Jia M, Zhao J, Sun X, Gong T, Zhang Z. Tumors and their microenvironment dual-targeting chemotherapy with local immune adjuvant therapy for effective antitumor immunity against breast cancer. Adv Sci. 2019;6(6):1801868. 10.1002/advs.201801868 PMC6425447 30937266

[22] Song Y, Tang C, Yin C. Combination antitumor immunotherapy with VEGF and PIGF siRNA via systemic delivery of multi-functionalized nanoparticles to tumor-associated macrophages and breast cancer cells. Biomaterials. 2018;185:117–132. 30241030 10.1016/j.biomaterials.2018.09.017

[23] Li T, Chen D, Liu H, Tao Y, He X, Zang S, Li J, Zhang L, Li M, Liu J, et al. Spatially targeting and regulating tumor-associated macrophages using a raspberry-like micellar system sensitizes pancreatic cancer chemoimmunotherapy. Nanoscale. 2022;14(36):13098–13112. 35972382 10.1039/d2nr03053e

[24] Cheng L, Zhang P, Liu Y, Liu Z, Tang J, Xu L, Liu J. Multifunctional hybrid exosomes enhanced cancer chemo-immunotherapy by activating the STING pathway. Biomaterials. 2023;301: Article 122259. 37531777 10.1016/j.biomaterials.2023.122259

[25] Wilhelm S, Tavares AJ, Dai Q, Ohta S, Audet J, Dvorak HF, Chan WCW. Analysis of nanoparticle delivery to tumours. Nat Rev Mater. 2016;1(5):16014.

[26] Zelepukin IV, Shevchenko KG, Deyev SM. Rediscovery of mononuclear phagocyte system blockade for nanoparticle drug delivery. Nat Commun. 2024;15(1):4366. 38777821 10.1038/s41467-024-48838-5 PMC11111695

[27] Zitvogel L, Apetoh L, Ghiringhelli F, Kroemer G. Immunological aspects of cancer chemotherapy. Nat Rev Immunol. 2008;8(1):59–73. 18097448 10.1038/nri2216

[28] Galluzzi L, Buqué A, Kepp O, Zitvogel L, Kroemer G. Immunological effects of conventional chemotherapy and targeted anticancer agents. Cancer Cell. 2015;28(6):690–714. 26678337 10.1016/j.ccell.2015.10.012

[29] Niu W, Xiao Q, Wang X, Zhu J, Li J, Liang X, Peng Y, Wu C, Lu R, Pan Y, et al. A biomimetic drug delivery system by integrating grapefruit extracellular vesicles and doxorubicin-loaded heparin-based nanoparticles for glioma therapy. Nano Lett. 2021;21(3):1484–1492. 33475372 10.1021/acs.nanolett.0c04753

[30] Wang Z, Liu Z, Wang S, Bing X, Ji X, He D, Han M, Wei Y, Wang C, Xia Q, et al. Implantation of hydrogel-liposome nanoplatform inhibits glioblastoma relapse by inducing ferroptosis. Asian J Pharm Sci. 2023;18(3): Article 100800. 37274924 10.1016/j.ajps.2023.100800 PMC10232663

[31] Schulz-Siegmund M, Aigner A. Nucleic acid delivery with extracellular vesicles. Adv Drug Deliv Rev. 2021;173:89–111. 33746014 10.1016/j.addr.2021.03.005

[32] O’Brien K, Breyne K, Ughetto S, Laurent LC, Breakefield XO. RNA delivery by extracellular vesicles in mammalian cells and its applications. Nat Rev Mol Cell Biol. 2020;21(10):585–606. 32457507 10.1038/s41580-020-0251-y PMC7249041

[33] Liu S, Chen L, Guo M, Li Y, Liu Q, Cheng Y. Targeted delivery of engineered RVG-BDNF-exosomes: A novel neurobiological approach for ameliorating depression and regulating neurogenesis. Research. 2024;7:0402. 41221079 10.34133/research.0402 PMC12599878

[34] Chen Y, Tang S, Cai F, Wan Y. Strategies for small extracellular vesicle-based cancer immunotherapy. Research. 2024;7:0421. 39040921 10.34133/research.0421 PMC11260559

[35] Isaac R, Reis FCG, Ying W, Olefsky JM. Exosomes as mediators of intercellular crosstalk in metabolism. Cell Metab. 2021;33(9):1744–1762. 34496230 10.1016/j.cmet.2021.08.006 PMC8428804

[36] Ambros V. The functions of animal microRNAs. Nature. 2004;431(7006):350–355. 15372042 10.1038/nature02871

[37] Thomou T, Mori MA, Dreyfuss JM, Konishi M, Sakaguchi M, Wolfrum C, Rao TN, Winnay JN, Garcia-Martin R, Grinspoon SK, et al. Adipose-derived circulating miRNAs regulate gene expression in other tissues. Nature. 2017;542(7642):450–455. 28199304 10.1038/nature21365 PMC5330251

[38] McGeary SE, Lin KS, Shi CY, Pham TM, Bisaria N, Kelley GM, Bartel DP. The biochemical basis of microRNA targeting efficacy. Science. 2019;366(6472): Article eaav1741. 31806698 10.1126/science.aav1741 PMC7051167

[39] Wertz IE, O’Rourke KM, Zhou H, Eby M, Aravind L, Seshagiri S, Wu P, Wiesmann C, Baker R, Boone DL, et al. De-ubiquitination and ubiquitin ligase domains of A20 downregulate NF-κB signalling. Nature. 2004;430(7000):694–699. 15258597 10.1038/nature02794

[40] Lee EG, Boone DL, Chai S, Libby SL, Chien M, Lodolce JP, Ma A. Failure to regulate TNF-induced NF-κB and cell death responses in A20-deficient mice. Science. 2000;289(5488):2350–2354. 11009421 10.1126/science.289.5488.2350 PMC3582399

[41] Sharifiaghdam M, Shaabani E, Faridi-Majidi R, De Smedt SC, Braeckmans K, Fraire JC. Macrophages as a therapeutic target to promote diabetic wound healing. Mol Ther. 2022;30(9):2891–2908. 35918892 10.1016/j.ymthe.2022.07.016 PMC9482022

[42] Duan Z, Luo Y. Targeting macrophages in cancer immunotherapy. Signal Transduct Target Ther. 2021;6(1):127. 33767177 10.1038/s41392-021-00506-6 PMC7994399

[43] Hoch T, Schulz D, Eling N, Gómez JM, Levesque MP, Bodenmiller B. Multiplexed imaging mass cytometry of the chemokine milieus in melanoma characterizes features of the response to immunotherapy. Sci Immunol. 2022;7(70):eabk1692. 35363540 10.1126/sciimmunol.abk1692

[44] House IG, Savas P, Lai J, Chen AXY, Oliver AJ, Teo ZL, Todd KL, Henderson MA, Giuffrida L, Petley EV, et al. Macrophage-derived CXCL9 and CXCL10 are required for antitumor immune responses following immune checkpoint blockade. Clin Cancer Res. 2020;26(2):487–504. 31636098 10.1158/1078-0432.CCR-19-1868

[45] Zhang J, Chen C, Li A, Jing W, Sun P, Huang X, Liu Y, Zhang S, Du W, Zhang R, et al. Immunostimulant hydrogel for the inhibition of malignant glioma relapse post-resection. Nat Nanotechnol. 2021;16(5):538–548. 33526838 10.1038/s41565-020-00843-7

[46] Xu X, Zhang Z, Du J, Xue Y, Chen X, Zhang J, Yang X, Chang D, Xie J, Ju S. Recruiting T-cells toward the brain for enhanced glioblastoma immunotherapeutic efficacy by co-delivery of cytokines and immune checkpoint antibodies with macrophage-membrane-camouflaged nanovesicles. Adv Mater. 2023;35(25): Article e2209785. 37101060 10.1002/adma.202209785

[47] Maeda Y, Nishikawa H, Sugiyama D, Ha D, Hamaguchi M, Saito T, Nishioka M, Wing JB, Adeegbe D, Katayama I, et al. Detection of self-reactive CD8^+^ T cells with an anergic phenotype in healthy individuals. Science. 2014;346(6216):1536–1540. 25525252 10.1126/science.aaa1292

[48] Chen M-L, Pittet MJ, Gorelik L, Flavell RA, Weissleder R, von Boehmer H, Khazaie K. Regulatory T cells suppress tumor-specific CD8 T cell cytotoxicity through TGF-β signals in vivo. Proc Natl Acad Sci USA. 2005;102(2):419–424. 15623559 10.1073/pnas.0408197102 PMC544311

[49] Kumar MA, Baba SK, Sadida HQ, Marzooqi SA, Jerobin J, Altemani FH, Algehainy N, Alanazi MA, Abou-Samra A-B, Kumar R, et al. Extracellular vesicles as tools and targets in therapy for diseases. Signal Transduct Target Ther. 2024;9(1):27. 38311623 10.1038/s41392-024-01735-1 PMC10838959

