# Supplementary material for: Co-Extracellular Vesicles Delivery System Enhances Immunochemotherapy for Glioblastoma
Source: Research (Wash D C). 2026 Mar 31;9:1219. doi: 10.34133/research.1219 (PMC13036365; doi:10.34133/research.1219)
Supplement: Supplementary 1 — Figs. S1 to S40 Tables S1 to S6 [file research.1219.f1.docx]

Supplementary Materials

**Co-Extracellular Vesicles Delivery System Enhances Immunochemotherapy for Glioblastoma**

Wenbo Niu^1,†^, Rui Guan^1,†^, Le Sun^1^, Mingqing Wang^2^, Xuejiao Wang^3^, Biao Zhang^3^, Xiangrong Hao^3^, Qun Wu^3^, Zhongman Cheng^3^, Jiahui Wei^3^, Ying Wang^3,*^, Jian Zhang^1,*^, and Jun-Bing Fan^3,*^

^1^Department of Oncology, Zhujiang Hospital, Southern Medical University, Guangzhou, 510282, P. R. China.

^2^School of Traditional Chinese Medicine, Southern Medical University, Guangzhou 510515, P. R. China

^3^Cancer Research Institute, School of Basic Medical Sciences, Southern Medical University, Guangzhou 510515, P. R. China.

^*^Address correspondence to: ningmengquan@gmail.com (Y.W.);

^*^Address correspondence to: zhangjian@i.smu.edu.cn (J.Z.);

^*^Address correspondence to: fjb2012@mail.ipc.ac.cn (J.F.)

^†^These authors contributed equally to this work.

**Materials and reagents**

Roswell Park Memorial Institute medium (RPMI 1640) and Dulbecco’s modified Eagle’s medium (DMEM) were obtained from Gibco (New York, USA). Fetal bovine serum (FBS) was obtained from ExCell Bio. (Suzhou, China). Lentivirus carrying a reporter gene encoding fireﬂy luciferase or mCherry was purchased from Shanghai Genechem Co., Ltd. (Shanghai, China). D-luciferin potassium salt was purchased from PerkinElmer Inc. (Massachusetts, USA). Recombinant mouse CSF-1, recombinant mouse IFN-γ, recombinant mouse IL-4, and recombinant human IL-13 were purchased from PeproTech, Inc. (New Jersey, USA). Phorbol 12-myristate 13-acetate (PMA), PKH67 fluorescent dye, lipopolysaccharide (LPS), 1-Ethyl-3-(3-dimethylaminopropyl)carbodiimide (EDC), N-hydroxysuccinimide (NHS) and 4′,6′-diamidino-2-phenylindole (DAPI) were purchased from Sigma-Aldrich (St. Louis, USA). Sulfo Cyanine7 NHS ester was purchased from Xi'an Ruixi Biological Technology Co., Ltd. (Xi'an, China). RNAex Pro RNA Reagent, Evo M-MLV RT Kit with gDNA Clean for qPCR II and SYBR® Green Pro Taq HS Premix II were purchased from Accurate Biology (Hunan, China). Rfect siRNA/miRNA Transfection Reagent was purchased from Baidai biotechnology (Changzhou, China). MiRNA mimics and let-7f-5p inhibitor were synthesized by Tsingke Biotechnology Co., Ltd (Beijing, China). The Dual-Luciferase Reporter Assay Kit was purchased from Promega Corporation (Madison, USA). 6.5 mm Transwell^®^ inserts with 0.4 µm Pore Polyester Membrane were purchased from Corning (New York, USA). The TUNEL Assay Kit was purchased from Elabscience Biotechnology Inc. (Wuhan, China). 5(6)-Carboxyfluorescein diacetate, succinimidyl ester (CFSE) and PECAM-1 Antibody were purchased from Santa Cruz Biotechnology (Dallas, USA). Doxorubicin hydrochloride (DOX) (A603456) was purchased from Sangon Biotech (Shanghai, China). Type-IV Collagenase and DNase I were purchased from Thermo Fisher Scientific Inc. (Waltham, USA). ‎Cell Counting Kit-8 (CCK8) was purchased from Dojindo Molecular Technologies, Inc. (Maryland, USA). Antibodies against TSG101, Alix, CD9, iNOS, NF-κB p65, ZO-1, Claudin-1, αV, β3, αVβ3, CD163, CD206, as well as Goat anti-rabbit IgG (Alexa Fluor 488), Goat anti-mouse IgG (Alexa Fluor 488) and Goat anti-rabbit IgG (Alexa Fluor 594) secondary antibodies were purchased from Abcam company (Cambridge, UK). Antibodies against Ki67 and CD34 were purchased from MXB Biotechnologies (Xiamen, China). Antibody against CXCL10 was purchased from Proteintech Group, Inc. (Wuhan, China). Rat tail collagen I, Mouse Fc receptor blocker, Fixable Viability Stain 780, Fixation/Permeabilization Solution Kit, and flow cytometry antibodies against CD45, MHCII, CD86, CD25 and FoxP3 were purchased from BD Biosciences (California, USA). Flow cytometry antibodies against F4/80, CD80, CD206, CD3ε, CD4 and CD8a were purchased from Biolegend Inc. (San Diego, USA). Flow cytometry antibody against CD11b was purchased from eBioscience, Inc. (San Diego, USA).

**Cell lines and culture**

Human GBM cell lines LN229 and U87, human monocyte cell line THP-1, mouse macrophages cell line RAW264.7, and mouse endothelial cell line bEnd.3 were purchased from the American Type Culture Collection (ATCC). Mouse GBM cell line GL261 and Human GBM cell line U251 were kindly provided by the Guangdong Provincial Key Laboratory of Brain Function Repair and Regeneration, Department of Neurosurgery, Zhujiang Hospital, Southern Medical University. GL261-Luc and GL261-mCherry cell lines were generated by transducing GL261 cells with lentivirus carrying a reporter gene encoding firefly luciferase or mCherry, respectively, according to the manufacturer's instructions. Transduced GL261 cells were then selected with 3 µg mL^-1^ puromycin for 7 days to establish stable luciferase- or mCherry-expressing cell lines. THP-1 cells were maintained in RPMI 1640 medium while other cell lines were cultured in DMEM supplemented with 10% fetal bovine serum (FBS). All cell lines used tested negative for mycoplasma contamination. Cells were incubated at 37°C under a humidified atmosphere containing 5% CO_2_.

**Bone marrow-derived macrophages (BMDMs) isolation and polarization**

Primary BMDMs were generated from 6-week-old female wild-type C57BL/6J mice. Bone marrow cells were flushed from femurs and tibiae, filtered through a 40 μm cell strainer, and subjected to red blood cell lysis using ACK lysing buffer (Thermo Fisher Scientific) on ice for 5 min. Cells were centrifuged at 300 g for 10 min to remove the lysed red blood cells, then cultured in DMEM supplemented with 10% FBS and recombinant mouse CSF-1 (20 ng mL^-1^, PeproTech) for 7 days to allow differentiation into M0 macrophages. The purity of BMDMs was assessed by flow cytometry using double staining with CD11b and F4/80 antibodies. For M1 polarization, M0 BMDMs were incubated with LPS (100 ng mL^-1^, Sigma-Aldrich) plus recombinant mouse IFN-γ (20 ng mL^-1^, PeproTech) for 48 h. For M2 polarization, M0 BMDMs were incubated with recombinant mouse IL-4 (20 ng mL^-1^, PeproTech) for 48 h.

**Macrophages cell line polarization**

Unstimulated RAW264.7 cells were considered as M0 phenotype. For M1 polarization, RAW264.7 cells were treated with LPS (100 ng mL^-1^, Sigma-Aldrich) plus recombinant mouse IFN-γ (20 ng mL^-1^, PeproTech) for 24 h. For M2 polarization, RAW264.7 cells were treated with recombinant mouse IL-4 (20 ng mL^-1^, PeproTech) for 24 h.

THP-1 monocytes were differentiated into M0 macrophages by incubation with PMA (200 ng mL^-1^, Sigma) for 24 h, followed by further incubation in RPMI medium for additional 24 h. For M1 polarization, M0 THP-1 macrophages were cultured in RPMI medium supplemented with LPS (100 ng mL^-1^, Sigma) and recombinant human IFN-γ (20 ng mL^-1^, Sigma) for 24 h; For M2 polarization, M0 THP-1 macrophages were cultured in RPMI medium supplemented with recombinant human IL-4 (20 ng mL^-1^, PeproTech) and recombinant human IL-13 (20 ng mL^-1^, PeproTech) for 72 h.

**Extracellular vesicles (EVs) isolation, characterization and labelling**

FBS was ultracentrifuged at 120,000 g for 18 h at 4°C to deplete EVs. For EVs isolation, M0 macrophages were cultured in medium supplemented with 10% EVs-depleted FBS for 48 h. EVs from the conditioned medium (CM) of M0 macrophages were isolated by differential ultracentrifugation. Briefly, the CM was sequentially centrifuged at 300 g for 10 min at 4 °C, 2,000 g for 10 min, and 10,000 g for 20 min to remove cells and debris.

The supernatant was then filtered through a 0.45 μm filter (Millipore). EVs were pelleted by two rounds of ultracentrifugation at 120,000 g for 120 min at 4°C using a Beckman ultracentrifuge. The isolated EVs were resuspended in PBS and stored at -80°C. Protein concentration of EVs was determined using a BCA protein assay kit. For functional experiments, EVs must be used within 2 weeks after isolation.

The size distribution of EVs was measured by dynamic light scattering (DLS) using a Zetasizer Nano-Zs (Malvern Instruments, UK). Morphology of EVs was observed by TEM (Hitachi HC-1, 80 kV) after negative staining with phosphotungstic acid. EVs were characterized by western blotting for the expression of EV-specific markers TSG101, Alix, and CD9.

To monitor EVs internalization and trafficking, M0 RAW264.7-derived EVs (MEVs) were labeled with PKH67 fluorescent dye (Sigma-Aldrich) according to the manufacturer's instructions. For biodistribution studies, MEVs were labeled with Cy7 fluorescent dye. Briefly, MEVs (100 µg in 2 mL PBS) were mixed with EDC (6 mg) and NHS (6 mg), followed by addition of 100 µg Cy7-NHS (R-H-7109, Xi'an Ruixi Biological Technology). The mixture was incubated overnight at room temperature. The resulting product was washed twice by ultracentrifugation at 120,000 g for 120 min at 4°C in PBS and re-dispersed in 250 μL PBS.

**In vitro macrophages repolarization assays**

RAW264.7 cells, THP-1 cells and BMDMs were first polarized to M1 or M2 phenotypes as described above. M2 macrophages were then cultured in fresh complete medium for 12 h, followed by incubation with various concentrations of M0 macrophages-derived EVs for an additional 24 h. After treatment, cell morphology was observed by a phase-contrast microscope. Cells were then washed three times and subjected to qRT-PCR, immunofluorescence, western blotting and flow cytometry assays to evaluate M1 and M2 macrophages markers. In some experiments, induced M1 macrophages were used as a positive control.

**RNA extraction and quantitative reverse transcription polymerase chain reaction (qRT-PCR)**

Total RNA was extracted from macrophages or primary tumors using RNAex Pro RNA reagent (Accurate Biology, AG21102) according to the manufacturer's instructions. Reverse transcription was performed using Evo M-MLV RT Kit with gDNA Clean for qPCR II (Accurate Biology, AG11711). qRT-PCR was carried out on an ABI7900HT apparatus (Applied Biosystems) using 2×SYBR^®^ Green Pro Taq HS Premix II (Accurate Biology, AG11702). Relative quantification was performed using the 2^-ΔΔCT^ method, with GAPDH expression for normalization. The primers for qRT-PCR are listed in Table S4.

**Western blotting**

Macrophages and EVs were washed three times and lysed in RIPA lysis buffer supplemented with protease and phosphatase inhibitors. Protein extracts were separated by 10% SDS-PAGE and transferred onto polyvinylidene difluoride (PVDF) membranes. Membranes were blocked with 5% BSA in TBST buffer for 1 h at room temperature. Primary antibodies were diluted in blocking solution and incubated with membranes overnight at 4°C. After washing, membranes were incubated with horseradish peroxidase (HRP)-conjugated secondary antibodies for 1 h at room temperature. Signals were detected using enhanced chemiluminescence (Millipore, USA) and imaged with a Bio-Rad ChemiDoc™ MP Imaging System (Bio-Rad, USA). Primary antibodies are listed in Table S5.

**Immunofluorescence staining**

Cells were washed and fixed with 4% paraformaldehyde (PFA) for 10 min. Then the cells were blocked with 5% goat serum and permeabilized with 0.25% Triton X-100 in PBS for 10 min. Cells were incubated with primary antibodies (iNOS or NF-κB p65) overnight at 4°C, followed by incubation with Alexa Fluor 488-conjugated goat anti-rabbit secondary antibodies, and nuclear were counterstained with DAPI (Sigma-Aldrich). Fluorescence was imaged with laser scanning confocal microscopy (LSCM).

**Flow cytometry assay to evaluate M2 macrophages repolarization**

RAW264.7 cells were seeded in a 6-well plates (1×10^5^ cells/well) and polarized to an M2 phenotype as described above. M2 RAW264.7 cells were then treated with MEVs (10 μg mL^-1^) or an equivalent volume of PBS as a control. After 24 h, the medium was removed, and cells were collected following washing with PBS. Cells were stained with antibodies against CD80, CD86, MHC II, and CD206 for 30 min at 4°C in the dark, washed three times with PBS, and analyzed using a Fortessa flow cytometer (BD Biosciences) and FlowJo software (BD Biosciences). Antibodies used are listed in Table S6.

**MEVs internalization**

MEVs were labeled with PKH67 as described above. M0 RAW264.7 cells were seeded on slides in a 6-well plate (1×10^5^ cells/well) and cultured for 24 h. Cells were then incubated with PKH67-labeled MEVs (10 μg mL^-1^) for 6 h, washed twice with PBS, fixed with 4% PFA, and stained with DAPI. Cellular internalization was visualized by LSCM.

**miRNA mimic transfection**

miRNA mimic or corresponding negative control mimic (NC mimic) was chemically synthesized by Tsingke Biotechnology (Beijing, China). Transient transfection of miRNA mimic into M2 RAW264.7 cells was performed using Rfect siRNA/miRNA Transfection Reagent (Cat#:11012, Baidai biotechnology, Changzhou, China) according to the manufacturer's instructions. Briefly, M2 RAW264.7 cells were seeded in 12-well plates (1 × 10^5^ cells/well). After 12 h, cells were gently washed three times with PBS and cultured in Opti-MEM I Reduced Serum Medium (Cat#: 31985070, Thermo Fisher Scientific, USA). Cells were transfected with 100 nM miRNA mimic, NC mimic, or 200 nm let-7f-5p inhibitor and incubated for 24 h prior to harvesting for subsequent experiments. The sequences of miRNA mimics were listed in Table S1.

**Fluorescent labeling and tracing of EVs-containing let-7f-5p**

M0 RAW264.7 cells were transfected with Cy5-labeled let-7f-5p mimic. After 48 h, MEVs were isolated from the supernatant as described above. The purified MEVs were then added to M2 RAW264.7 cells at a concentration of 10 μg mL^-1^ for 24 h. Cells were washed twice with PBS, stained with DAPI, and examined for Cy5 fluorescence by LSCM.

**Luciferase reporter assay**

Let-7f-5p target gene prediction was conducted with TargetScan Mouse 8.0 (https://www.targetscan.org/vert_80/). To validate that A20 as a direct target of let-7f-5p, luciferase reporter assays were performed using the 3’ untranslated regions (UTR) of A20 containing either the wild-type (WT) or mutated let-7f-5p binding site, cloned downstream of the luciferase gene in the pmirGLO plasmid. Reporter plasmids were co-transfected with let-7f-5p mimic or NC mimic into M2 RAW264.7 cells. After 24 h, cell lysates were harvested, and firefly and Renilla luciferase activities were measured using the Dual-Luciferase Reporter Assay Kit (Promega, USA) according to the manufacturer's protocol.

**In vitro macrophages and GBM cell co-culture assay**

Non-contact co-culture of GL261 and RAW264.7 cells was conducted using a transwell system. M0 RAW264.7 cells were seeded in plates (3 10^5^/well) and treated with IL-4 (20 ng mL^-1^) for 24 h to induce M2 polarization. Then the medium was then removed, cells were washed twice with PBS, and incubated with fresh medium containing PBS (negative control) or different concentrations of MEVs for another 24 h. Subsequently, the treated-M2 RAW264.7 cells (5 × 10^4^ cells/well) were seeded into the upper compartment, while GL261 cells (1 × 10^5^ cells/well) were seeded into the lower compartment of the transwell system (pore size: 0.4 μm). After 36 h of co-culture, GL261 cell proliferation was determined by CCK8 assay. GL261 cells cultured alone were considered as the control group.

For conditioned medium (CM) experiments, M2 RAW264.7 cells were treated with PBS or various concentrations of MEVs for 24 h. The medium was then replaced with fresh DMEM containing 10% FBS and cells were cultured for an additional 24 h before CM collection. GL261 cells were seeded in plates; after 4 h, the medium was replaced with CM from treated-M2 RAW264.7 cells. The proliferation of GL261 cells was determined by the CCK8 assay. The apoptosis of GL261 cells after incubation for 36 h was detected by TUNEL assay (Cat#: E-CK-A334, Elabscience Biotechnology, China) according to the manufacturer's instructions. For colony formation assay, GL261 cells (200 cells/well) were seeded in 6-well plates and cultured with CM from treated M2 RAW264.7 cells for 10 days. Cells were then fixed with methanol for 10 min and stained with 0.1% crystal violet for 10 min. After washing with PBS, colonies were imaged, and colonies containing >50 cells were counted. Each group was plated in triplicate, and experiments were performed three times. GL261 cells cultured with CM from M0 RAW264.7 cells served as the control group.

For direct contact co-culture assay, M0 RAW264.7 cells were first polarized to M2 phenotype. M2 RAW264.7 cells were then labeled with CFSE Dye (Santa Cruz Biotechnology, USA) according to the manufacturer’s protocol and seeded into the 24-well plates (1.5 × 10^5^ cells/well). After 4 h, GL261-mCherry cells (3 × 10^4^ cells/well) were added to the wells. LPS (100 ng mL^-1^) + IFN-γ (20 ng mL^-1^), different concentrations of MEVs, or an equivalent volume of PBS were added to the culture medium (time point: 0 h). Live-cell imaging was performed using a fluorescence microscope (Nikon, Japan) for 48 hours. Cell numbers at 0 h and 48 h were quantified by counting cells in five fields of view, and the relative cell number at 48 h compared to 0 h was calculated.

**In vitro phagocytosis assay**

M0 RAW264.7 cells were polarized to an M2 phenotype and treated with MEVs (10 μg mL^-1^) or PBS for 24 h, then the treated-M2 RAW264.7 cells were harvested and seeded into a 24-well plates (110^5^ cells/well). GL261 cells (3 10^5^) were labeled with CFSE and then added to the treated-M2 RAW264.7 cells, followed by co-cultured for 12 h. Cells were harvested, stained with APC-CD11b primary antibodies for 30 min at 4°C in the dark, washed three times with PBS, and analyzed by flow cytometry using a Fortessa flow cytometer (BD Biosciences). The percentage of APC^+^CFSE^+^ double-positive cells was quantified using FlowJo software (BD Biosciences).

**Preparation and characterization of LEVDs**

The EVs isolation from lemons and the doxorubicin-loaded heparin-based nanoparticles (DNs) were prepared according to our previous study [29]. To construct LEVDs, 100 µg of lemon-derived EVs, 1000 µg of DNs, 6 mg EDC, and 6 mg NHS were dissolved into 2 mL deionized water and allowed to react for 12 h at room temperature. The resulting product was washed with PBS, pelleted by two rounds of ultracentrifugation at 120,000 g for 1 h each, and re-dispersed in 1 mL PBS for further characterization. To evaluate DOX loading performance, three batches of LEVDs were prepared. The amount of DOX loaded was measured using UV-vis spectrometry, and the mass of lyophilized LEVDs was determined. The DOX loading efficiency was calculated by: (Weight of DOX in LEVDs/Weight of DOX initially added) × 100%. DOX loading capacity was calculated by: (Weight of DOX in LEVDs/Weight of LEVDs-Weight of DOX in LEVDs) × 100%. The loading amount of DOX and cRGD peptide per µg of LEVs was determined as described in our previous study [29].

**Drug release**

In vitro DOX release from LEVDs was performed in 0.1 M PBS (pH 7.4) and 0.1 M acetate buffer (pH 5.0), respectively. Briefly, LEVDs dissolved in PBS or acetate buffer was placed into a dialysis membrane (MWCO 3500), which was then immersed in 100 mL of the corresponding buffer and incubated at 37°C with gentle shaking. At predetermined time points, aliquots of the dialysate were withdrawn and replaced with an equal volume of fresh buffer. DOX concentration was determined by UV-vis spectrometry at 480 nm using a standard curve.

**Stability of LEVDs**

LEVs and LEVDs were incubated in PBS containing 50% FBS at 37°C. At indicated time points, the size of LEVs and LEVDs was measured by DLS. Measurements were performed in triplicate, and the average values were recorded.

**In vitro BBB model**

An in vitro BBB model was established as described previously [29]. Briefly, the transwell membranes were first coated with rat tail collagen I (BD Biosciences, USA) according to the manufacturer’s instructions. Then bEnd.3 cells (2×10^4^ cells/well) were seeded into the upper compartment in 300 μL DMEM supplemented with 10% FBS. Transendothelial electric resistance (TEER) value of bEnd.3 monolayers were measured using a Millicell ERS-2 (Millipore, USA) after 3 days of culture. When TEER values exceeded 200 Ω·cm², 5×10^4^ GL261 cells in 600 μL DMEM with 10% FBS were seeded onto the glass coverslips placed in lower compartment of transwell system and co-cultured with bEnd.3 cells for an additional day. Prior to transcytosis assays, the expression of tight junction proteins (Claudin-1 and ZO-1) and cell-cell adhesion protein PECAM-1 in endothelial cells was verified by immunofluorescence assay.

To assess the BBB penetration capability of MEVs, LEVDs, or the co-EVs delivery system MEVs/LEVDs, PKH67-labeled MEVs (10 μg mL^-1^), LEVDs (DOX concentration: 10 μg mL^-1^), or a combination of both were added to the upper compartment and incubated for 8 h. Subsequently, the transwell membrane with bEnd.3 cells and the glass slide with GL261 cells were fixed with 4% formaldehyde for 10 min, and stained with DAPI at room temperature for 10 min. Uptake of PKH67-MEVs and/or LEVDs in bEnd.3 and GL261 cells was visualized by LSCM.

**Detection of intercellular transport of MEVs**

Transcytosis of MEVs across cerebral microvascular endothelial cells to GBM cells was assessed using a co-culture system. bEnd.3 cells (1×10^5^) were seeded on slides placed in 6-well plates and incubated with PKH67-MEVs (10 μg mL^-1^) for 24 h. The medium was then removed, and cells were washed three times with PBS. Another slide seeded with GL261 cells (2×10^5^) was placed into the same well, and fresh medium (1 mL) was added. After 9 h of co-culture, the medium was removed, cells were washed with PBS, and the PKH67 signal in GL261 cells was examined by fluorescence microscopy.

**Biodistribution and accumulation of MEVs in GBM-bearing mice**

Orthotopic GBM-bearing mice were generated by intracranial injection of GL261-mCherry cells into C57BL/6J mice as described above. MEVs were labeled with PKH67 (PKH67-MEVs) as described. Mice were intravenously injected with a single dose of PKH67-MEVs (5 μg g^-1^ body weight). At 12 h post-injection, mice were sacrificed and transcardially perfused with PBS. Subsequently, the brain and major organs (including the heart, liver, spleen, lungs, kidneys, stomach and intestines) were dissected and frozen-sectioned into 10-μm-thick slices. Sections were fixed in cold acetone for 10 min, washed twice with PBS, and stained with DAPI. Fluorescence was visualized by LSCM with excitation at 488 nm for PKH67-MEVs and 552 nm for GL261-mCherry.

GL261-Luc-bearing mice were also generated. MEVs were labeled with Cy7 (Cy7-MEVs) as described. Mice received a single tail vein injection of Cy7-MEVs (5 μg g^-1^) or an equivalent amount of free Cy7 dye as control. At 3 h and 24 h post-injection, mice were administered D-luciferin potassium salt (150 mg kg^-1^, PerkinElmer, USA), sacrificed, and their brains and major organs were excised. Cy7 fluorescence and luciferase signals in tumor-bearing brains and organs were visualized using an IVIS imaging system.

**Integrin αV and β3 expression in GBM and cerebral microvascular endothelial cells detected by flow cytometry**

GBM cells (GL261, LN229, U251 and U87) or cerebral microvascular endothelial cells (bEnd.3) (1.0×10^5^ cells) were suspended in 100 μL PBS, and incubated with primary antibodies (at a 1:100 dilution ratio) for 30 min on ice. Cells were then washed three times with PBS and incubated with secondary antibodies for another 30 min on ice in the dark. After three washes with PBS, αVβ3 expression was analyzed by flow cytometry using a Fortessa flow cytometer (BD Biosciences). Antibodies are listed in Table S6.

**BBB/BBTB penetration ability of LEVDs**

GL261-Luc-bearing mice and healthy tumor-free mice were intravenously injected with a single dose of DOX, DNs, or LEVDs (DOX dose: 2.5 μg g^-1^). At 12 h post-injection, DOX fluorescence in brain and GBM tissues was examined by LSCM as described above.

**Affinity of MEVs and LEVDs to macrophages and GBM cells**

To assess the binding affinity and targeting ability of MEVs and LEVDs to GBM cells and macrophages, GL261 and RAW264.7 cells were seeded on slides in 6-well plates (3×10^5^ cells/well). PKH67-MEVs (10 μg mL^-1^) and LEVDs (DOX concentration: 1 μg mL^-1^) were added to the cells. At indicated time points, cellular uptake was evaluated by LSCM and flow cytometry.

GL261 and LN229 cells were also incubated with LEVDs or free DOX (DOX concentration: 2.5 μg mL^-1^) for 4 h, and cellular uptake was detected by flow cytometry.

To evaluate the targeting ability of individual components of the co-EVs delivery system MEVs/LEVDs to GBM cells and macrophages, GL261-mCherry (1.0×10^5^ cells/well) and RAW264.7 (3.0×10^5^ cells/well) cells were mixed and seeded in 6-well plates for direct contact co-culture. PKH67-MEVs (10 μg mL^-1^) and LEVDs (DOX concentration: 2.5 μg mL^-1^) were simultaneously. After 8 h of incubation, cells were harvested, and RAW264.7 cells were labeled with BV421-F4/80 primary antibody for 30 min at 4℃vin the dark. DOX and FITC (PKH67) signals in F4/80^+^ and mCherry^+^ cells were analyzed by flow cytometry.

To assess the in vivo targeting ability of MEVs or co-EVs delivery system MEVs/LEVDs to TAMs and GBM cells, orthotopic GL261-mCherry-bearing mice were generated as described. Mice received a single intravenous injection of PKH67-MEVs (5 μg g^-1^) or the co-EVs delivery system [PKH67-MEVs (5 μg g^-1^)/LEVDs (DOX dose: 2.5 μg g^-1^)]. At 12 h post-injection, mice were sacrificed, and GBM tissues were harvested. Single-cell suspensions were prepared by mincing tumors and digesting with type IV collagenase (500 μg mL^-1^, Thermo Fisher Scientific Inc.) and DNase I (10 μg mL^-1^, Thermo Fisher Scientific Inc.) for 3 h at 37 °C. The cell suspension was filtered through a 40 µm strainer, washed twice with PBS buffer (containing 2% FBS) by centrifuging at 350 g for 5 minutes. Red blood cells (RBC) were lysed with RBC lysis buffer for 5 min at room temperature. Cells were then stained with anti-CD11b and anti-F4/80 primary antibodies to label TAMs. Uptake of PKH-67-MEVs and LEVDs by TAMs (F4/80^+^CD11b^+^) or GBM cells (mCherry^+^) was detected by flow cytometry. Additionally, healthy tumor-free mice were injected with the co-EVs delivery system [PKH67-MEVs (5 μg g^-1^)/LEVDs (DOX dosage: 2.5 μg g^-1^)], and brain tissues were examined for PKH67 and DOX fluorescence by LSCM at 12 h post-injection.

**Cell viability assay**

Cytotoxicity was assessed using the CCK8 assay. To evaluate the cytotoxicity of MEVs, DOX, or LEVDs, GL261 and RAW264.7 cells were seeded in 96-well plates (4×10^3^ cells/well for MEVs; 1×10^4^ cells/well for DOX and LEVDs) and cultured for 24 h at 37°C. The medium was replaced with 200 μL fresh medium containing different concentrations of MEVs, DOX, or LEVDs, and cells were cultured for another 48 h. Subsequently, the medium was removed, cells were washed twice with PBS, and 100 μL fresh medium containing 10 μL CCK8 (DOJINDO, Japan) was added. After 4 h of incubation, absorbance was measured at 450 nm using a microplate reader (Synergy2, Bio-Tek, USA). Cell viability was expressed as a percentage of the absorbance relative to the control group (MEVs or DOX concentration: 0 μg mL^-1^). The experiments were repeated in triplicate, and representative results from three independent experiments are shown.

**Immunohistochemistry (IHC)**

Orthotopic GL261-Luc-bearing mice (*n* = 3) were treated via tail vein injection every 5 days for a total of 4 injections as described. Two days after the final injection, GBM tissues were harvested. IHC was performed as previously described [29]. For all markers except CD34, the number of positive cells was quantified in five random fields at 400× magnification. For CD34, the average number of microvessels was quantified in five random fields at 400× magnification. Antibodies used are listed in Table S5.

**In vivo biosafety evaluation**

For biosafety evaluation of the co-EVs delivery system MEVs/LEVDs, tumor-free C57BL/6J mice were injected with MEVs (5 μg g^-1^) plus LEVDs (DOX dose: 2.5 μg g^-1^) every 5 days for a total of six injections. Mice injected with PBS served as controls. Body weight was recorded every three days. Two days after the last administration, mice were euthanized. Serum was collected for biochemical analysis using a BS-330E Mindray biochemistry analyzer and for inflammatory cytokine detection using a Kaeser 6600 system (Kangrun Biotech, China). Serum cTnT concentration was measured using a FC-9000 biochemistry analyzer (Wondfo Biotech, China). Major organs (heart, liver, spleen, lung, kidney, and brain) were harvested for hematoxylin and eosin (H&E) staining.

**Echocardiography**

Cardiac function was evaluated by echocardiography (Vevo 2100 System, VisualSonics) fitted with a 30-MHz transducer two days after the final administration of MEVs (5 μg g^-1^) + LEVDs (DOX dosage: 2.5 μg g^-1^). Mice were anesthetized and placed in a supine position on a heated physiologic monitoring stage. Left ventricular fractional shortening (LVFS) and left ventricular ejection fraction (LVEF) were measured by two-dimensional guided M-mode echocardiography at the short axis view.

**Heart weight (HW)/body weight (BW)**

Two days after the final administration of MEVs + LEVDs, body weight was recorded. Mice were then euthanized, and hearts were harvested, dried, and weighed. The heart weight-to-body weight (HW/BW) ratio were calculated.

**Flow cytometry analysis of in vivo GBM samples**

Orthotopic GL261-Luc-bearing mice (n = 3 per group) were injected via tail vein every 5 days for a total of 4 injections as described. Two days after the final injection, GBM tissues were harvested and single-cell suspensions were prepared as described above. For flow cytometry, cells (1×10^6^) were first incubated with mouse Fc receptor blocker (BD biosciences, USA) for 10 min, then stained with Fixable Viability Stain 780 (BD biosciences, USA) and surface markers antibodies for 30 min at 4°C in the dark. For intracellular staining, cells were fixed and permeabilized using the Fixation/Permeabilization Solution Kit (BD biosciences, USA) and stained with intracellular antibodies for 30 min at 4°C. Stained cells were analyzed using a Fortessa flow cytometer (BD Biosciences) and FlowJo software (BD Biosciences). Antibodies are listed in Table S6.


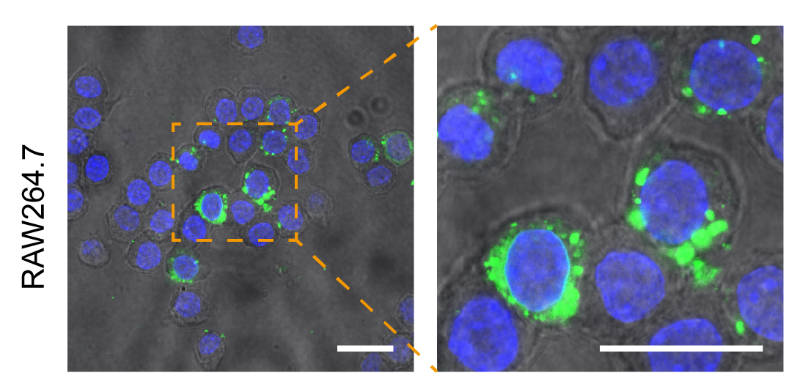


**Fig. S1.** LSCM images of cellular uptake of PKH67-MEVs in M0 RAW264.7 cells after incubation for 6 h. Scale bar: 20 µm.


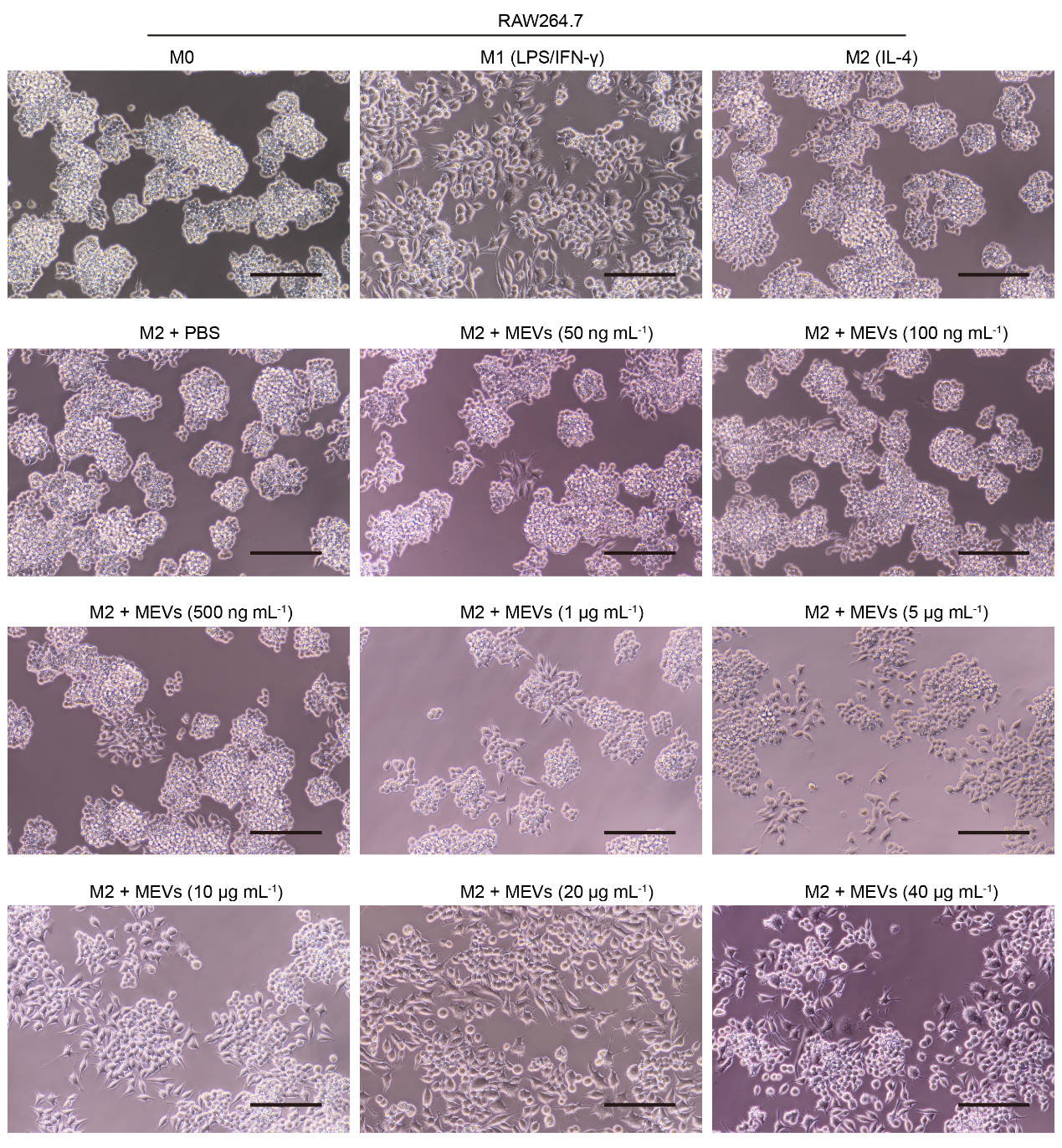


**Fig. S2.** Cell morphology of M2 RAW264.7 cells incubated with different concentrations of MEVs as indicated for 24 h, imaged by phase-contrast microscopy. Scale bar: 300 μm.


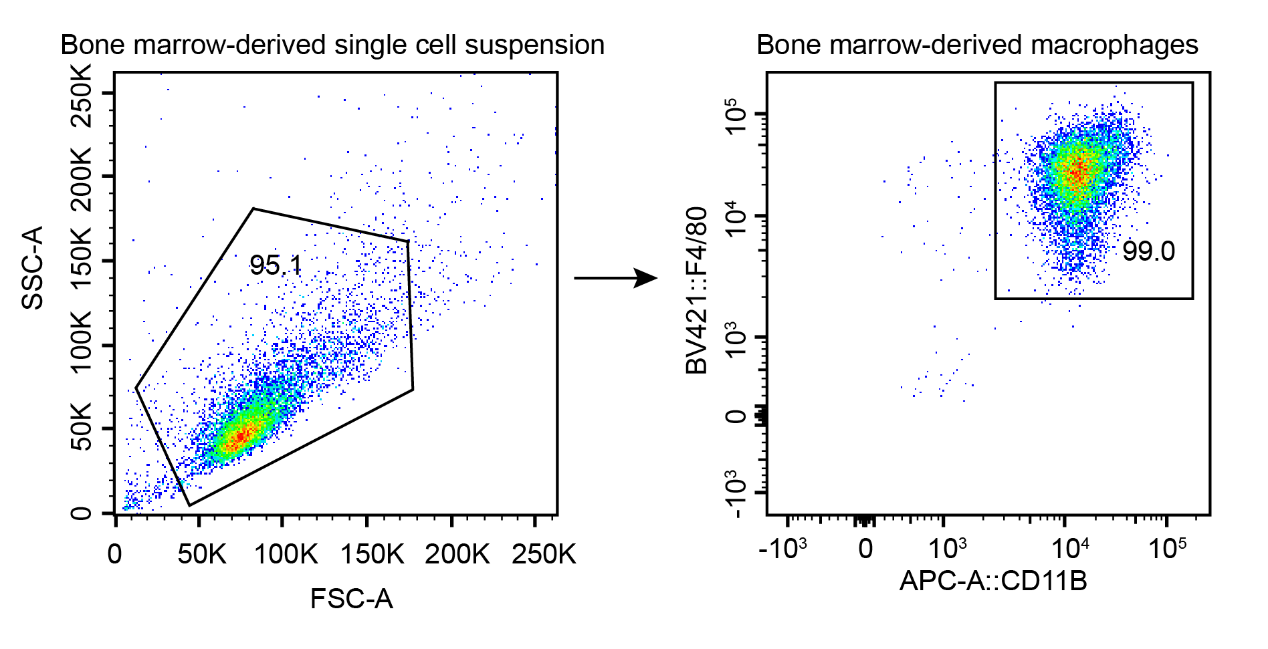


**Fig. S3.** Purity of mouse bone marrow-derived macrophages (BMDMs) (CD11b^+^F4/80^+^) assessed by flow cytometry.


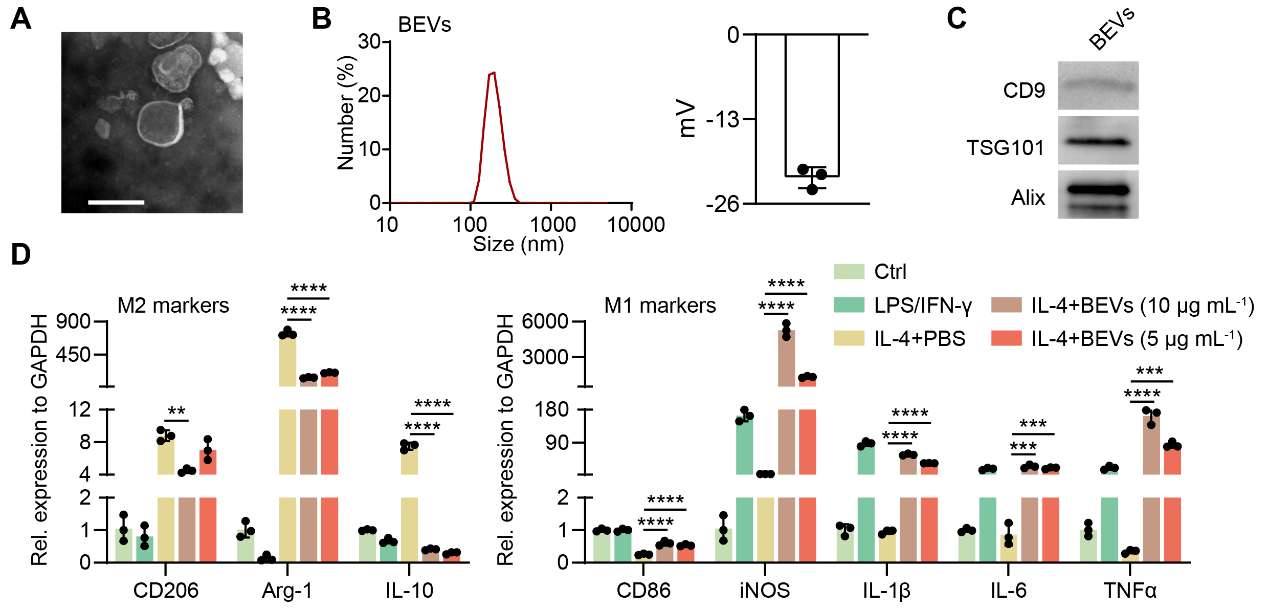


**Fig. S4.** M0 BMDMs-derived EVs (BEVs) repolarize M2 BMDMs toward an M1 phenotype. (A) TEM image of BEVs. Scale bar: 100 nm. (B) Size distribution (hydrodynamic diameter) and zeta potential of BEVs detected by DLS. (C) Western blot analysis of EVs markers CD9, TSG101 and Alix in BEVs. (D) Quantitative reverse transcription polymerase chain reaction analysis of M2 (left panel) and M1 (right panel) markers in BEVs-treated M2 BMDMs; LPS/IFN-γ-treated BMDMs (M1 BMDMs) served as positive control (*n* = 3). Expression normalized to GAPDH. The results indicated that M2 markers CD206 and IL-10 were remarkably downregulated, while M1 markers CD86, iNOS, IL-6, IL-1β, TNF-α and CXCL10 were significantly upregulated in BEVs-treated M2 BMDMs. Data are represented as mean ± SD. Statistical significance was determined using an unpaired, 2-sided Student’s t test (D), ***P* < 0.01; ****P* < 0.001; *****P* < 0.0001. GAPDH, glyceraldehyde phosphate dehydrogenase.


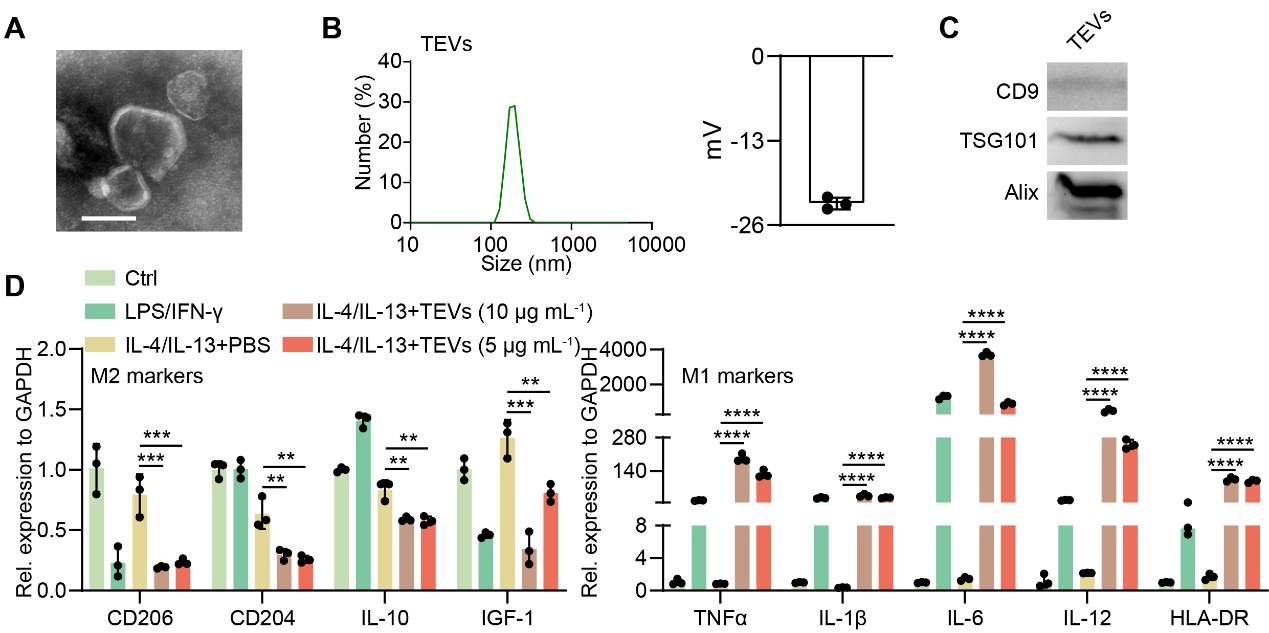


**Fig. S5.** M0 THP-1-derived EVs (TEVs) repolarize M2 THP-1 cells toward an M1 phenotype. (A) TEM image of TEVs. Scale bar: 100 nm. (B) Size distribution and zeta potential of TEVs measured by DLS. (C) Western blot analysis of EVs markers CD9, TSG101, and Alix in TEVs. (D) Quantitative reverse transcription polymerase chain reaction analysis of M2 (left) and M1 (right) markers in TEVs-treated M2 THP-1 cells; LPS/IFN-γ-treated THP-1 cells (M1 THP-1) served as positive control (*n* = 3). Expression normalized to GAPDH. Data are represented as mean ± SD. Statistical significance was determined using an unpaired, 2-sided Student’s t test (D), ***P* < 0.01; ****P* < 0.001; *****P* < 0.0001. GAPDH, glyceraldehyde phosphate dehydrogenase.


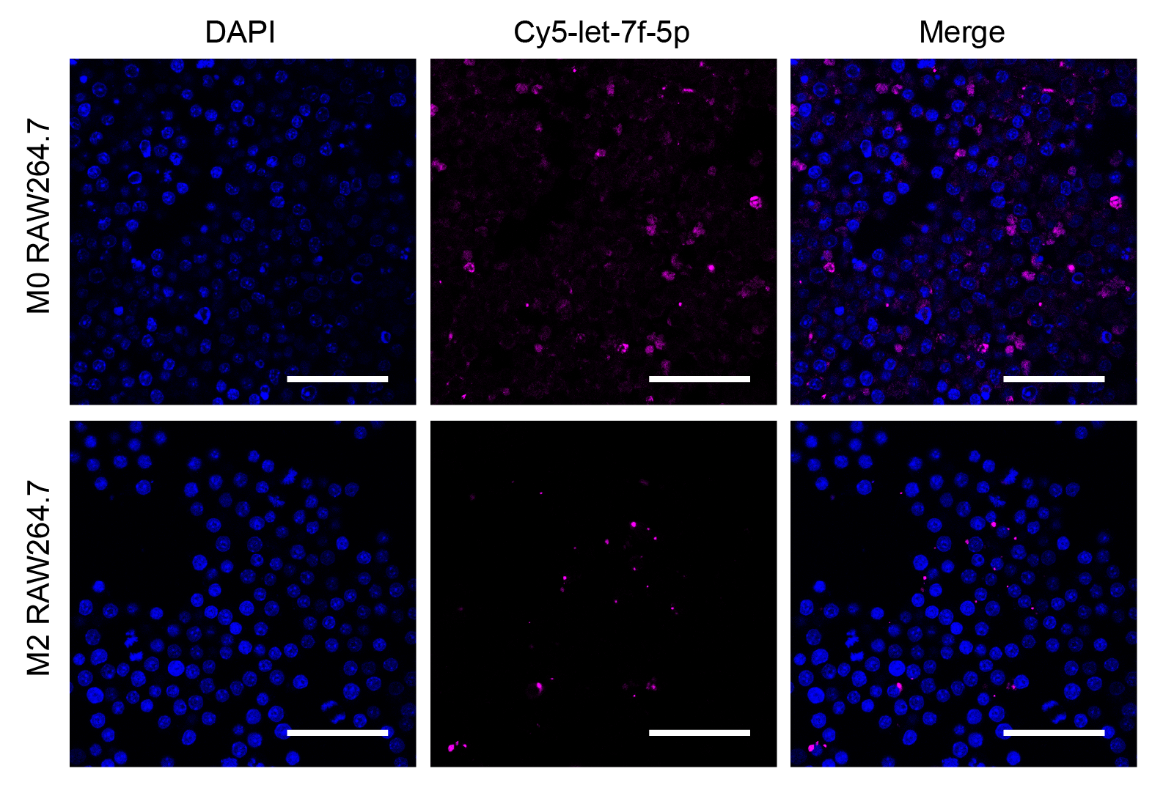


**Fig. S6.** MicroRNA let-7f-5p can be transferred to M2 RAW264.7 cells via MEVs secreted by M0 RAW264.7 cells. M0 RAW264.7 cells were first transfected with Cy5-labeled let-7f-5p mimic (Cy5-let-7f-5p). MEVs were isolated from the supernatant 48 h post-transfection and added to M2 RAW264.7 cells. Cy5 fluorescence in M0 and M2 RAW264.7 cells was imaged by LSCM after 24 h. Scale bar: 60 μm.


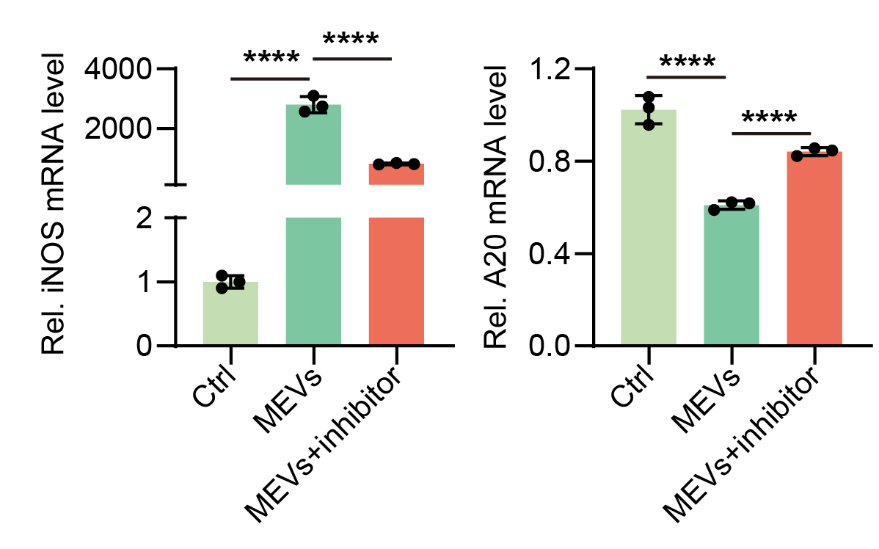


**Fig. S7.** Quantitative reverse transcription polymerase chain reaction analysis of iNOS mRNA expression in MEVs-treated M2 RAW264.7 cells in the presence or absence of let-7f-5p inhibitor (*n* = 3). Data are represented as mean ± SD. Statistical significance was determined using one-way ANOVA followed by Bonferroni’s post hoc test, *****P* < 0.0001.


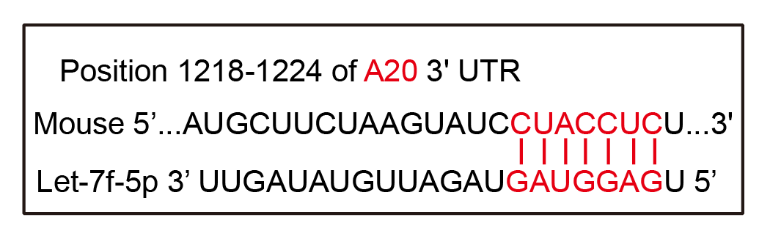


**Fig. S8.** Predicted binding site of let-7f-5p with the 3’-UTR of A20 using TargetScan Mouse 8.0.


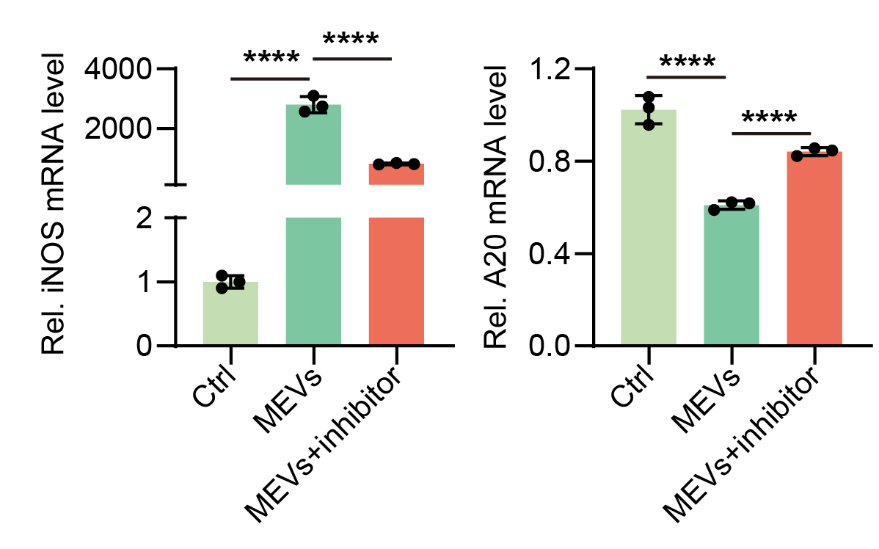


**Fig. S9.** Quantitative reverse transcription polymerase chain reaction analysis of A20 mRNA expression in MEVs-treated M2 RAW264.7 cells in the presence or absence of let-7f-5p inhibitor (*n* = 3). Data are represented as mean ± SD. Statistical significance was determined using one-way ANOVA followed by Bonferroni’s post hoc test, *****P* < 0.0001.


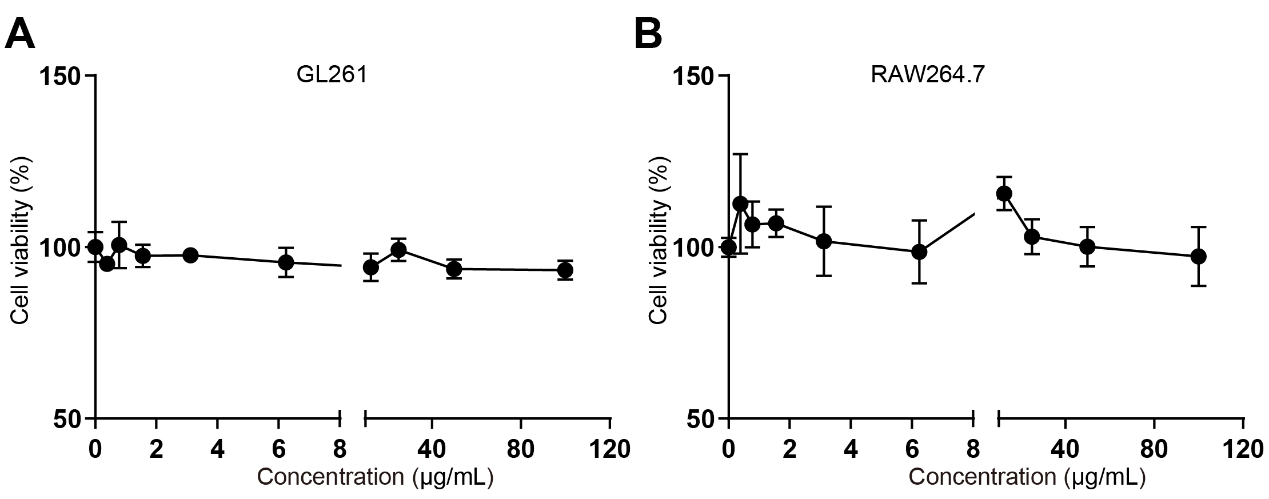


**Fig. S10.** Cell viability of GL261 (**A**) and RAW264.7 cells (**B**) treated with different concentrations of MEVs for 48 h (*n* = 4). Data are represented as mean ± SD.


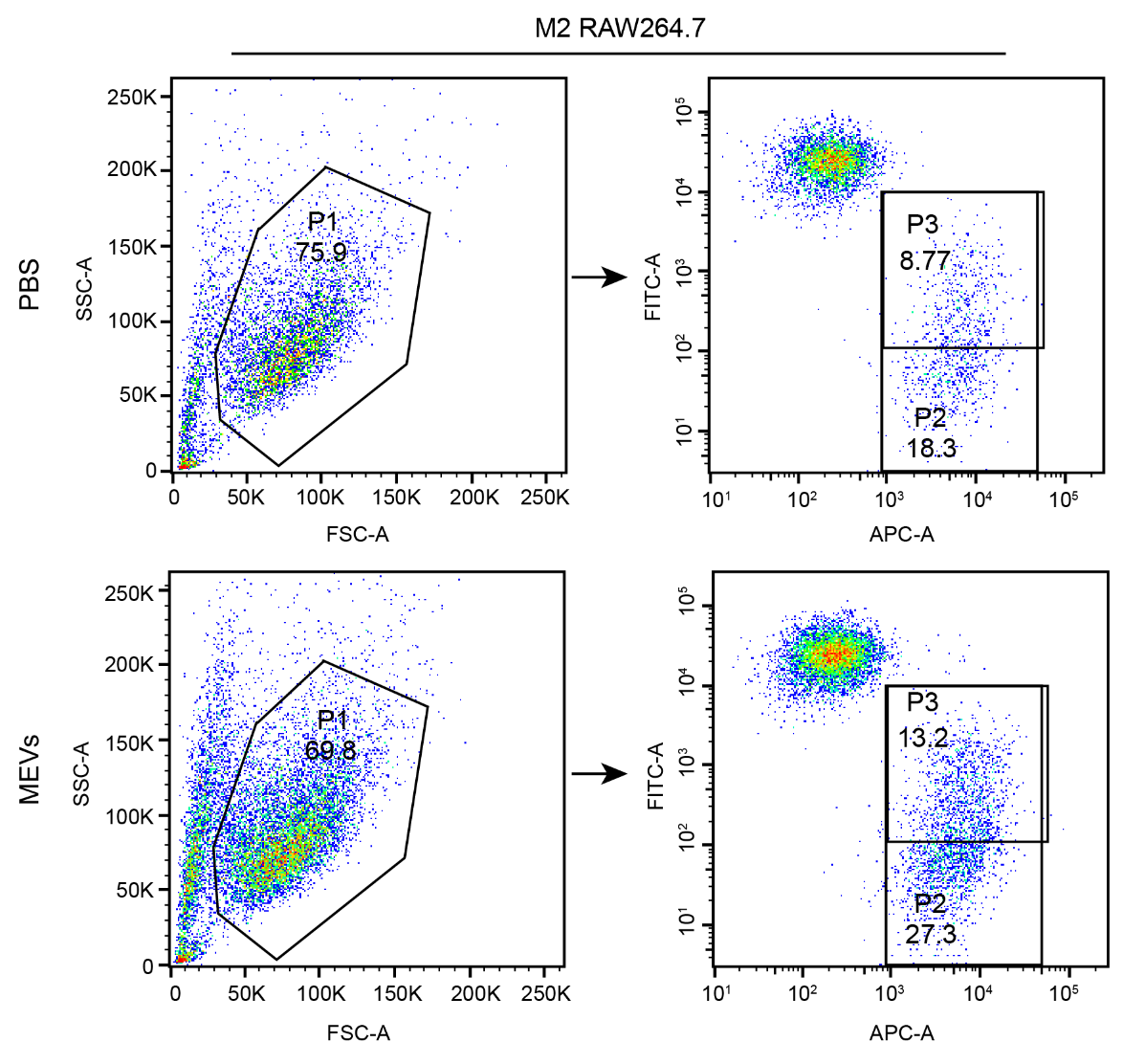


**Fig. S11.** Phagocytosis of GL261 cells by MEVs-treated M2 RAW264.7 cells evaluated by flow cytometry (n = 3). M0 RAW264.7 cells were polarized to an M2 phenotype, treated with MEVs or PBS for 24 h, and then incubated with CFSE labeled-GL261 (GL261-CFSE) cells for additional 12 h. Cells were subsequently harvested and stained with APC-anti-CD11b primary antibody. Percentage of APC^+^CFSE^+^ double-positive cells determined by flow cytometry.


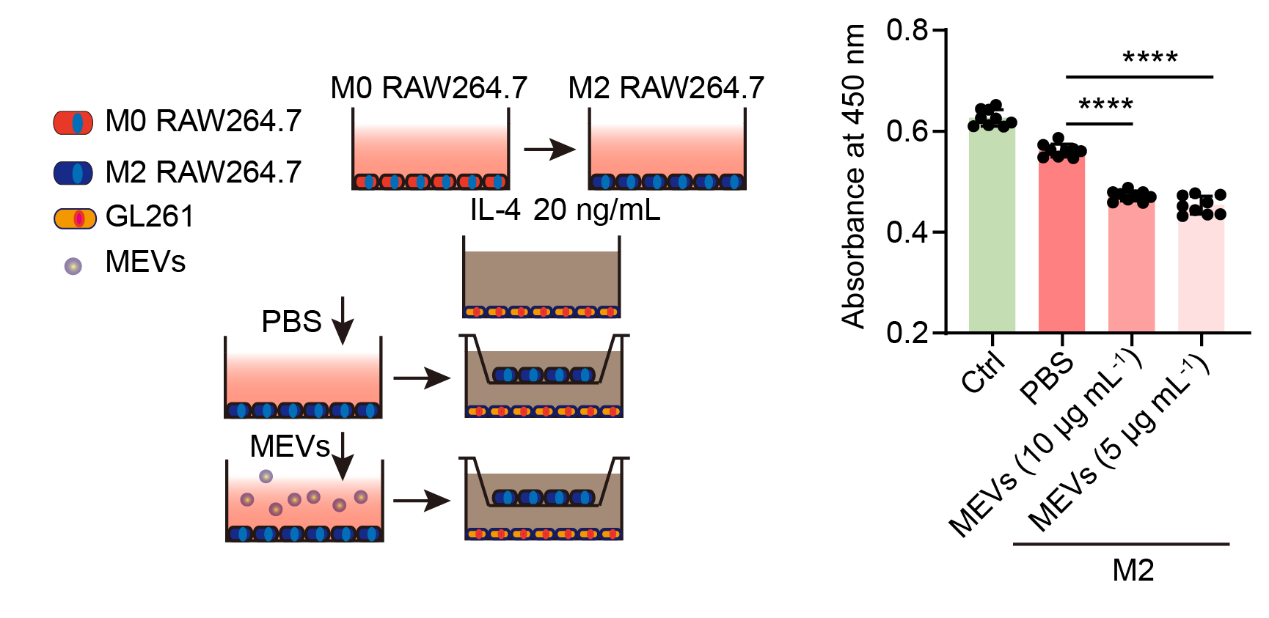


**Fig. S12.** Schematic illustration of the non-contact co-culture system of GL261 and RAW264.7 cells using a transwell model. M0 RAW264.7 cells were polarized to M2 macrophages with IL-4 (20 ng mL^-1^) for 24 h, then treated with PBS or different concentrations of MEVs for another 24 h. Treated M2 RAW264.7 cells were seeded in the upper compartment and co-cultured with GL261 cells in the lower compartment for 36 h; GL261 proliferation was determined by CCK8 assay.


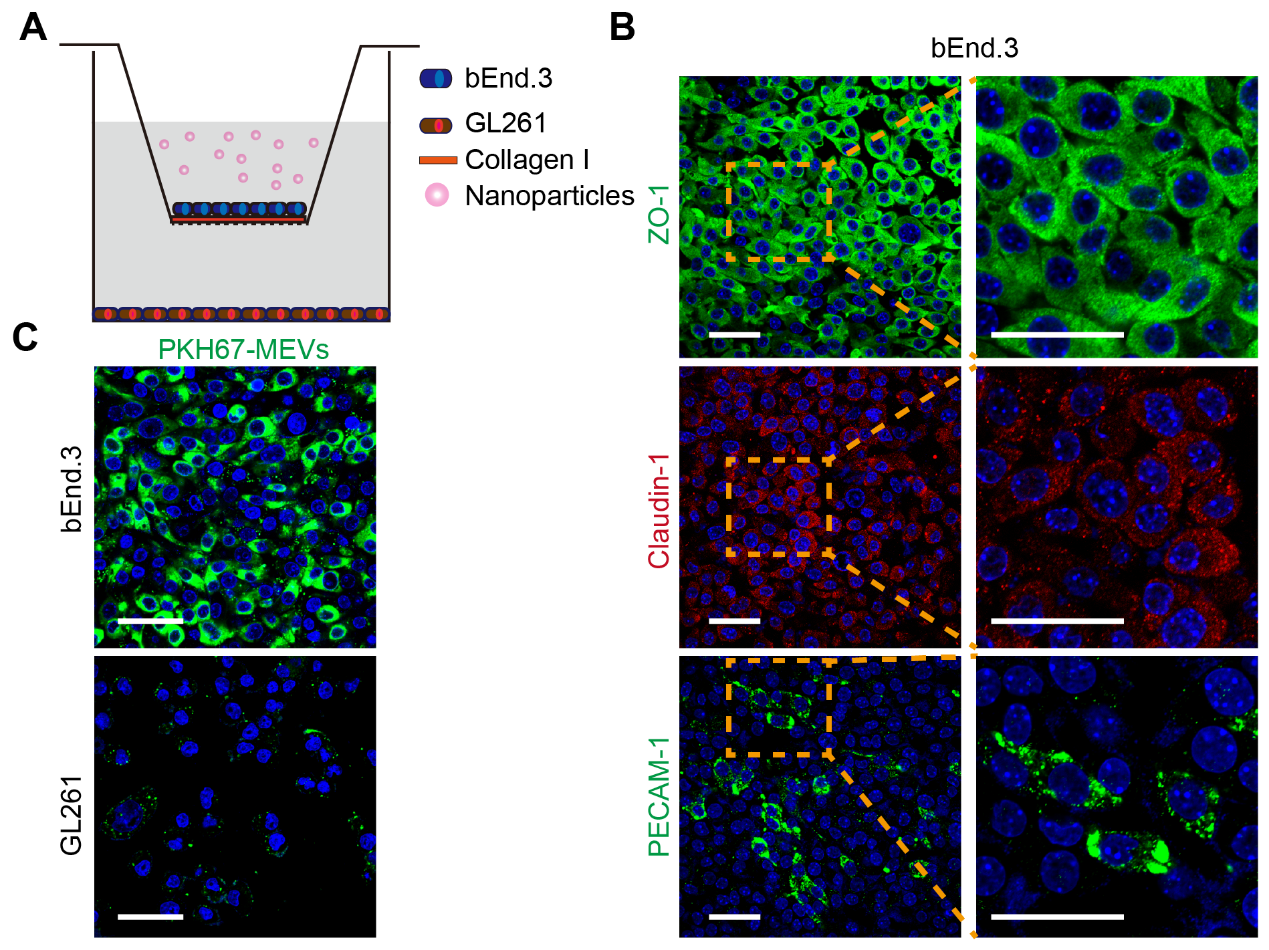


**Fig. S13.** MEVs efficiently cross the in vitro BBB. (A) Schematic of in vitro BBB model construction. (B) Immunofluorescence detection of ZO-1, Claudin-1, and PECAM-1 in bEnd.3 monolayers. Scale bar: 20 μm. (C) LSCM images of PKH67-MEVs permeability in bEnd.3 and GL261 cells in the in vitro BBB model. Scale bar: 50 μm.


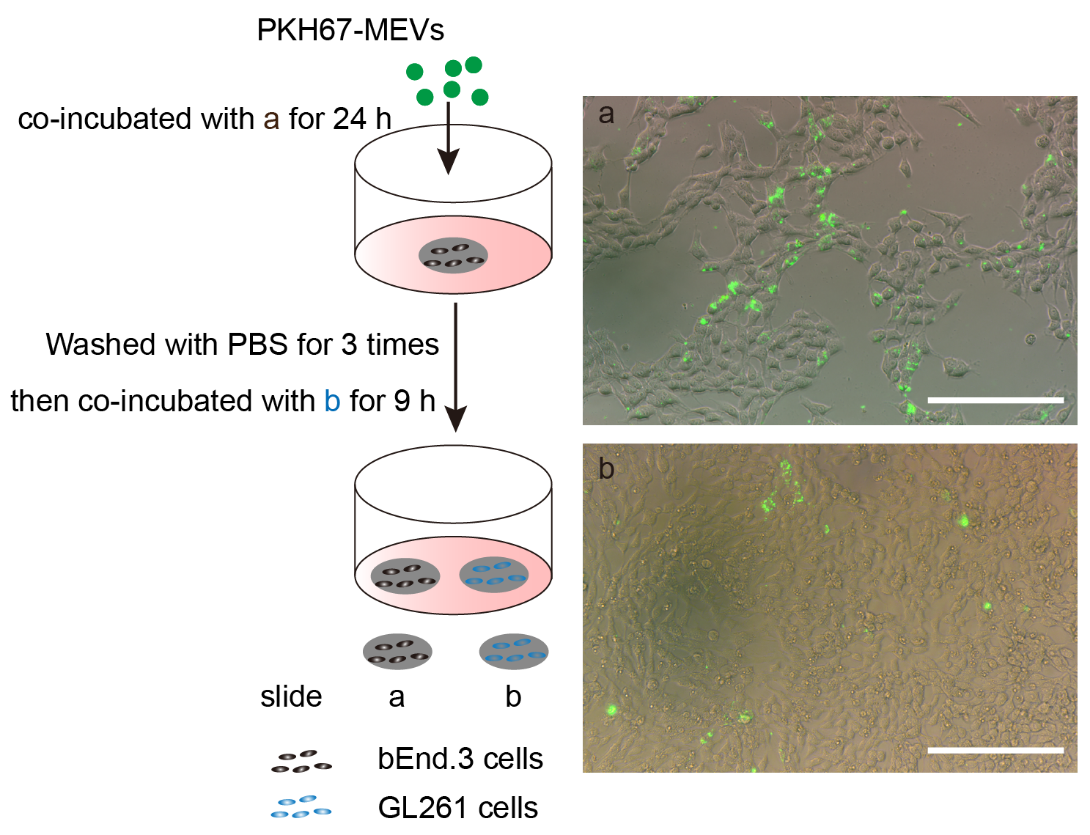


**Fig. S14.** Co-culture system showing transcytosis of MEVs from bEnd.3 cells to GL261 cells. Scale bar: 100 μm.


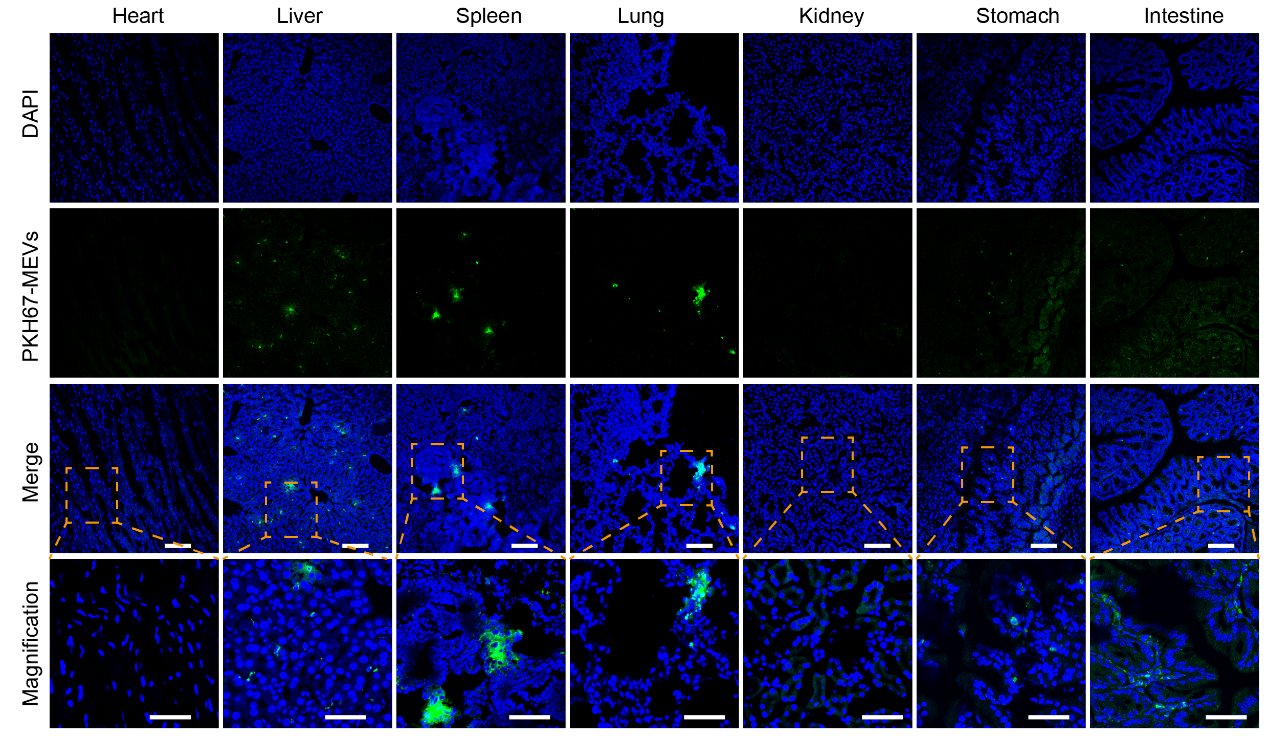


**Fig. S15.** Accumulation of MEVs in major organs imaged by LSCM. PKH67-MEVs were administered intravenously to orthotopic GL261-mCherry-bearing C57BL/6J mice. PKH67 signals in heart, liver, spleen, lung, kidney, stomach, and intestine were detected by LSCM at 12 h post-administration. Scale bar: merge: 100 μm; magnification: 50 μm.


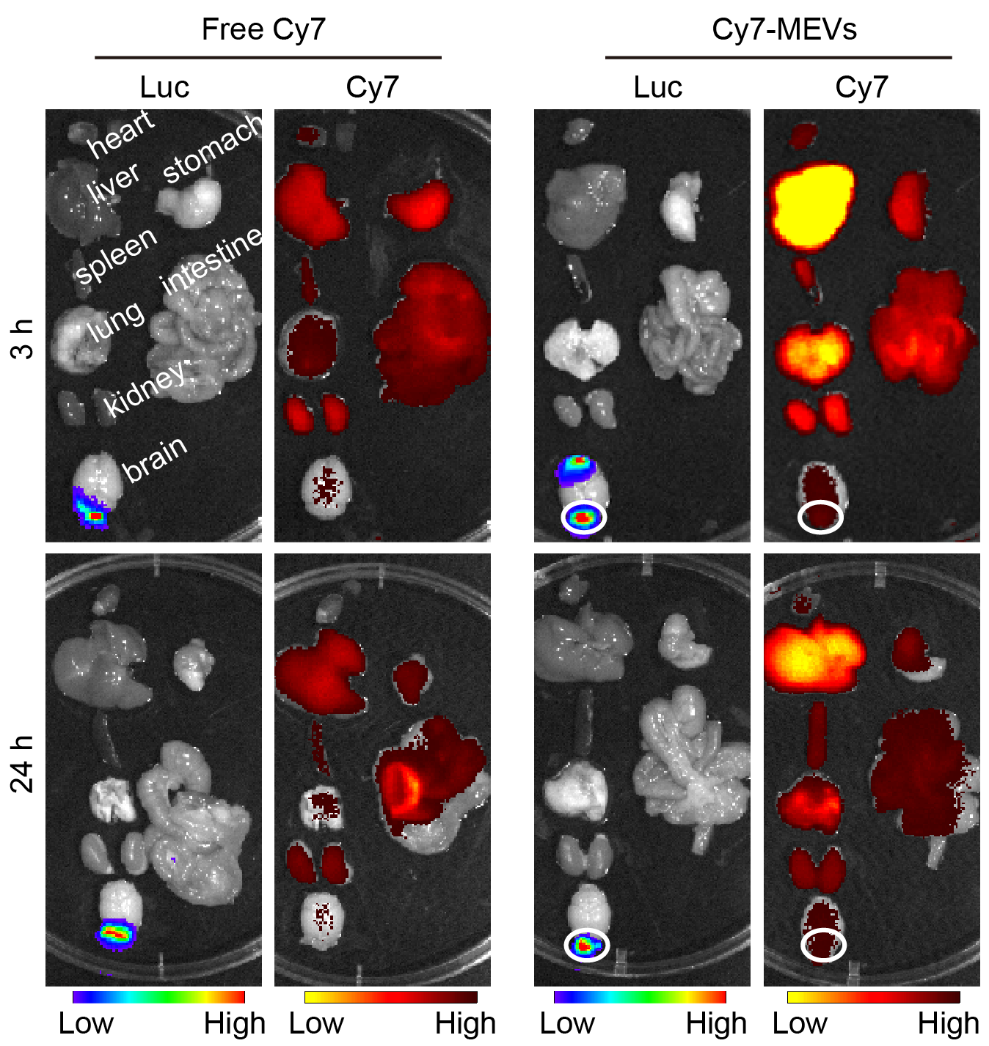


**Fig. S16.** Biodistribution of MEVs detected by IVIS. MEVs were labeled with Cy7 dye and injected intravenously into orthotopic GL261-Luc-bearing C57BL/6J mice. At indicated time points, mice were sacrificed, and Cy7 fluorescence and luciferase signals in brain and major organs were visualized. White circle indicates Cy7-MEVs colocalized with GL261-Luc signal in GBM.


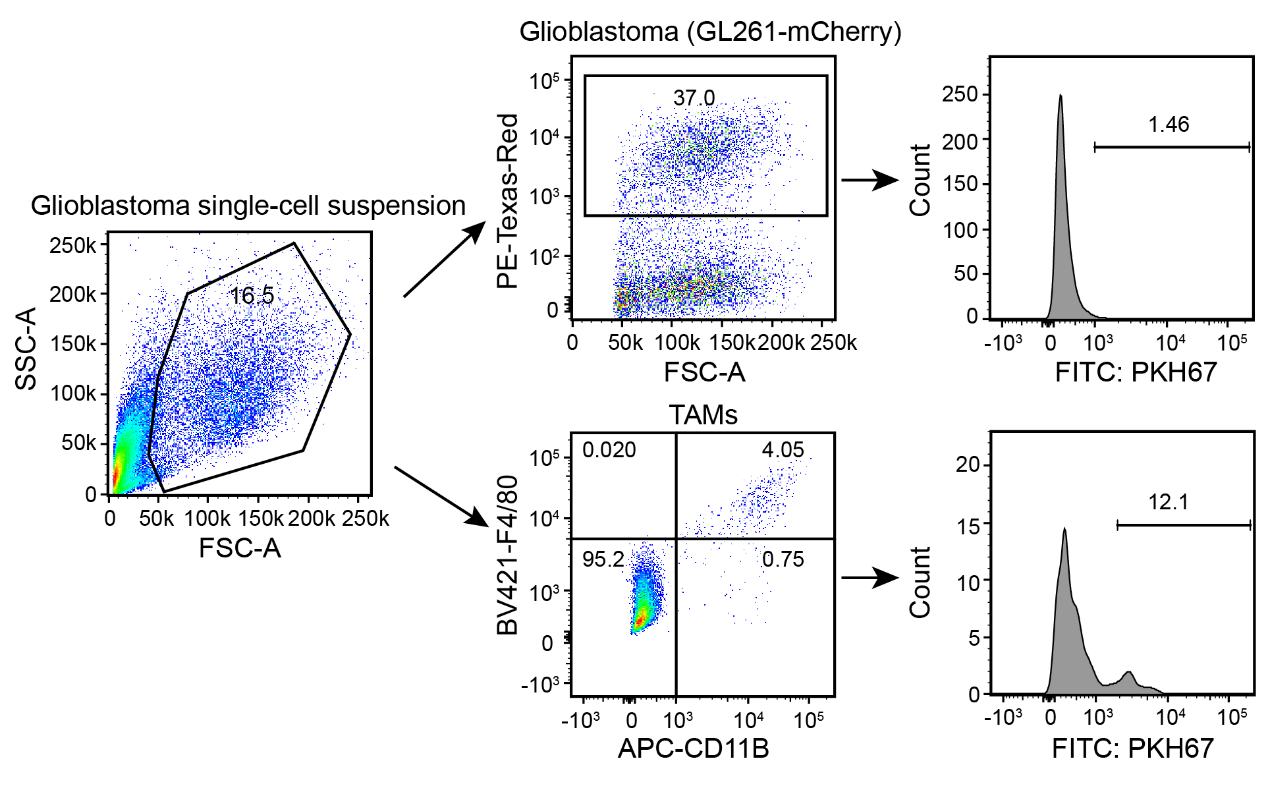


**Fig. S17.** In vivo targeting ability of MEVs to TAMs assessed by flow cytometry. Orthotopic GL261-mCherry-bearing C57BL/6J mice were intravenously injected with a single dose of PKH67-MEVs. GBM was harvested at 12 h post-injection, and single-cell suspensions were prepared. Uptake of PKH67-MEVs by TAMs (F4/80^+^CD11b^+^) and GBM cells (mCherry^+^) was detected by flow cytometry.


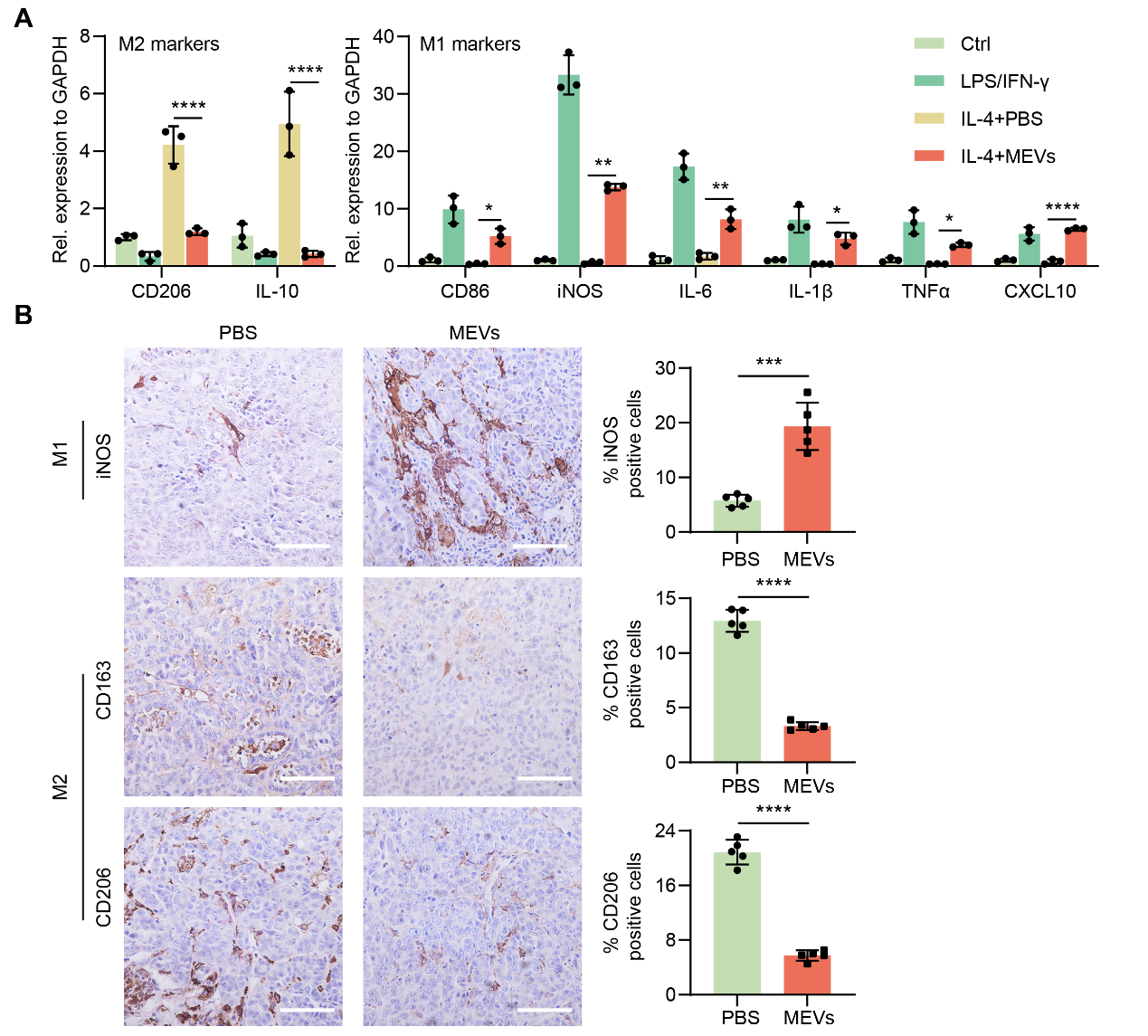


**Fig. S18.** MEVs repolarize M2 macrophages to an M1 phenotype in vivo. (A) Quantitative reverse transcription polymerase chain reaction analysis of M2 and M1 markers in MEVs-treated M2 BMDMs (*n* = 3). BMDMs were polarized to M2 with IL-4 and treated with MEVs or PBS for 24 h. LPS/IFN-γ-induced BMDMs (M1 BMDMs) served as positive control. (B) IHC analysis of M1 (iNOS) and M2 (CD163 and CD206) markers in GL261-bearing subcutaneous tumors after intratumoral injection of MEVs (*n* = 5). Scale bar: 100 μm. Data are represented as mean ± SD. Statistical significance was determined using an unpaired, two-sided Student’s t test, **P* < 0.05; ***P* < 0.01; ****P* < 0.001; *****P* < 0.0001. GAPDH, glyceraldehyde phosphate dehydrogenase.


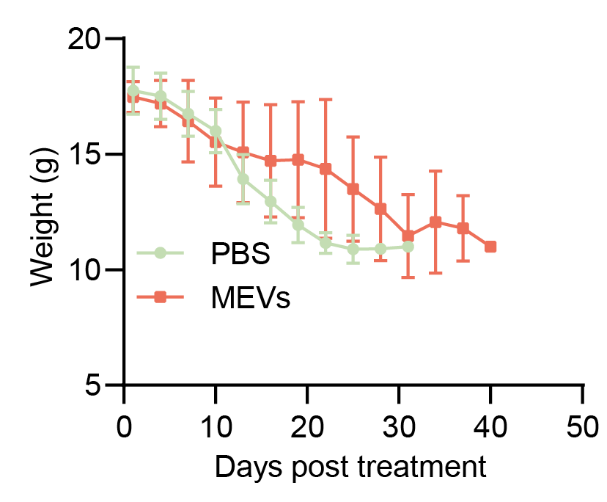


**Fig. S19.** Body weight of GL261-Luc-bearing C57BL/6J mice in different treatment groups (*n* = 6). Data are represented as mean ± SD.


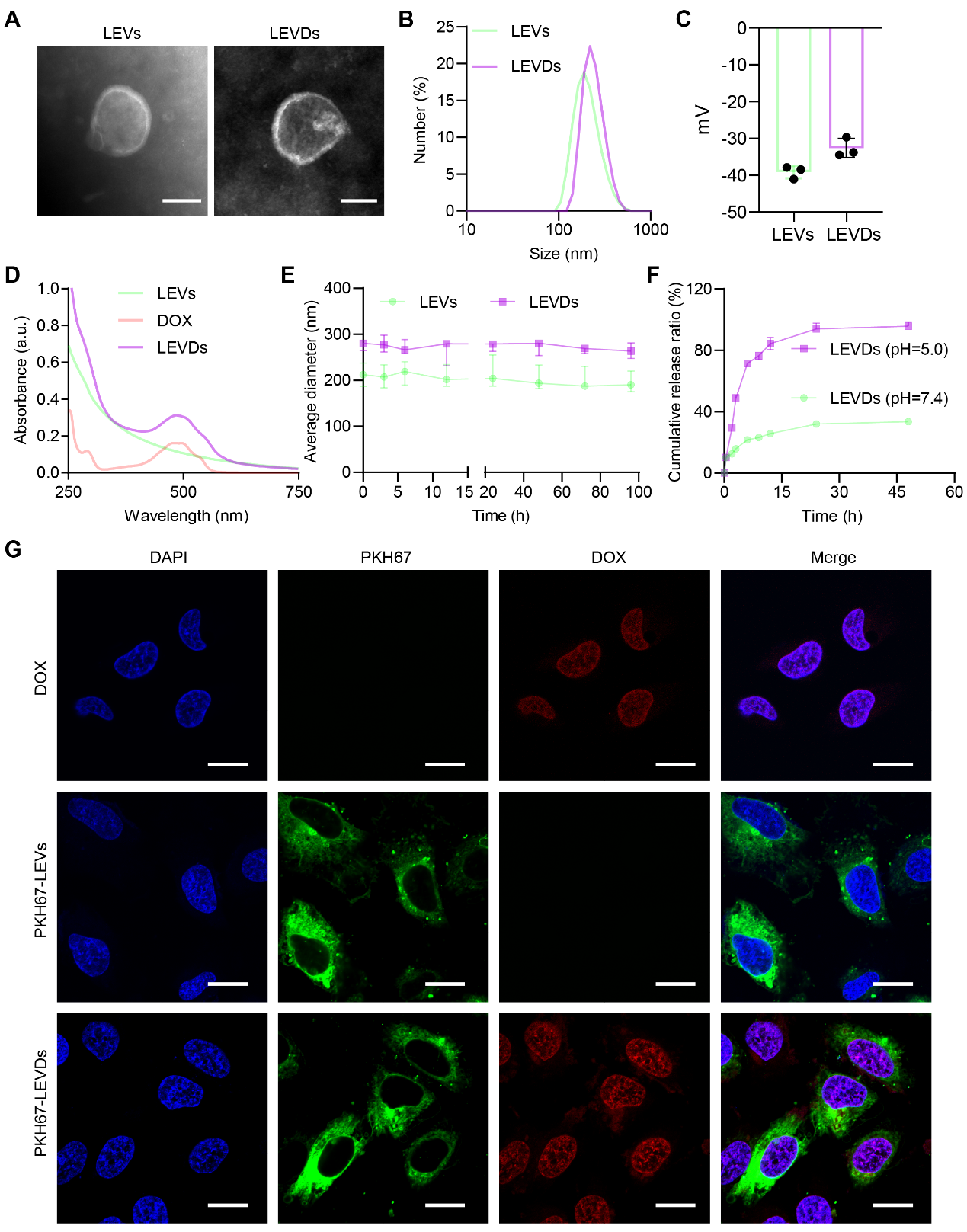


**Fig. S20.** (A) TEM images of lemon-derived EVs and LEVDs. Scale bar: 100 nm. (B) Size distribution (hydrodynamic diameter) of LEVs and LEVDs measured by DLS. (C) Zeta potential of LEVs and LEVDs (*n* = 3). (D) UV-vis spectra of LEVs, DOX, and LEVDs. (E) Stability of LEVs and LEVDs in 50% FBS at different time points (*n* = 3). (F) Cumulative DOX release from LEVDs at pH 5.0 and pH 7.4 (*n* = 3). (G) LSCM images of subcellular localization of DOX, PKH67-LEVs, and PKH67-LEVDs in GL261 cells after 4 h incubation. Scale bar: 20 μm. Data are represented as mean ± SD.


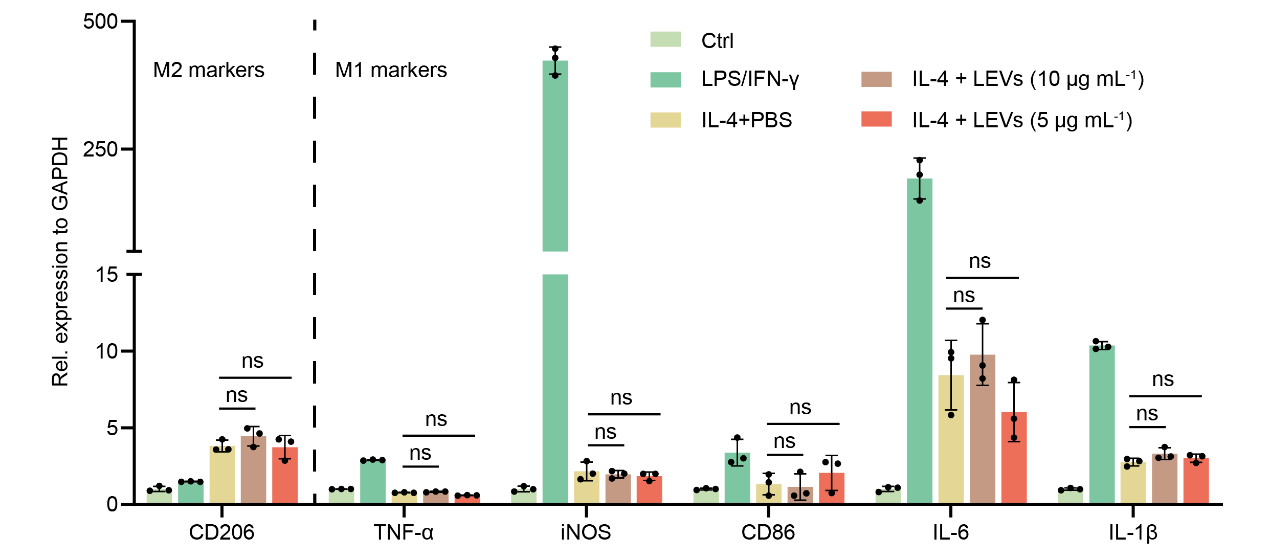


**Fig. S21.** Quantitative reverse transcription polymerase chain reaction analysis of M2 and M1 markers in M2 RAW264.7 cells treated with different concentrations of LEVs (*n* = 3). LPS/IFN-γ-treated RAW264.7 cells (M1 RAW264.7) served as positive control. Data are represented as mean ± SD. Statistical significance was determined using an unpaired, 2-sided Student’s t test, ns: not significant. GAPDH, glyceraldehyde phosphate dehydrogenase.


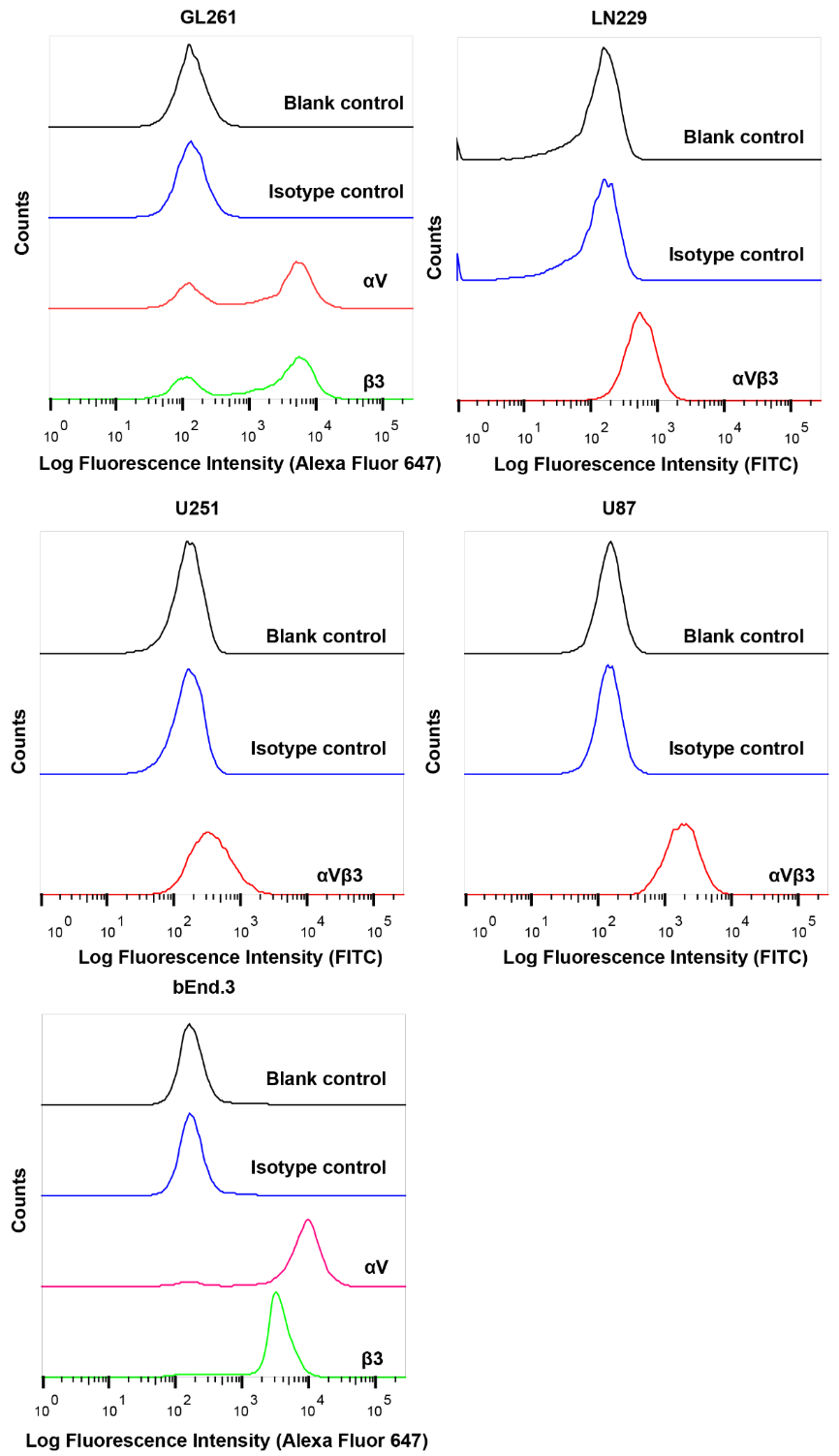


**Fig. S22.** Flow cytometric analysis of integrin αV and β3 expression in GBM and bEnd.3 cells.


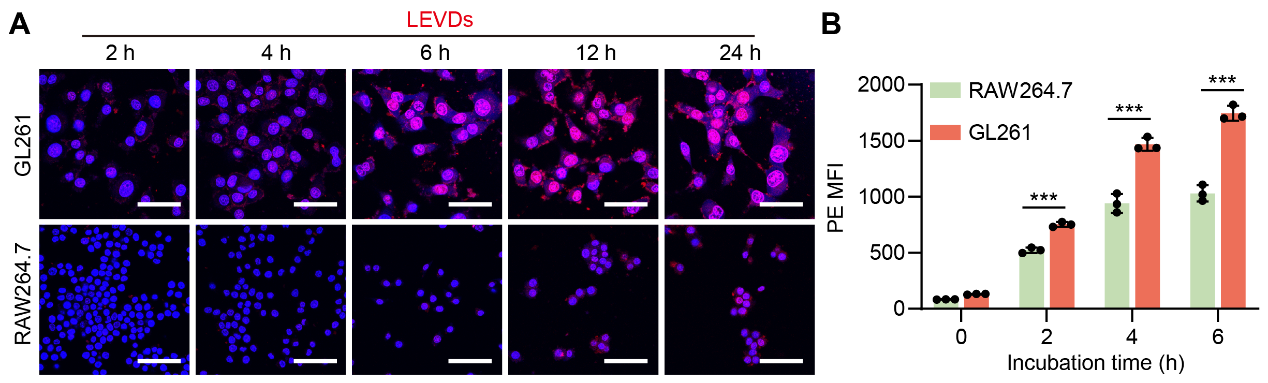


**Fig. S23.** Cellular uptake of LEVDs in GL261 and RAW264.7 cells detected by LSCM and flow cytometry. (A) LSCM images of LEVDs uptake at indicated time points. Scale bar: 50 μm. (B) Flow cytometric analysis of LEVDs uptake at indicated time points (*n* = 3). Data are represented as mean ± SD. Statistical significance was determined using an unpaired, 2-sided Student’s t test, ****P* < 0.001.


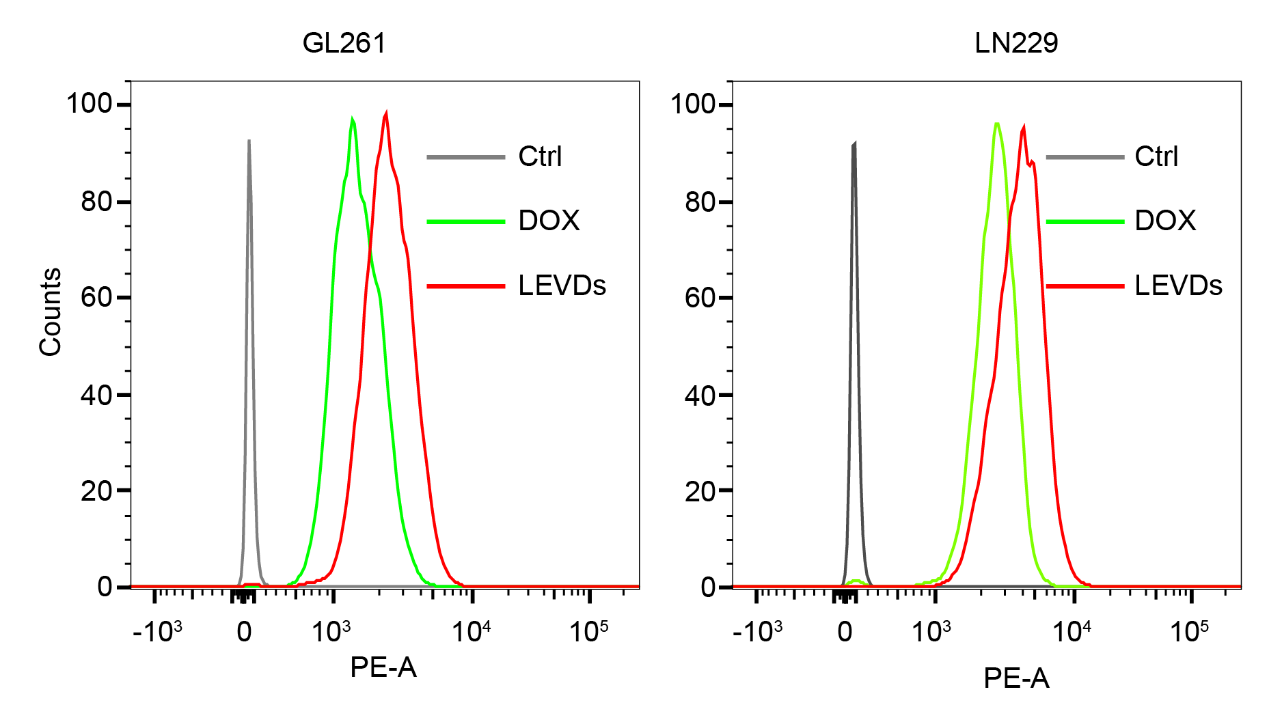


**Fig. S24.** Cellular uptake of DOX and LEVDs in GBM cells determined by flow cytometry.


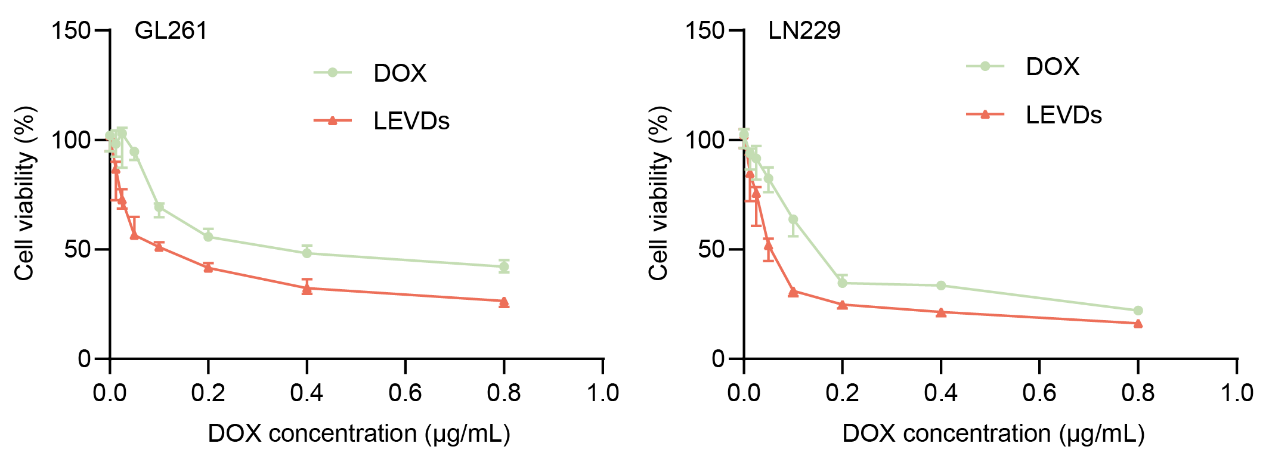


**Fig. S25.** Cell viability of GL261 and LN229 cells treated with DOX or LEVDs for 48 h (*n* = 5). Data are represented as mean ± SD.


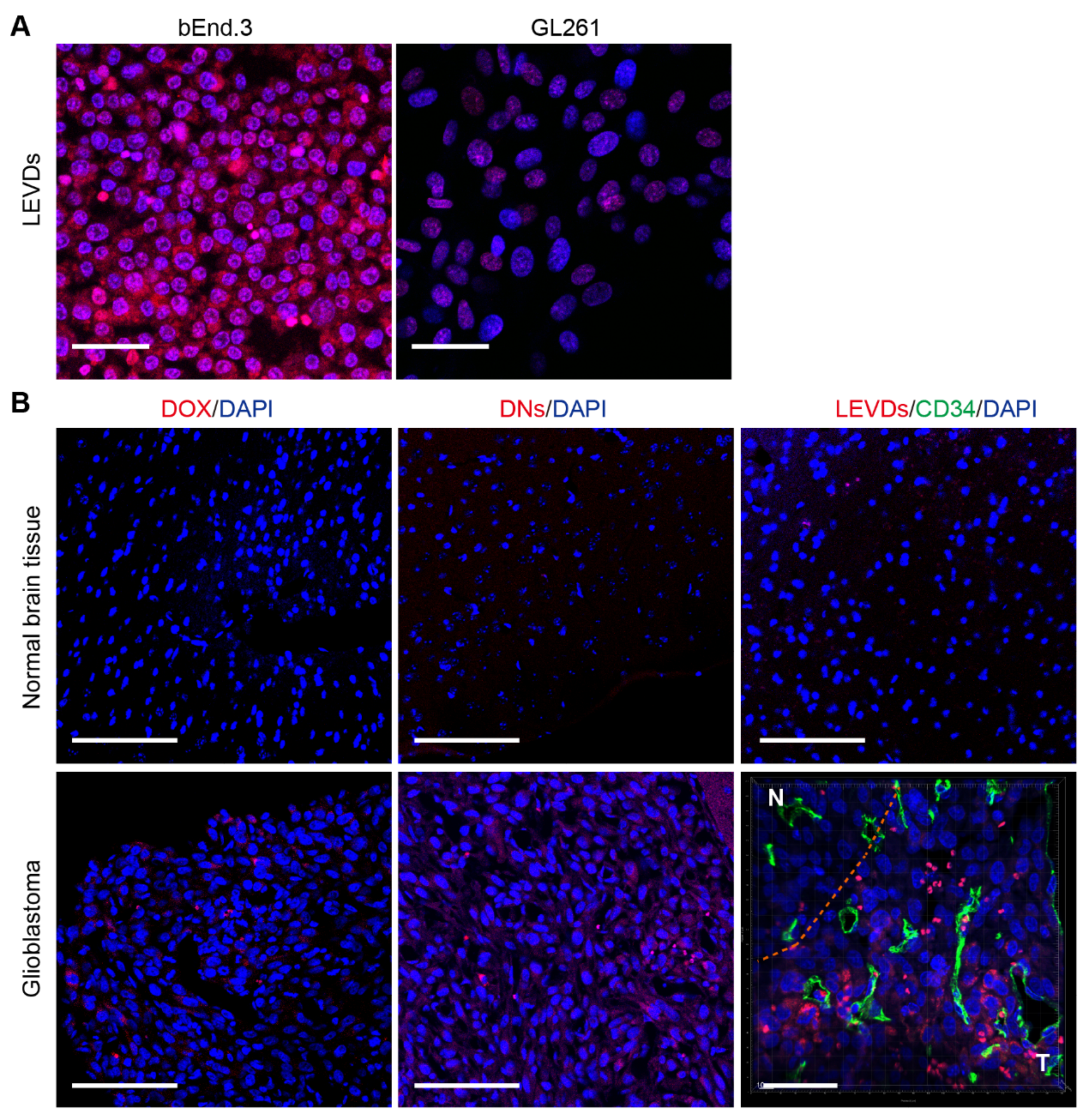


**Fig. S26.** LEVDs efficiently penetrate BBB/BBTB in vitro and in vivo. (A) LSCM images of LEVDs permeability in bEnd.3 and GL261 cells in the in vitro BBB model. Scale bar: 50 μm. (B) LSCM images of healthy and orthotopic GL261-bearing brain tissues after intravenous injection of DOX, DNs, or LEVDs. Endothelial cells stained with CD34 (green), nuclei with DAPI, and LEVDs (red) extravasated from tumor vessels and accumulated in tumor tissue. T, tumor tissue; N, normal tissue. Scale bar: 50 μm.


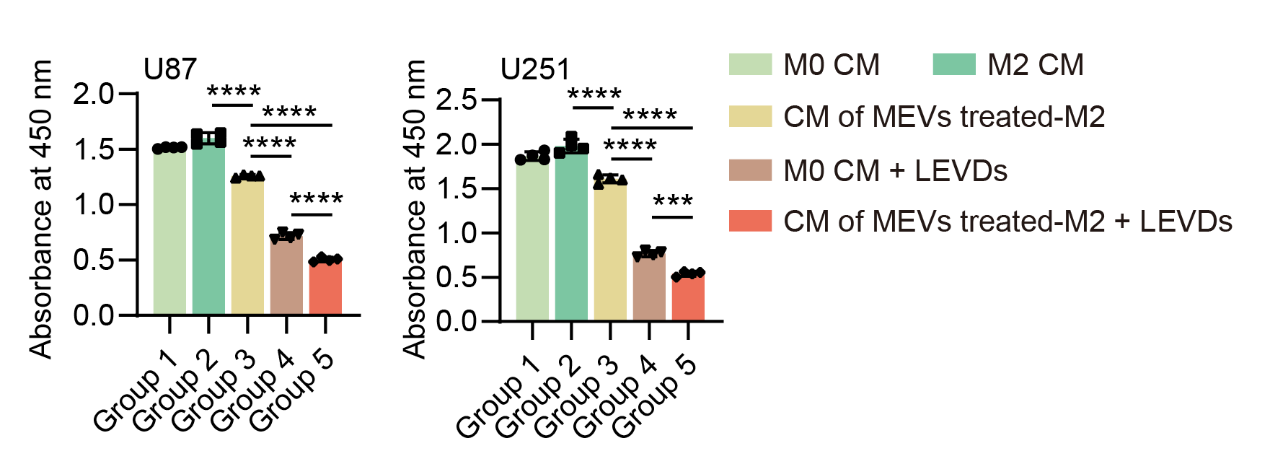


**Fig. S27.** Proliferation of U87 and U251 cells treated as indicated, detected by CCK8 assay (*n* = 4). Data are represented as mean ± SD. Statistical significance was determined using one-way ANOVA followed by Bonferroni’s post hoc test, ****P* < 0.001; *****P* < 0.0001.


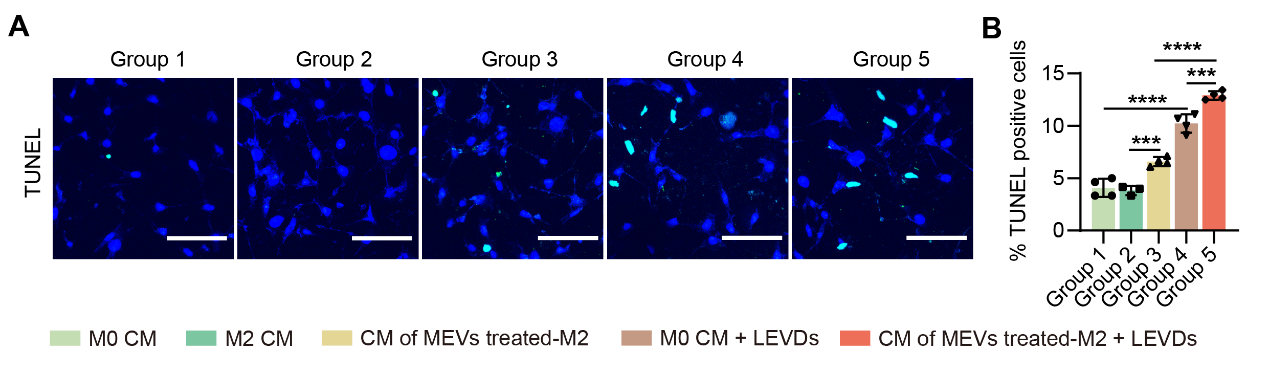


**Fig. S28.** The co-EVs delivery system MEVs/LEVDs promotes GBM cell apoptosis in vitro. (A) Representative TUNEL staining images of GL261 cells from different treatment groups. Group 1: M0 conditioned medium (CM); Group 2: M2 CM; Group 3: CM of MEVs-treated M2 RAW264.7 cells; Group 4: M0 CM + LEVDs; Group 5: CM of MEVs-treated M2 RAW264.7 cells + LEVDs. Scale bar: 100 μm. (B) Quantitative analysis of (A) (*n* = 4). Data are represented as mean ± SD. Statistical significance was determined using one-way ANOVA followed by Bonferroni’s post hoc test, ****P* < 0.001; *****P* < 0.0001.


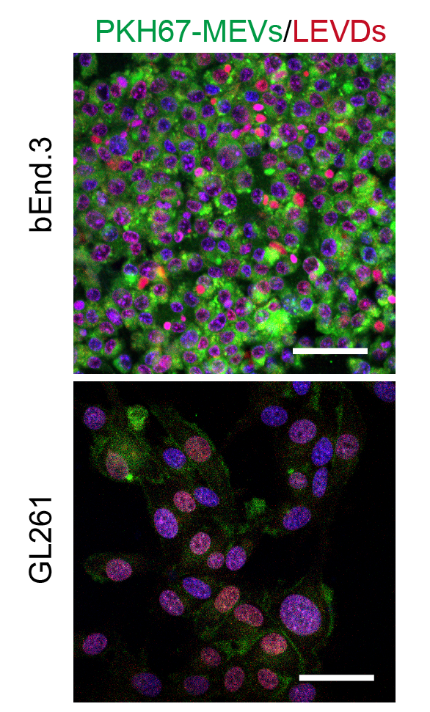


**Fig. S29.** LSCM images of co-EVs delivery system (PKH67-MEVs/LEVDs) permeability in bEnd.3 and GL261 cells in the in vitro BBB model. Scale bar: 50 μm.


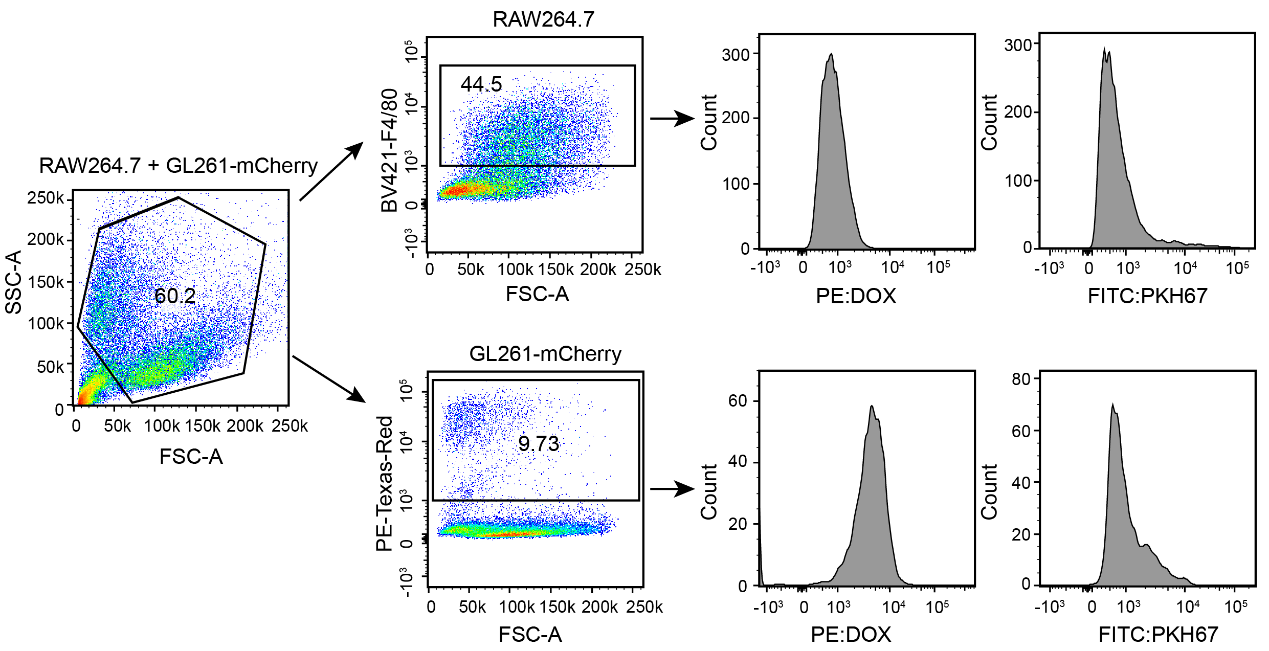


**Fig. S30.** Targeting ability of co-EVs delivery system MEVs/LEVDs to GBM cells and macrophages in an in vitro direct contact co-culture system, evaluated by flow cytometry. RAW264.7 and GL261-mCherry cells were mixed and seeded, and LEVDs and PKH67-MEVs were added simultaneously. After 8 h, cells were harvested, and RAW264.7 cells were labeled with BV421-anti-F4/80 antibody. DOX and PKH67 signals in F4/80^+^ and mCherry^+^ cells were determined by flow cytometry.


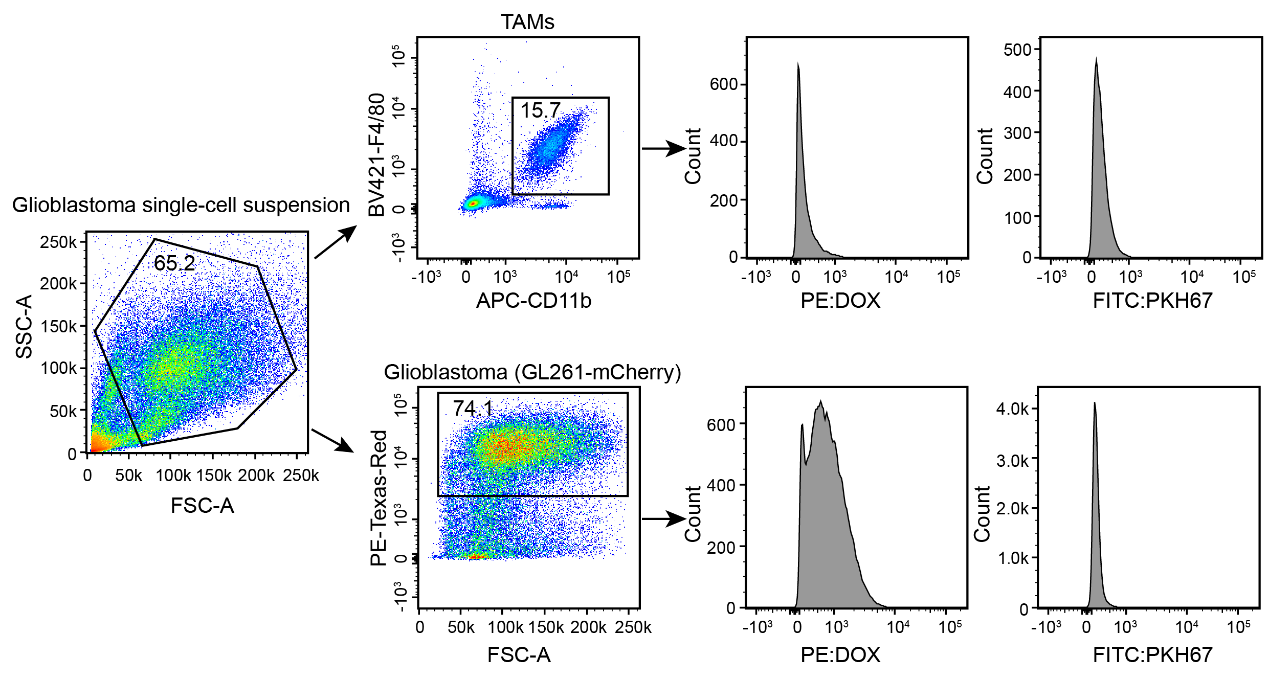


**Fig. S31.** In vivo targeting ability of co-EVs delivery system MEVs/LEVDs to TAMs and GBM cells, evaluated by flow cytometry. GL261-mCherry-bearing mice were injected with a single dose of PKH67-MEVs (5 μg g⁻¹) and LEVDs (DOX dose: 2.5 μg g⁻¹). GBM was harvested at 12 h post-injection, and single-cell suspensions were prepared. Uptake of PKH67-MEVs by TAMs (F4/80^+^CD11b^+^) and LEVDs by GBM cells (mCherry^+^) was detected by flow cytometry.


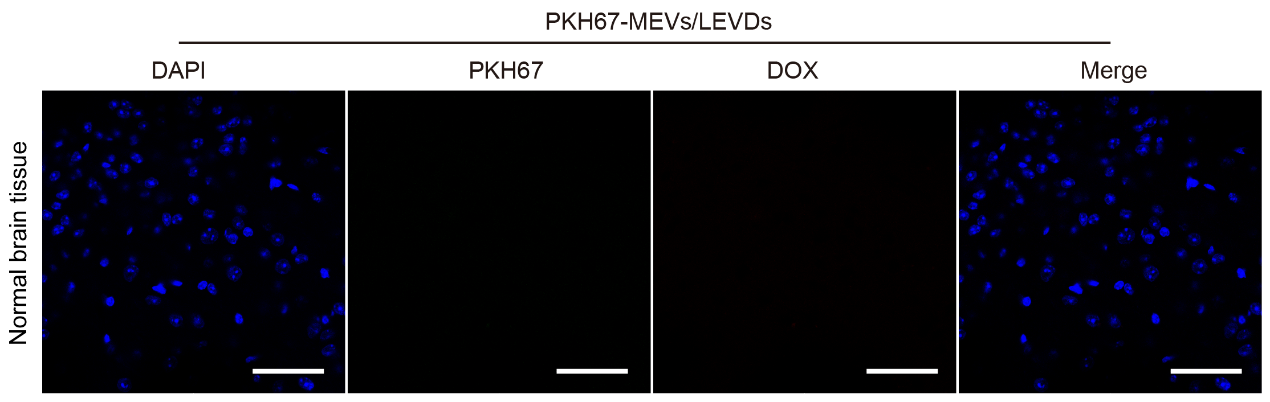


**Fig. S32.** LSCM images of brain tissues from healthy C57BL/6J mice after intravenous injection of MEVs/LEVDs. Scale bar: 50 μm.


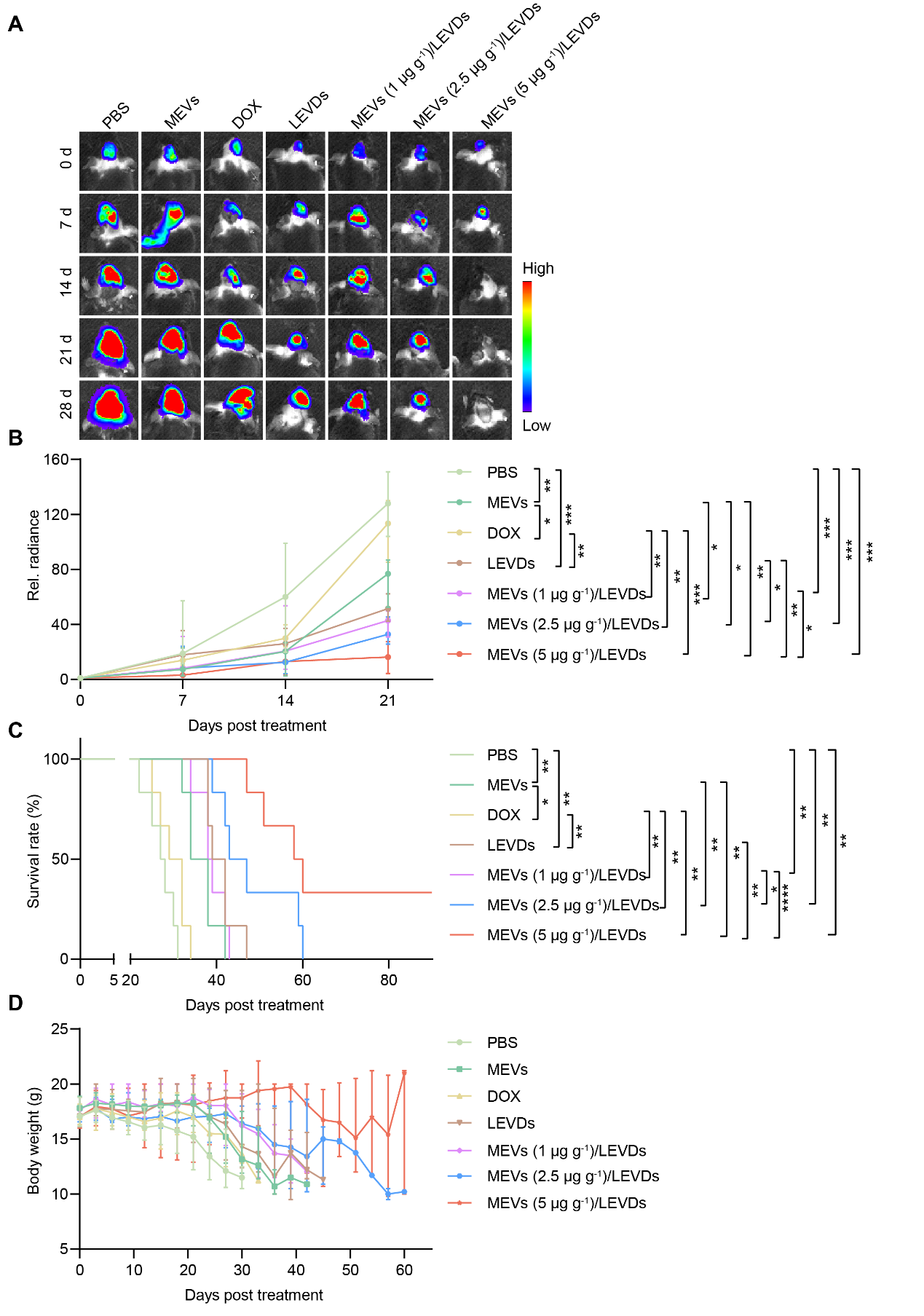


**Fig. S33.** The co-EVs delivery system MEVs/LEVDs exhibits enhanced anti-GBM effects in vivo. (A) Representative bioluminescence IVIS images of GL261-Luc-bearing C57BL/6J mice in different treatment groups. (B) Relative bioluminescence of GL261-Luc tumors in different treatment groups (*n* = 6). (C) Kaplan-Meier survival curves of GL261-Luc-bearing mice (*n* = 6). (D) Body weight changes of GL261-Luc-bearing mice (*n* = 6). Data are represented as mean ± SD. Statistical significance was determined using one-way ANOVA followed by Bonferroni’s post hoc test (B) or two-sided Log-rank Mantel-Cox tests (C), **P* < 0.05; ***P* < 0.01; ****P* < 0.001; *****P* < 0.0001.


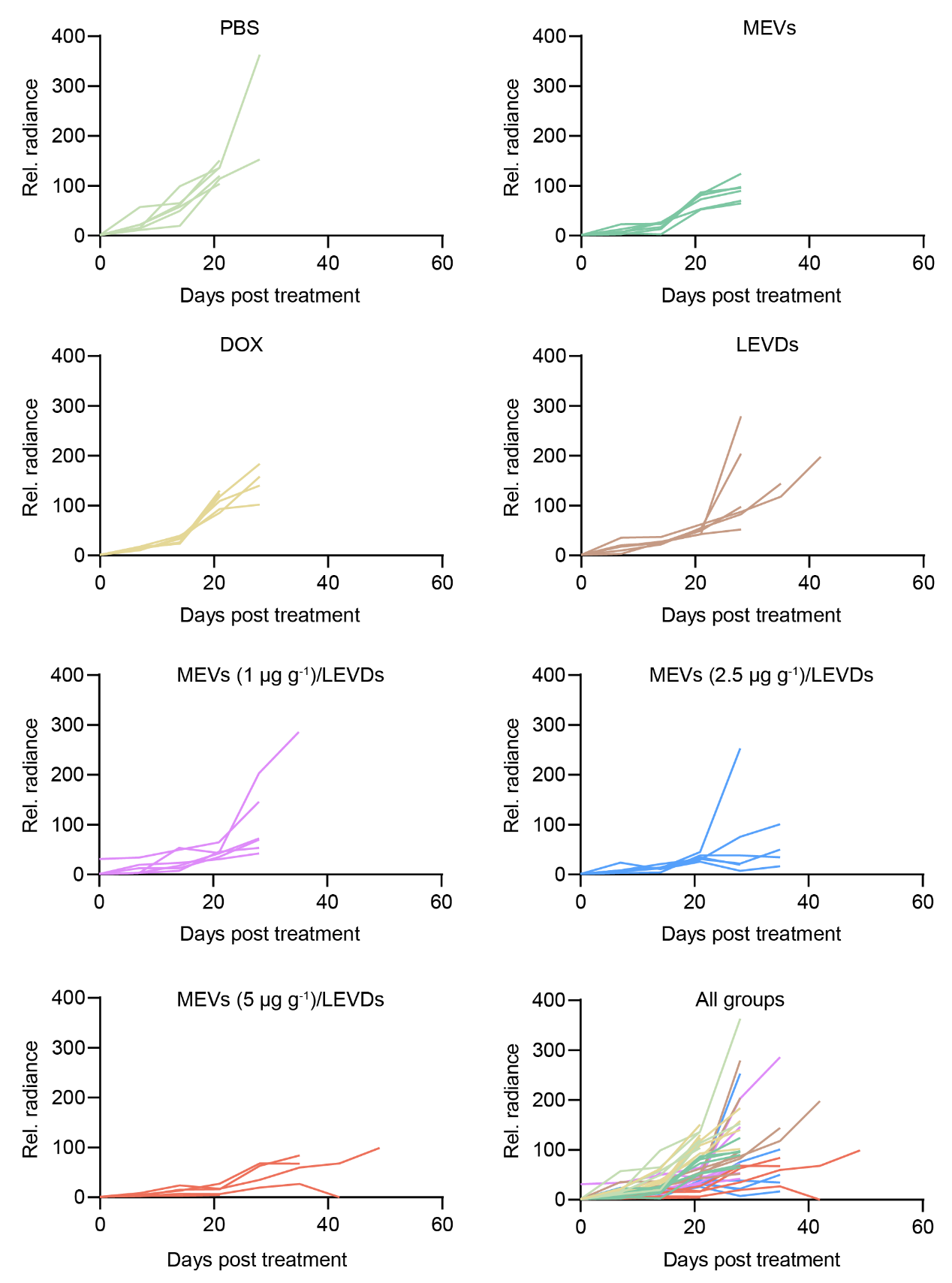


**Fig. S34.** Quantified luminescence of all mice detected by IVIS (*n* = 6).


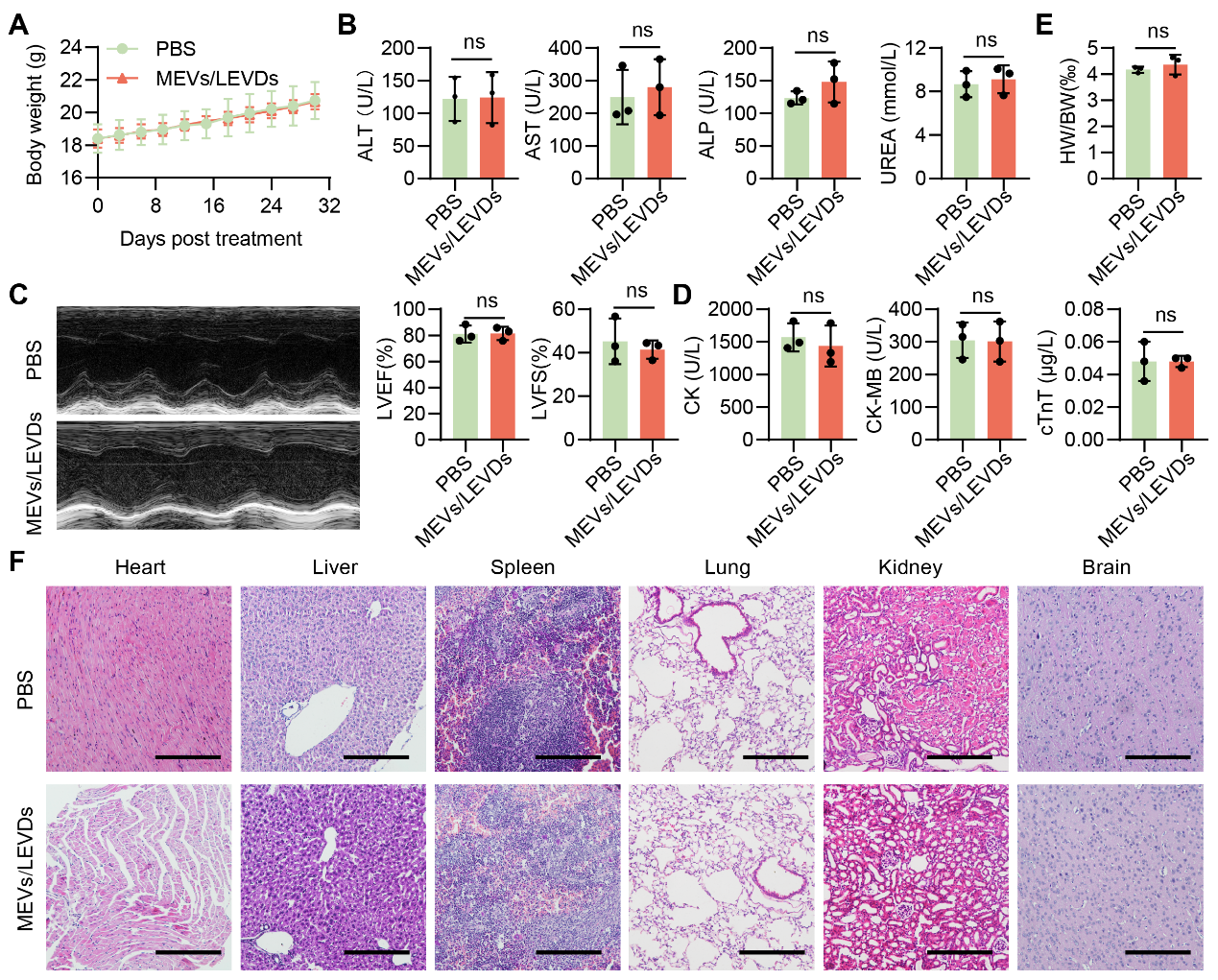


**Fig. S35.** Safety evaluation of in vivo application of co-EVs delivery system MEVs/LEVDs. (A) Body weight of tumor-free C57BL/6J mice treated with PBS or MEVs/LEVDs (*n* = 3). (B) Serum levels of ALT, AST, ALP, and urea (*n* = 3). (C) Left panel: representative echocardiography images; right panel: left ventricular ejection fraction (LVEF) and left ventricular fractional shortening (LVFS) (*n* = 3). (D) Serum levels of CK, CK-MB, and cTnT (*n* = 3). (E) Heart weight/body weight ratio (HW/BW) (*n* = 3). (F) Representative H&E-stained sections of heart, liver, spleen, lung, kidney, and brain. Serum and tissues collected two days after last treatment. Scale bar: 100 μm. Data are represented as mean ± SD. Statistical significance was determined using an unpaired, 2-sided Student’s t test; ns, not significant.


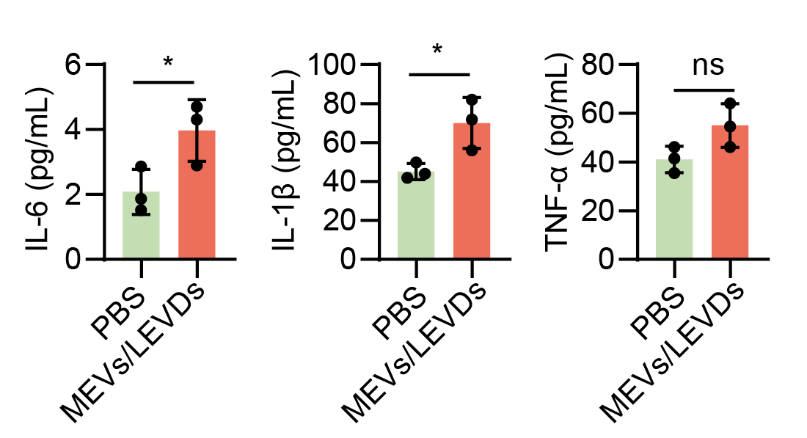


**Fig. S36.** Serum levels of IL-6, IL-1β and TNF-α in tumor-free C57BL/6J mice treated with PBS or MEVs/LEVDs (*n* = 3). Data are represented as mean ± SD. Statistical significance was determined using an unpaired, 2-sided Student’s t test, **P* < 0.05; ns: not significant.


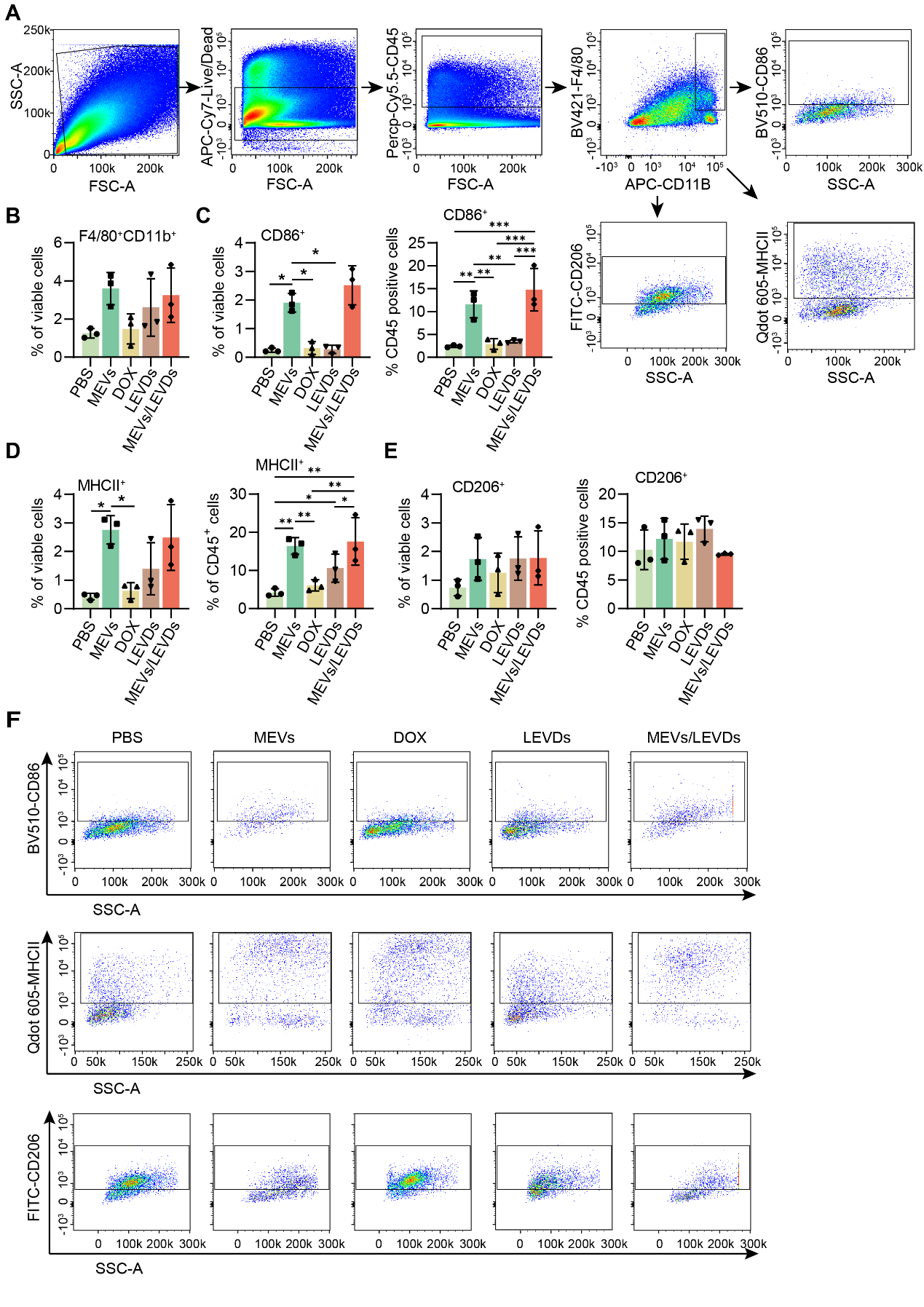


**Fig. S37.** Co-EVs delivery system MEVs/LEVDs repolarizes M2 TAMs to an M1 phenotype in GBM. (A) Gating strategy for flow cytometry analysis of macrophages. Two days after the fifth administration, GBM was processed into single-cell suspension. Live cells were gated, followed by CD45^+^ cells, and then analyzed for M1(CD11b^+^F4/80^+^CD86^+^ or CD11b^+^F4/80^+^MHCII^+^) and M2 (CD11b^+^F4/80^+^CD206^+^) macrophages. (B) Percentage of total macrophages among viable GBM cells (*n* = 3). (C-E) Percentage of M1 and M2 macrophages among viable cells or immune cells in GBM (*n* = 3). (F) Representative flow cytometry plots of M1(CD86^+^ or MHCII^+^) and M2 macrophages (CD206^+^) within CD45^+^CD11b^+^F4/80^+^ cells. Data are represented as mean ± SD. Statistical significance was determined using one-way ANOVA followed by Bonferroni’s post hoc test, **P* < 0.05; ***P* < 0.01; ****P* < 0.001.


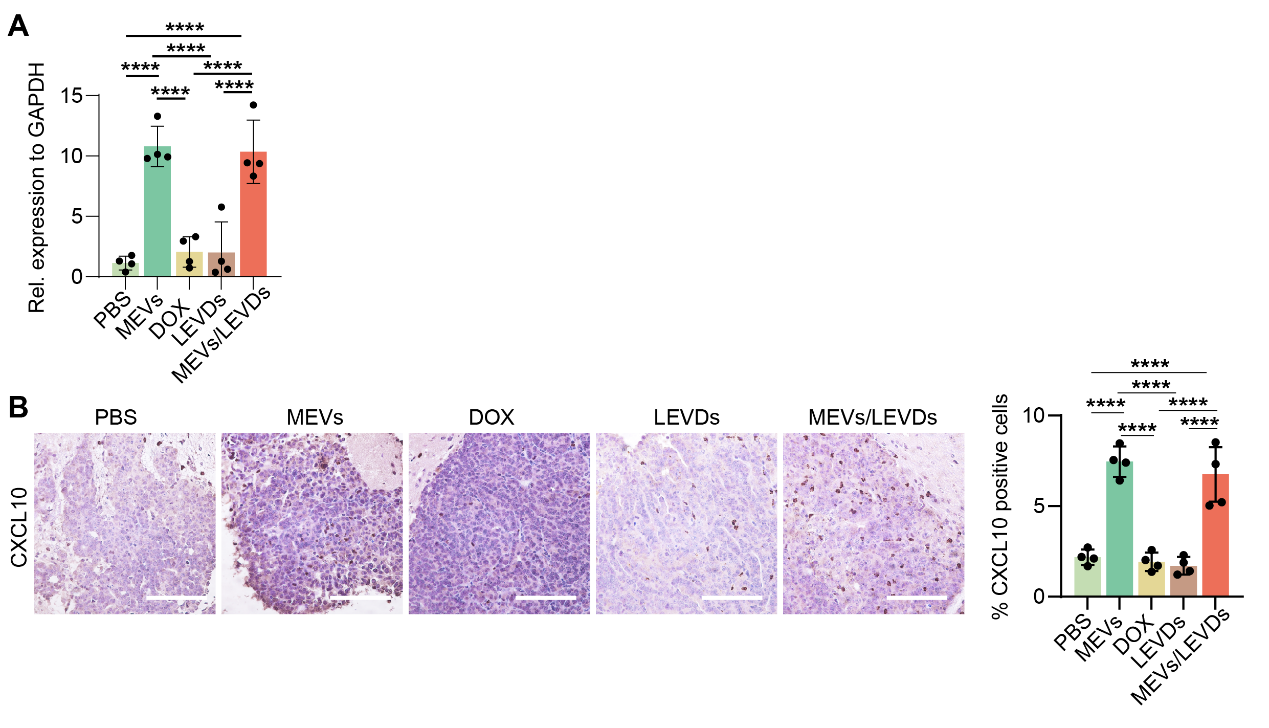


**Fig. S38.** Co-EVs delivery system MEVs/LEVDs increases CXCL10 expression in GBM. (A) qRT-PCR analysis of CXCL10 mRNA in bulk GBM (*n* = 3). (B) IHC analysis of CXCL10 protein in GBM (*n* = 4). Scale bar: 100 μm. Data are represented as mean ± SD. Statistical significance was determined using one-way ANOVA followed by Bonferroni’s post hoc test, *****P* < 0.0001.


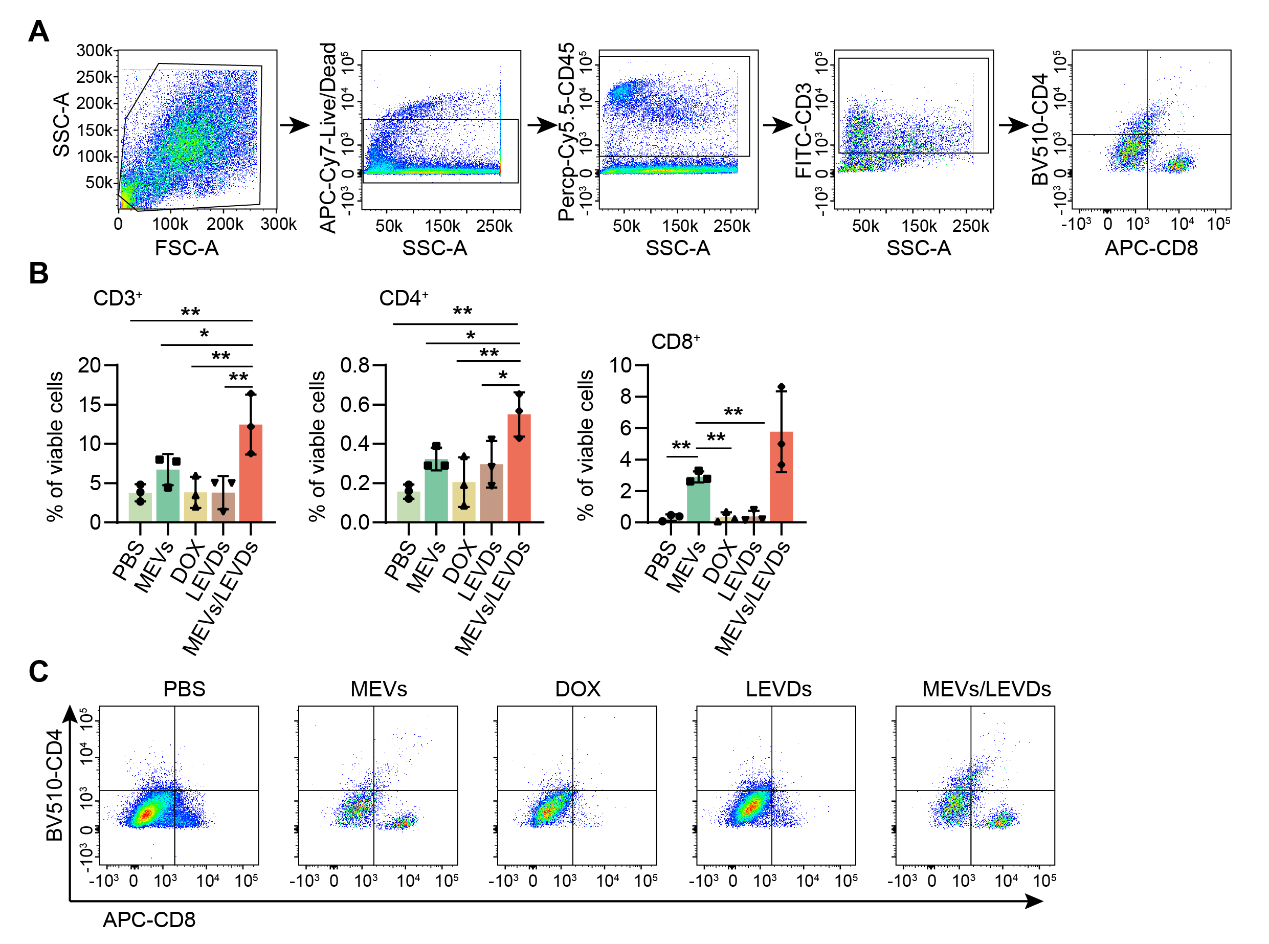


**Fig. S39.** Co-EVs delivery system MEVs/LEVDs increases the infiltration of cytotoxic T-lymphocytes in GBM. (A) Gating strategy for flow cytometry analysis of T cells. Two days after the fifth administration, GBM was processed into single-cell suspension. Live cells were gated, followed by CD45^+^ cells, and then analyzed for T cell markers. (B) Percentage of CD3^+^, CD4^+^ and CD8^+^ T cells in viable GBM cells (*n* = 3). (C) Flow cytometry plots of CD4^+^ and CD8^+^ T cells in CD45^+^CD3^+^ cells of GBM. Data are represented as mean ± SD. Statistical significance was determined using one-way ANOVA followed by Bonferroni’s post hoc test, **P* < 0.05; ***P* < 0.01.


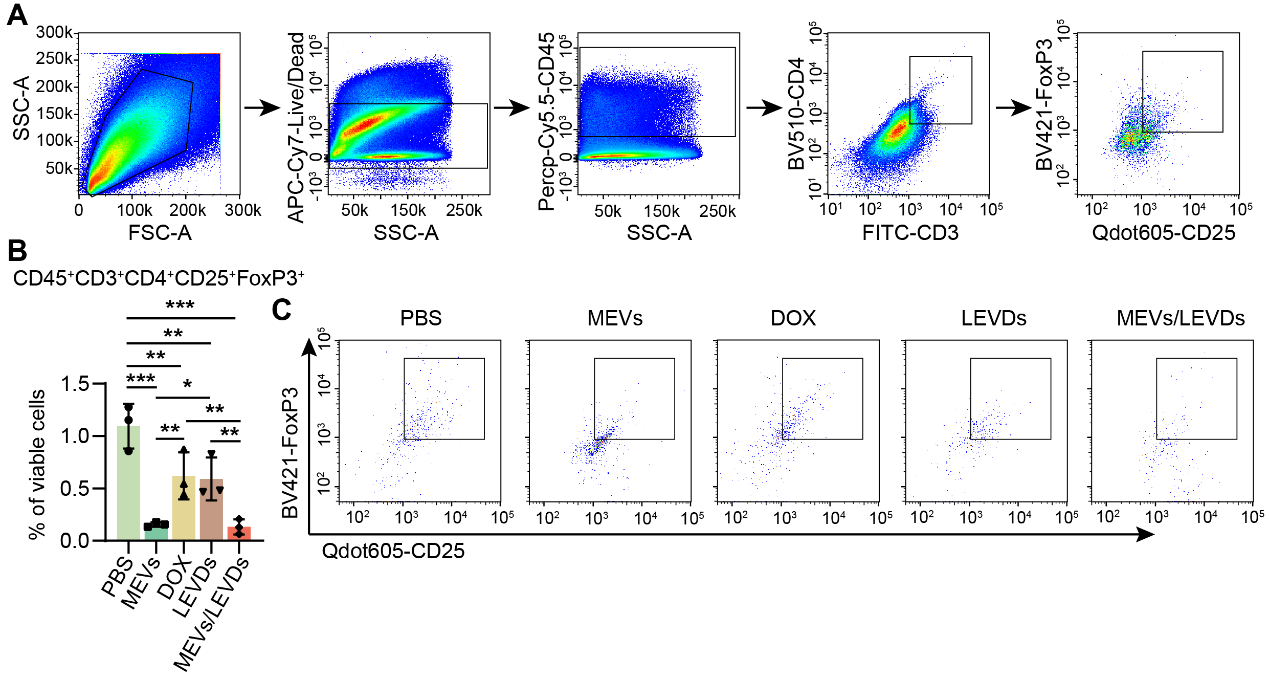


**Fig. S40.** Co-EVs delivery system MEVs/LEVDs decreased the infiltration of Treg lymphocytes in GBM. (A) Gating strategy for flow cytometry analysis of Treg cells. Two days after the fifth administration, GBM was processed into single-cell suspension. Live cells were gated, followed by CD45^+^ cells, and then analyzed for Treg markers. (B) Percentage of Treg cells (CD45^+^CD3^+^CD4^+^CD25^+^FoxP3^+^) in viable GBM cells (*n* = 3). (C) Representative flow cytometry plots of CD25^+^FoxP3^+^ cells within CD45^+^CD3^+^CD4^+^ cells. Data are represented as mean ± SD. Statistical significance was determined using one-way ANOVA followed by Bonferroni’s post hoc test, **P* < 0.05; ***P* < 0.01; ****P* < 0.001.

**Table S1. Sequences of miRNAs used in this study**

| Species | miRNA | sequence 5’-3’ |
| --- | --- | --- |
| Mouse | let-7f-5p | UGAGGUAGUAGAUUGUAUAGUU |
|  | let-7c-5p | UGAGGUAGUAGGUUGUAUGGUU |
|  | miR-423-3p | AGCUCGGUCUGAGGCCCCUCAGU |
|  | let-7i-5p | UGAGGUAGUAGUUUGUGCUGUU |
|  | miR-151-3p | CUAGACUGAGGCUCCUUGAGG |
|  | miR-365-2-5p | AGGGACUUUCAGGGGCAGCUGUG |
|  | miR-99b-3p | CAAGCUCGUGUCUGUGGGUCCG |
|  | let-7a-5p | UGAGGUAGUAGGUUGUAUAGUU |
|  | miR-30c-2-3p | CUGGGAGAAGGCUGUUUACUCU |
|  | let-7b-5p | UGAGGUAGUAGGUUGUGUGGUU |

**Table S2. DOX loading efficiency and loading capacity of LEVDs**

| Batch Number | Loading Efficiency (%) | Loading Capacity (%) |
| --- | --- | --- |
| 1 | 48.37 | 1.56 |
| 2 | 55.82 | 1.67 |
| 3 | 52.29 | 1.64 |
| Mean ± SD | 52.16±3.73 | 1.62±0.06 |

**Table S3. Loading amount of DOX and cRGD per µg (protein) of LEVs in LEVDs**

| Batch Number | Loading weight (µg in per µg of EVs) | |
| --- | --- | --- |
|  | DOX | cRGD peptide |
| 1 | 1.61 | 0.42 |
| 2 | 1.64 | 0.43 |
| 3 | 1.36 | 0.35 |
| Mean ± SD | 1.54±0.16 | 0.40±0.04 |

**Table S4. Primers for qRT-PCR**

| Species | Gene |  | Primer sequence 5’-3’ |
| --- | --- | --- | --- |
| Mouse | CD206 | Forward | CTGCAGATGGGTGGGTTATT |
|  |  | Reverse | GGCATTGATGCTGCTGTTATG |
|  | IL-10 | Forward | GCTCTTACTGACTGGCATGAG |
|  |  | Reverse | CGCAGCTCTAGGAGCATGTG |
|  | CD86 | Forward | GATTATCGGAGCGCCTTTCT |
|  |  | Reverse | CCACACTGACTCTTCCATTCTT |
|  | iNOS | Forward | TCACCTTCGAGGGCAGCCGA |
|  |  | Reverse | TCCGTGGCAAAGCGAGCCAG |
|  | IL-6 | Forward | ATCCAGTTGCCTTCTTGGGACTGA |
|  |  | Reverse | TTGGATGGTCTTGGTCCTTAGCCA |
|  | IL-1β | Forward | GAAATGCCACCTTTTGACAGTG |
|  |  | Reverse | CTGGATGCTCTCATCAGGACA |
|  | TNF-α | Forward | CCTGTAGCCCACGTCGTAGC |
|  |  | Reverse | AGCAATGACTCCAAAGTAGACC |
|  | CXCL10 | Forward | ATCATCCCTGCGAGCCTATCCT |
|  |  | Reverse | GACCTTTTTTGGCTAAACGCTTTC |
|  | GAPDH | Forward | TGTGATGGGTGTGAACCACG |
|  |  | Reverse | CAGTGAGCTTCCCGTTCAGC |
|  | Arg-1 | Forward | TGTCCCTAATGACAGCTCCTT |
|  |  | Reverse | GCATCCACCCAAATGACACAT |
|  | A20 | Forward | ACTGGAATGACGAATGGGACA |
|  |  | Reverse | CAGGGAATTGTACTGAAGTCCAC |
| Human | CD206 | Forward | GGCGGTGACCTCACAAGTAT |
|  |  | Reverse | TTTTCATGGCTTGGTTCTCC |
|  | CD204 | Forward | CCAGGTCCAATAGGTCCTCC |
|  |  | Reverse | CTGGCCTTCCGGCATATCC |
|  | IL-10 | Forward | GACTTTAAGGGTTACCTGGGTTG |
|  |  | Reverse | TCACATGCGCCTTGATGTCTG |
|  | IGF-1 | Forward | GCTCTTCAGTTCGTGTGTGGA |
|  |  | Reverse | GCCTCCTTAGATCACAGCTCC |
|  | TNF-α | Forward | GGCTCCAGGCGGTGCTTG |
|  |  | Reverse | CAGATAGATGGGCTCATACCA |
|  | IL-1β | Forward | ATGATGGCTTATTACAGTGGCAA |
|  |  | Reverse | GTCGGAGATTCGTAGCTGGA |
|  | IL-6 | Forward | ACTCACCTCTTCAGAACGAATTG |
|  |  | Reverse | CCATCTTTGGAAGGTTCAGGTTG |
|  | IL-12 | Forward | CATCAGGGACATCATCAA |
|  |  | Reverse | GTCAGGGAGAAGTAGGAA |
|  | HLA-DR | Forward | ATCATGACAAAGCGCTCCAACTAT |
|  |  | Reverse | GATGCCCACCAGACCCACAG |
|  | GAPDH | Forward | CCACCCATGGCAAATTCC |
|  |  | Reverse | TGGGATTTCCATTGATGACAA |

**Table S5. Antibodies used for western blotting, immunofluorescence (IF), and IHC**

| Antibody | Source | Catalogue  number | Application | Dilution |
| --- | --- | --- | --- | --- |
| CD9 | abcam | ab307085 | Western blotting | 1:1000 |
| TSG101 | abcam | ab125011 | Western blotting | 1:1000 |
| Alix | abcam | ab275377 | Western blotting | 1:1000 |
| iNOS | abcam | ab178945 | Western blotting | 1:1000 |
| GAPDH | Proteintech Group | 10494-1-AP | Western blotting | 1:20000 |
| CD206 | abcam | ab64693 | Western blotting | 1:1000 |
| A20 | Cell Signaling Technology (CST) | #5630 | Western blotting | 1:1000 |
| p-NF-κB p65 (S536) | CST | #3033 | Western blotting | 1:1000 |
| NF-κB p65 | CST | #8242 | Western blotting/IF | 1:1000/1:400 |
| ZO-1 | Proteintech Group | 21773-1-AP | IF | 1:2000 |
| Claudin-1 | Proteintech Group | 13050-1-AP | IF | 1:2000 |
| PECAM-1 | Santa Cruz Biotechnology | sc-376764 | IF | 1:200 |
| iNOS | abcam | ab178945 | IF/IHC | 1:500 |
| CD206 | CST | #24595 | IHC | 1:1000 |
| Ki67 | MXB Biotechnologies | MAB-0672 | IHC | 1:1 |
| CD34 | MXB Biotechnologies | Kit-0004 | IHC | 1:1 |
| CD163 | abcam | ab182422 | IHC | 1:500 |
| CD4 | abcam | ab183685 | IHC | 1:250 |
| CD8 | abcam | ab217344 | IHC | 1:500 |
| FoxP3 | abcam | ab215206 | IHC | 1:400 |
| CXCL10 | Proteintech | 10937-1-AP | IHC | 1:200 |

**Table S6. Antibodies used for flow cytometry**

(Dilutions used as per manufacturer’s recommendations)

| Antibody | Source | Catalogue  number |
| --- | --- | --- |
| PerCP-Cy™5.5 Rat Anti-Mouse CD45 | BD | 561869 |
| CD11b Monoclonal Antibody (M1/70), APC | eBioscience | 17-0112-82 |
| BV 421™ anti-mouse F4/80 | BioLegend | 123132 |
| BV 510™ anti-mouse CD80 | BioLegend | 104741 |
| BV605 Rat Anti-Mouse MHCII | BD | 563413 |
| FITC anti-mouse CD206 (MMR) Antibody | BioLegend | 141704 |
| BV510 Rat Anti-Mouse CD86 | BD | 563077 |
| FITC anti-mouse CD3ε Antibody | BioLegend | 152304 |
| BV 510™ anti-mouse CD4 Antibody | BioLegend | 116025 |
| APC anti-mouse CD8a Antibody | BioLegend | 100712 |
| BV605 Rat Anti-Mouse CD25 | BD | 563061 |
| BV421 Rat Anti-Mouse Foxp3 | BD | 562996 |
| Recombinant Anti-Integrin alpha V antibody | abcam | ab124968 |
| Recombinant Anti-Integrin beta 3 antibody | abcam | ab119992 |
| Anti-Integrin alpha V beta 3 antibody | abcam | ab190147 |
| Goat Anti-Mouse IgG H&L (DyLight® 488) | abcam | ab96871 |
